# Supplementary material for: Bivariate causal mixture model quantifies polygenic overlap between complex traits beyond genetic correlation
Source: Nat Commun. 2019 Jun 3;10:2417. doi: 10.1038/s41467-019-10310-0 (PMC6547727; doi:10.1038/s41467-019-10310-0)
Supplement: Supplementary file 1 — Supplementary Information [file 41467_2019_10310_MOESM1_ESM.pdf]

Supplementary information cover page

Bivariate causal mixture model quantifies polygenic overlap between complex traits beyond genetic correlation

Frei et al.

SUPPLEMENTARY NOTE 1.

Theory. Consider a bi-allelic genetic variant j, and let β_j be the effect size of allele substitution of that variant on a given quantitative trait. Variants with non-zero effect $\beta_j \neq 0$ are said to be *causal* for the trait. We assume a simple additive generative model

$$y_k = \sum_{j=1}^{M} g_{kj}\beta_j + e_k, \tag{1}$$

where y_k is a quantitative trait measured on k-th individual ($k = 1, \ldots, N$), g_{kj} is an additively coded number of reference alleles for j-th variant ($j = 1, \ldots, M$) on k-th individual, β_j is the effect size of variant j, and e_k contains additive environmental and measurement error effects. In a vector form, $\mathbf{y} = \mathbf{G}\beta + \mathbf{e}$, and we denote rows of the genotype matrix $\mathbf{G}$ as $\mathbf{x_k^T}$ (genotype vector for k-th individual) and columns as $\mathbf{v_j}$ (genotype vector for j-th variant).

The scales of β_j and e_k are chosen so that phenotype vector $\mathbf{y}$ has unit variance, $\text{Var}(\mathbf{y}) = 1$, and $\text{Var}(\mathbf{e}) = 1 - h^2$ where h^2 is narrow-sense heritability of the trait. Genotypes g_{kj} are assumed to be centered for each variant to have zero mean across individuals, but are not normalized, therefore $\text{Var}(\mathbf{v_j}) = 2p_j(1-p_j)$, where p_j is minor allele frequency of j-th variant.

Theorem 1. *Let $\hat{\beta}'_j$ be GWAS estimate of j-th effect size, assessed via univariate linear regression, and z_j be corresponding z-score, $z_j = \hat{\beta}'_j / \hat{se}(\beta'_j)$. Then*

$$\begin{aligned} z_j &= \delta_j + \epsilon_j, \\ \delta_j &= \sqrt{N_j} \sum_i \sqrt{H_i} r_{ij} \beta_i, \\ \epsilon_j &\sim \mathcal{N}(0, \sigma_0^2), \end{aligned} \tag{2}$$

where N_j is the number of subjects with non-missing genotype information on j-th variant; $H_i = 2p_i(1 - p_i)$ is heterozygosity of i-th variant; $r_{ij} = corr(\mathbf{v_i}, \mathbf{v_j})$ is an allelic correlation coefficient (LD r2) between genotypes of variants i and j; summation $\sum_i$ runs across all variants i with non-zero r_{ij} with j-th variant, and parameter σ_0^2 accounts for cryptic relatedness among individuals.

In the absence of covariates, the least square estimates can be expressed as

$$\hat{\beta}'_j = \frac{\mathbf{v_j^T y}}{\mathbf{v_j^T v_j}} = \beta_j + \sum_{i \neq j} \hat{\xi}_{ij}\beta_i + \frac{\mathbf{v_j^T e}}{\mathbf{v_j^T v_j}},$$

where $\hat{\xi}_{ij} = \mathbf{v_i^T v_j}/\mathbf{v_j^T v_j} = \hat{\zeta}_{ij}/\hat{\zeta}_{jj}$, with $\hat{\zeta}_{ij} = \mathbf{v_i^T v_j}/N$ being an estimate of the covariance between i-th and j-th variants:

$$\hat{\zeta}_{ij} \simeq \sqrt{2p_i(1 - p_i)}\sqrt{2p_j(1 - p_j)} r_{ij}.$$

Here the symbol "$\simeq$" denotes asymptotic equality as $n \to \infty$. Then using $\hat{se}(\beta'_j) = \sqrt{H_j/N_j}$ result in (2). ■

Univariate Causal Mixture model for GWAS. In unvariate case we model β_j as a mixture of null and non-null components:

$$\beta_j \sim (1-\pi_1)\mathcal{N}(0,0) + \pi_1\mathcal{N}(0,\sigma^2_\beta), \tag{3}$$

where π_1 is the proportion of causal variants, σ^2_β is the "discoverability" (phenotypic variance explained per causal variant), and $\mathcal{N}(0,0)$ is a Dirac delta function with the entire probability mass concentrated as 0. All parameters are assumed to be equal across all variants. We are interested in finding parameters π_1, σ^2_β and σ^2_0 that best describe the observed values of z_j in GWAS summary statistics. For this we need likelihood term $pdf(z_j|\pi_1,\sigma^2_\beta,\sigma^2_0)$, where z_j is given by (2) with β_j distributed according to (3):

$$z_j|\pi_1,\sigma^2_\beta,\sigma^2_0 \sim \sum_i \Big[(1-\pi_1)\mathcal{N}(0,0) + \pi_1\mathcal{N}(0, N_j H_i r^2_{ij}\sigma^2_\beta)\Big] + \mathcal{N}(0,\sigma^2_0), \tag{4}$$

where the outer sumation $\sum_i$ denotes a sum of random variables, then each summand is a mixture of two distributions, finally the last plus sign again indicates sum of random variables (e.i. convolution of corresponding probability density functions).

Below we focus on a computationally efficient way of calculating likelihood from expression (4). This task is not straighforward because (4) involves multiple convolutions of a mixture distribution. First we simplify expression (4) by assuming that regardless of index i all r^2_{ij} values are equal to a certain parameter κ^2_j, and also assuming equal heterozigosity of all variants (for all i, $H_i = H_j$). Then we relax this assumption and derive general formulas for 2nd and 4th raw moments of $z_j|\pi_1,\sigma^2_\beta,\sigma^2_0$. Finally, we derive two computationally efficient approximations, one based on two-component Gaussian mixture, and another based on Poisson approximation to binomial distribution. In the Online methods, the two-component Gaussian mixture is referred to as "fast" model, and it is used to perform initial search in the space of models parameters. The Poisson approximation is not used directly, but it provides an important insight into mathematics of GWAS z scores in a context of the casal mixture model.

Lemma 2. *For a given variant j assume all non-zero allelic correlations r^2_{ij} are equal to a certain value κ^2_j, and assume equal heterozigosity of all variants (for all i, $H_i = H_j$). Then*

$$pdf(z_j|\pi_1,\sigma^2_\beta,\sigma^2_0) = \sum_{k=0}^{L_j} \binom{L_j}{k} \pi_1^k (1-\pi_1)^{L_j-k} \phi(z_j; 0, \sigma^2_0 + k\kappa^2_j H_j N_j \sigma^2_\beta), \tag{5}$$

where $\ell_j = \sum_i r^2_{ij}$ indicates total LD score of variant j, and $L_j = \ell_j/\kappa^2_j$ denote the number of variants in LD with variant j.

Proof. Consider two-component mixture of random variables A and B with weights p and q (where $p+q=1$). L-times convolution of this mixture with itself is given by the following combinatorial expression:

$$\sum_{i=1}^{L}(pA+qB) = \sum_{k=0}^{L}\binom{L}{k} p^k q^{L-k}\Big(kA + (L-k)B\Big).$$

Applying this formula to (4) with $p=\pi_1$, $q = 1-\pi_1$, $A = \mathcal{N}(0, H_j N_j \sigma^2_\beta)$ and $B = \mathcal{N}(0,0)$ concludes the proof. ■

With $\pi_1 = 1$ expression (5) simplifies to $\mathcal{N}(0, \sigma_0^2 + \ell_j H_j N_j \sigma_\beta^2)$, which corresponds to the "infinitesimal" model underlying LD score regression.

Now we aim to relax the assumptions of $r_{ij}^2 = \kappa_j^2$ and $H_i = H_j$, and, for the general case, derive expressions for the 2nd and 4th raw moments $E(z_j^2)$ and $E(z_j^4)$. First we show how they are related to the raw moments of δ_j.

Lemma 3.

$$E(z_j^2) = E(\delta_j^2) + \sigma_0^2,$$
$$E(z_j^4) - 3(E(z_j^2))^2 = \Big(E(\delta_j^4) - 3\big(E(\delta_j^2)\big)^2\Big).$$

Proof. Expression for $E(z_j^2)$ straighforwardly follows from (2). To prove the second formula we note that for two independent random variables x and y the expectation $E(x+y)^4 = Ex^4+3Ex^3Ey+6Ex^2Ey^2+3ExEy^3+Ey^4$, which simplifies to $E(x+y)^4 = Ex^4 + 6Ex^2Ey^2 + Ey^4$ for zero mean random variables. Thus

$$E(z_j^4) = E(\delta_j^4) + 6E(\delta_j^2)\sigma_0^2 + 3\sigma_0^4,$$

and hence $E(z_j^4) - 3(E(z_j^2))^2 = (N_j H_j)^2 \Big(E(\beta_j'^4) - 3\big(E(\beta_j'^2)\big)^2\Big)$. ■

Last lemma allows us to simplify notation and derive all formulas in terms of $E(\delta_j^2)$ and $E(\delta_j^4)$, which depend only on π_1 and σ_β^2, but not on σ_0^2.

Lemma 4.

$$E(\delta_j^2) = N_j \ell_j \pi_1 \sigma_\beta^2,$$
$$E(\delta_j^4) - 3E(\delta_j^2)^2 = 3N_j^2 R_j \pi_1 \pi_0 \sigma_\beta^4, \tag{6}$$

where $R_j = \sum_i H_i^2 r_{ij}^4$ is a sum of fourth power of allelic correlations (adjusted for heterozigosity), and $\ell_j = \sum_i H_i r_{ij}^2$ is total LD score (adjusted for heterozigosity).

Proof. From (2), $\delta_j/N_j = \sum_i \sqrt{H_i} r_{ij} \beta_i$, where all β_i are independent equally distributed random variables with $E(\beta_i^2) = \pi_1 \sigma_\beta^2$ and $E(\beta_i^4) = 3\pi_1 \sigma_\beta^4$.

$$E(\delta_j^2/N_j) = E\Big(\sum_i \sqrt{H_i} r_{ij} \beta_i\Big)^2 = \sum_i H_i r_{ij}^2 E(\beta_i^2) + \sum_{i<k} 2\sqrt{H_i H_k} r_{ij} r_{kj} E(\beta_i) E(\beta_k).$$

The second sum is equal to zero because $E(\beta_i) = 0$. Thus,

$$E(\delta_j^2/N_j) = \sum_i H_i r_{ij}^2 E(\beta_i^2) = \ell_j E(\beta^2) = \ell_j \pi_1 \sigma_\beta^2.$$

Similarly, for $E(\delta_j^4/N_j^2)$ we get

$$E(\delta_j^4/N_j^2) = E\Big(\sum_i \sqrt{H_i} r_{ij} \beta_i\Big)^4 = \sum_i H_i^2 r_{ij}^4 E(\beta_i^4) + \sum_{i<k} 6 H_i H_k r_{ij}^2 r_{kj}^2 E(\beta_i^2) E(\beta_k^2),$$

where all summands with zero mean are already omited. Note that

$$2\sum_{i<k} H_i H_k r_{ij}^2 r_{kj}^2 = \Big(\sum_i H_i r_{ij}^2\Big)^2 - \sum_i H_i^2 r_{ij}^4 = \ell_j^2 - R_j,$$

hence $E(\delta_j^4) = N_j^2 R_j E(\beta^4) + 3N_j^2(\ell_j^2 - R_j)E(\beta^2)^2$, and thus

$$E(\delta_j^4) - 3E(\delta_j^2)^2 = N_j^2 R_j \Big(E(\beta^4) - 3N_j^2 E(\beta^2)^2\Big) = 3N_j^2 R_j(\pi_1 - \pi_1^2)\sigma_\beta^4.$$

■

Corollary 4.1. *An arbitrary LD structure of a j-th variant could be approximated via "spike"-like histogram of equal r_{ij} values, with effective allelic correlation $\hat{\kappa}_j^2 = R_j/\ell_j$, and effective block size $\hat{L}_j = \ell_j^2/R_j$, and such approximation preserves 2nd and 4th moments of z_j.*

Proof. The statement directly follows from lemmas 3 and 4. ■

Last corolary also imply that the variance of z_j depends only on the total LD score ℓ_j, and does not depend on κ_j^2 parameter. On contrary, the excess kurtosis linearly depend on κ_j^2. Recall that the excess kurtosis is a measure of how heavy are the tails of the distribution. This means that in the context of a mixture model total LD score itself is not sufficient to describe the distribution of z scores, because variants with higher κ_j^2 will tend to have larger z scores despite having the same total LD score.

Last corollary alloes one to approximate likelihood $pdf(z|\pi_1, \sigma_\beta^2, \sigma_0^2)$ with binomial formula (5), with parameters $\kappa_j = R_j/\ell_j$, $H_j = 1$, and $L_j = \ell_j^2/R_j$. Such approximation is reasonable in a sense that it preserves 2nd and 4th moments of z_j.

Likelihood calculation from (5) still involves a potentially large sum $\sum_{k=0}^{L}$. To further speedup calculations we show that a two-component mixture of Gaussin distributions has enough flexibility to preserve 2nd and 4th moments of z_j.

Theorem 5 (Gaussian approximation)**.** *Let*

$$z_j' \sim \pi_{0j}'\mathcal{N}(0, \sigma_0^2) + \pi_{1j}'\mathcal{N}(0, \sigma_0^2 + \sigma_{\delta_j}^2), \tag{7}$$

where

$$\begin{aligned} \pi_{1j}' &= \frac{\pi_1 \ell_j}{\pi_1 \ell_j + \pi_0 \kappa_j^2}, \\ \sigma_{\delta_j}^2 &= N_j \sigma_\beta^2 (\pi_0 \kappa_j^2 + \pi_1 \ell_j), \\ \ell_j &= \sum_i H_i r_{ij}^2, \quad R_j = \sum_i H_i^2 r_{ij}^4, \\ \kappa_j^2 &= R_j/\ell_j, \quad \pi_0 = 1 - \pi_1, \quad \pi_{0j}' = 1 - \pi_{1j}'. \end{aligned} \tag{8}$$

Then 2nd and 4th moments of z_j' and z_j distributions are equal.

Proof. Lemmas 3 and 4 derive expressions for 2nd and 4th moments of z_j. What's left is to find same moments for z_j', defined by (7):

$$\begin{aligned} E(z_j'^2) &= \pi_{1j}' \sigma_{\delta_j}^2 + \sigma_0^2, \\ E(z_j'^4) - 3(E(z_j'^2))^2 &= \pi_{1j}' \pi_{0j}' \sigma_{\delta_j}^4, \end{aligned}$$

Using (8) and doing straighforward algebraic computations concludes the proof. ■

Gaussian approximation provides very efficient way of calculating likelihood $pdf(z_j|\pi_1, \sigma^2_\beta, \sigma^2_0)$. As a downside, such two-component gaussian mixture does not fully capture heavy tails of z_j distribution — even though both 2nd and 4th moments are preserved. The following approximation, which we call *poisson-type approximation*, gives more accurate representation for the tails of z-score distribution. A key feature of poisson-type approximation is that it has the same underlying structure as the binomial formula (5), e.i. it is a mixture of gaussians with linearly increasing variance ($k\sigma_j^2$, $k = 0, 1, 2, \dots,$).

Theorem 6 (Poisson-type approximation)**.** *Let*

$$z''_j \sim \sum_{k=0}^{\infty} \frac{\lambda_j^k e^{-\lambda_j}}{k!} \mathcal{N}(0, \sigma_0^2 + k\sigma_j^2), \tag{9}$$

where

$$\begin{aligned} \lambda_j &= (\pi_1 \ell_j^2)/(\pi_0 R_j), \\ \sigma_j^2 &= N_j \sigma_\beta^2 \pi_0 R_j / \ell_j. \end{aligned} \tag{10}$$

Then 2nd and 4th moments of z''_j and z_j distributions are equal.

Proof. Lemmas 3 and 4 derive expressions for 2nd and 4th moments of z_j. What's left is to find same moments for z''_j. Let's denote $p_k = \frac{\lambda_j^k e^{-\lambda_j}}{k!}$. Then,

$$E(z''^2) = \sigma_0^2 + \sigma_j^2 \sum_{k=0}^{\infty} k p_k = \sigma_0^2 + \sigma_j^2 \lambda_i.$$

$$E(z''^4) - (E(z''^2))^2 = 3\sigma_0^4 + 3\sigma_j^4 \Big(\sum_{k=0}^{\infty} k^2 p_k - \Big(\sum_{k=0}^{\infty} k p_k \Big)^2 \Big) = 3\sigma_j^4 \lambda_j.$$

Using (10) and doing straighforward algebraic computations concludes the proof. ∎

In practice the invfinite sum in (9) converges very quickly, typically $k_{max} = 5$ is sufficient. In addition, σ_j^2 can be adjusted by $poisscdf(k_{max-1}, \lambda)$ factor, where *poisscdf* gives cumulated distribution function of the discrete Poisson distribution. Such adjustement compensates for variance explained by $k > k_{max}$.

To get an improved fit of the model to the data one may split LD structure of j-th variant into regions with low r_{ij}^2 and high r_{ij}^2, each approximated with own (λ_j, σ_j^2) values, This can be handled using the following analytical way of convolving two Poisson-type approximations:

$$z''_j \sim \sum_{k_1=0}^{\infty} \sum_{k_2=0}^{\infty} \frac{\lambda_{1j}^{k_1} e^{-\lambda_{1j}}}{k_1!} \frac{\lambda_{2j}^{k_2} e^{-\lambda_{2j}}}{k_2!} \mathcal{N}(0, \sigma_0^2 + (k_1 \sigma_{1j}^2 + k_2 \sigma_{2j}^2)).$$

This formulas simplify considerably if $\sigma_{1j}^2 = \sigma_{2j}^2 = \sigma_j^2$. If that's the case, we have

$$z''_j \sim \sum_k (f * g)_k \mathcal{N}(0, \sigma_0^2 + k\sigma_j^2), \tag{11}$$

where $f_k = \frac{\lambda_{1j}^{k_1} e^{-\lambda_{1j}}}{k_1!}$, $g_k = \frac{\lambda_{2j}^{k_2} e^{-\lambda_{2j}}}{k_2!}$, and $(f * g)_k = \sum_m f_m g_{k-m}$ denotes discrete confolution of coefficients f_k and g_k.

Bivariate causal mixture model for GWAS. For a given individual, simple additive genetic model for two quantitative traits y_1, y_2 can be written as follows:

$$\begin{aligned} y_1 &= \sum_{j=1}^{M} g_j \beta_{1j} + e_1, \\ y_2 &= \sum_{j=1}^{M} g_j \beta_{2j} + e_2, \end{aligned} \tag{12}$$

where g_j is an additively coded number of reference alleles for j-th variant, β_{1j} (β_{2j}) is the effect of j-th variant on, respectively, trait y_1 (y_2), and e_1 (e_2) contains additive environmental and measurement error effects. Here we choose that random variables y_1, β_{1j} and e_1 are centered and scaled so that $E(y_1) = 0$ and $\text{Var}(y_1) = 1$, and likewise for the second trait. We allow for dependencies between genetic effects for same variant (β_{1j}, β_{2j}), and also allow for dependency between environmental components (e_1, e_2). Apart from these dependencies, we model genetic effects β_{1j} and β_{2k} ($j \neq k$) independently from each other, and independently from the environmental terms e_1, e_2.

For each trait, it's *heritability* is defined as the variance of the genetic component:

$$h_1^2 = \frac{\text{Var}(\sum_{j=1}^{M} g_j \beta_{1j})}{\text{Var}(y)} = \sum_{j=1}^{M} H_j \text{Var}(\beta_{1j}), \tag{13}$$

where $H_j = 2p_j(1 - p_j)$ is heterozigosity of j-th variant. Likewise, for the second trait $h_2^2 = \sum_{j=1}^{M} H_j \text{Var}(\beta_{2j})$. *Genetic correlation* between the traits is defined as the covariance of the genetic components, normalized by $\sqrt{h_1^2 h_2^2}$:

$$\rho_g = \frac{\text{Var}(\sum_{j=1}^{M} g_j \beta_{1j}, \sum_{j=1}^{M} g_j \beta_{2j})}{\sqrt{h_1^2 h_2^2}} = \frac{1}{\sqrt{h_1^2 h_2^2}} \sum_{j=1}^{M} H_j \text{Cov}(\beta_{1j}, \beta_{2j}). \tag{14}$$

Bivariate MiXeR model is defined as follows:

$$\begin{aligned} &(\beta_{1j}, \beta_{2j}) \sim \pi_0 \mathcal{N}(0, 0) + \pi_1 \mathcal{N}(0, \mathbf{\Sigma}_1) + \pi_2 \mathcal{N}(0, \mathbf{\Sigma}_2) + \pi_{12} \mathcal{N}(0, \mathbf{\Sigma}_{12}), \\ &\mathbf{\Sigma}_1 = \begin{bmatrix} \sigma_1^2 & 0 \\ 0 & 0 \end{bmatrix}, \mathbf{\Sigma}_2 = \begin{bmatrix} 0 & 0 \\ 0 & \sigma_2^2 \end{bmatrix}, \text{ and } \mathbf{\Sigma}_{12} = \begin{bmatrix} \sigma_1^2 & \rho_{12}\sigma_1\sigma_2 \\ \rho_{12}\sigma_1\sigma_2 & \sigma_2^2 \end{bmatrix}, \\ &(z_{1j}, z_{2j}) \sim (\delta_{1j}, \delta_{2j}) + \mathcal{N}\Big(0, \mathbf{\Sigma}_0\Big), \quad \delta_{\cdot j} = \sqrt{N_{\cdot j}} \sum_i \sqrt{H_i} r_{ij} \beta_{\cdot i}, \\ &\mathbf{\Sigma}_0 = \begin{bmatrix} \sigma_{01}^2 & \rho_0 \sigma_{01}\sigma_{02} \\ \rho_0 \sigma_{01}\sigma_{02} & \sigma_{02}^2 \end{bmatrix}. \end{aligned} \tag{15}$$

We denote the vector of nine parameters of the model by $\theta = (\pi_1, \pi_1, \pi_{12}, \sigma_1^2, \sigma_2^2, \rho_{12}, \sigma_{01}^2, \sigma_{02}^2, \rho_0)$. Below we derive a computationally tractable approximation for the likelihood term $pdf(z_{1j}, z_{2j}|\theta)$, which allows us to fit estimated parameters $\hat{\theta}$ from GWAS summary statistics (z_{1j}, z_{2j}) by maximizing log-likelihood, weighted by $w_j = 1/\ell_j$ or by weights based on random pruning to avoid over-counting in large LD blocks:

$$F(\theta) = \sum_j w_j \log pdf(z_{1j}, z_{2j}|\theta) \to \max_\theta.$$

First, we assume that parameters π_1, π_2 and π_{12} are small ($\pi_1 \ll 1$, $\pi_2 \ll 1$, $\pi_{12} \ll 1$). Therefore (β_{1j}, β_{2j}) can be approximated as a convolution of three mixtures of null and non-null components:

$$\begin{aligned}(\beta_{1j}, \beta_{2j}) \approx &\Big[(1-\pi_1)\mathcal{N}(0,0) + \pi_1\mathcal{N}(0,\mathbf{\Sigma}_1)\Big] + \\ &\Big[(1-\pi_2)\mathcal{N}(0,0) + \pi_2\mathcal{N}(0,\mathbf{\Sigma}_2)\Big] + \\ &\Big[(1-\pi_{12})\mathcal{N}(0,0) + \pi_{12}\mathcal{N}(0,\mathbf{\Sigma}_{12})\Big].\end{aligned} \tag{16}$$

Second, we assume that LD operator $\mathcal{L}_j = \sum_i \sqrt{H_i} r_{ij}$ is commutative with convolutions in (16). This assumption is accurate for the first two components, π_1 and π_2, and is approximate for the last component π_{12}:

$$\begin{aligned}\Big(\frac{\delta_{1j}}{\sqrt{N_{1j}}}, \frac{\delta_{2j}}{\sqrt{N_{2j}}}\Big) = \mathcal{L}_j\Big(\beta_{1j}, \beta_{2j}\Big) \approx & \\ &\mathcal{L}_j\Big[(1-\pi_1)\mathcal{N}(0,0) + \pi_1\mathcal{N}(0,\mathbf{\Sigma}_1)\Big] + \\ &\mathcal{L}_j\Big[(1-\pi_2)\mathcal{N}(0,0) + \pi_2\mathcal{N}(0,\mathbf{\Sigma}_2)\Big] + \\ &\mathcal{L}_j\Big[(1-\pi_{12})\mathcal{N}(0,0) + \pi_{12}\mathcal{N}(0,\mathbf{\Sigma}_{12})\Big].\end{aligned} \tag{17}$$

Now we can use Gaussian-type approximation (Theorem 5) or Poisson-type approximation (Theorem 6) to apply LD operator $\mathcal{L}_j$ to each two-component mixture. For Gaussian-type approximation, the result is as follows:

$$\begin{aligned}\Big(\frac{\delta_{1j}}{\sqrt{N_{1j}}}, \frac{\delta_{2j}}{\sqrt{N_{2j}}}\Big) \approx &\Big[(1-\pi'_{1j})\mathcal{N}(0,0) + \pi'_{1j}\mathcal{N}(0,\mathbf{\Sigma}'_{1j})\Big] + \\ &\Big[(1-\pi'_{2j})\mathcal{N}(0,0) + \pi'_{2j}\mathcal{N}(0,\mathbf{\Sigma}'_{2j})\Big] + \\ &\Big[(1-\pi'_{12j})\mathcal{N}(0,0) + \pi'_{12j}\mathcal{N}(0,\mathbf{\Sigma}'_{12j})\Big],\end{aligned} \tag{18}$$

where

$$\begin{aligned}\pi'_{cj} &= \frac{\pi_c \ell_j}{\pi_c \ell_j + (1-\pi_c)\kappa_j^2}, \quad c \in \{1, 2, 12\}, \\ \mathbf{\Sigma}'_{cj} &= \mathbf{\Sigma}_c\big((1-\pi_c)\kappa_j^2 + \pi_c \ell_j\big), \\ \ell_j &= \sum_i H_i r_{ij}^2, \quad R_j = \sum_i H_i^2 r_{ij}^4, \quad \kappa_j^2 = R_j/\ell_j.\end{aligned} \tag{19}$$

Ignoring interactions between non-zero components in each mixture, (18) can be approximated by

$$\begin{aligned}\Big(\frac{\delta_{1j}}{\sqrt{N_{1j}}}, \frac{\delta_{2j}}{\sqrt{N_{2j}}}\Big) &\approx \pi'_{0j}\mathcal{N}(0,0) + \pi'_{1j}\mathcal{N}(0,\mathbf{\Sigma}'_{1j}) + \pi'_{2j}\mathcal{N}(0,\mathbf{\Sigma}'_{2j}) + \pi'_{12j}\mathcal{N}(0,\mathbf{\Sigma}'_{12j}), \\ \pi'_{0j} &= 1 - \pi'_{1j} - \pi'_{2j} - \pi'_{12j}.\end{aligned} \tag{20}$$

More accurately (18) can be written as a mixture of 8 components:

$$
\begin{aligned}
\Big(\frac{\delta_{1j}}{\sqrt{N_{1j}}}, \frac{\delta_{2j}}{\sqrt{N_{2j}}}\Big) \approx\ & \bar{\pi}'_{1j}\bar{\pi}'_{2j}\bar{\pi}'_{12j}\mathcal{N}(0,0) + \\
& \pi'_{1j}\bar{\pi}'_{2j}\bar{\pi}'_{12j}\mathcal{N}(0,\mathbf{\Sigma}'_{1j}) + \\
& \bar{\pi}'_{1j}\pi'_{2j}\bar{\pi}'_{12j}\mathcal{N}(0,\mathbf{\Sigma}'_{2j}) + \\
& \bar{\pi}'_{1j}\bar{\pi}'_{2j}\pi'_{12j}\mathcal{N}(0,\mathbf{\Sigma}'_{12j}) + \\
& \pi'_{1j}\pi'_{2j}\bar{\pi}'_{12j}\mathcal{N}(0,\mathbf{\Sigma}'_{1j}+\mathbf{\Sigma}'_{2j}) + \\
& \pi'_{1j}\bar{\pi}'_{2j}\pi'_{12j}\mathcal{N}(0,\mathbf{\Sigma}'_{1j}+\mathbf{\Sigma}'_{12j}) + \\
& \bar{\pi}'_{1j}\pi'_{2j}\pi'_{12j}\mathcal{N}(0,\mathbf{\Sigma}'_{2j}+\mathbf{\Sigma}'_{12j}) + \\
& \pi'_{1j}\pi'_{2j}\pi'_{12j}\mathcal{N}(0,\mathbf{\Sigma}'_{1j}+\mathbf{\Sigma}'_{2j}+\mathbf{\Sigma}'_{12j}),
\end{aligned} \tag{21}
$$

where $\bar{\pi}'_{cj} = 1 - \pi'_{cj}$.

To speedup computation of bivariate normal zero-mean denisty one may take advantage of closed-form expression for the matrix inverse. Let variance matrix be $\mathbf{\Sigma} = \begin{bmatrix} a & b \\ b & c \end{bmatrix}$, then the precision matrix $\mathbf{\Sigma}^{-1} = \frac{1}{ac-b^2}\begin{bmatrix} c & -b \\ -b & a \end{bmatrix}$, and bivariate normal density function

$$
\phi(z_1, z_2; 0, \begin{bmatrix} a & b \\ b & c \end{bmatrix}) = \frac{1}{2\pi\sqrt{ac-b^2}} \exp\Big(-\tfrac{1}{2}\frac{cz_1^2 + az_2^2 - 2bz_1z_2}{ac-b^2}\Big). \tag{22}
$$

GWAS power curves. We are interested in calculating the proportion $S(N)$ of SNP heritability captured by genome-wide significant hits, as a function of GWAS sample size N. The $S(N)$ is defined in Online Methods:

$$
\begin{aligned}
S(N) &= \frac{\sum_j \int_{z:\,|z|\geq z_t} C_2(z,N,j)dz}{\sum_j \int_z C_2(z,N,j)dz}, \\
C_q(z,N,j) &= \int \delta^q P(z|\delta)P(\delta,j)d\delta.
\end{aligned} \tag{23}
$$

Whenever δ_j is modeled as mixture of normal distributions, it is possible to calculate the above integrals as an analytical expression. Let $\delta_j \sim \frac{1}{K}\sum_k N(0, S_{kj}^2)$, where S_{kj}^2 is typically a function of N, and $z_j \sim \frac{1}{K}\sum_k N(0, \sigma_0^2 + S_{kj}^2)$. Then

$$
\begin{aligned}
C_2(z,N,j) &= \frac{1}{K}\sum_{k=1}^{K} \int \delta^2 P(z|\delta)P(\delta,j)d\delta = \\
&= \frac{1}{\sqrt{2\pi}K}\sum_{k=1}^{K} \int \delta^2 \frac{1}{\sqrt{2\pi}S_{kj}\sigma_0} e^{-\frac{(z-\delta)^2}{2\sigma_0^2} - \frac{\delta^2}{2S_{kj}^2}} d\delta = \\
&= \frac{1}{\sqrt{2\pi}K}\sum_{k=1}^{K} \frac{S_{kj}^2(\sigma_0^4 + \sigma_0^2 S_{kj}^2 + z^2 S_{kj}^2)}{(\sigma_0^2 + S_{kj}^2)^{\frac{5}{2}}} e^{-\frac{z^2}{2(\sigma_0^2 + S_{kj}^2)}},
\end{aligned} \tag{24}
$$

and

$$
\int_{z:\,|z|\geq z_t} C_2(z,N,j)dz = \frac{1}{K}\sum_{k=1}^{K}\Big[\frac{\sqrt{\frac{2}{\pi}}S_{kj}^4 z_t}{(\sigma_0^2+S_{kj}^2)^{\frac{3}{2}}} e^{-\frac{z_t^2}{2(\sigma_0^2+S_{kj}^2)}} + S_{kj}^2 \mathrm{erfc}\Big(\frac{z_t}{\sqrt{2(\sigma_0^2+S_{kj}^2)}}\Big)\Big], \tag{25}
$$

where $\text{erfc}(z) = \frac{2}{\sqrt{\pi}} \int_z^{\infty} e^{-t^2} dt$ is the complementary error function.

In addition one may be interested in $E(\delta^2|z) = C_2(z, N, j)/C_0(z, N, j)$ and $E(\delta|z) = C_1(z, N, j)/C_0(z, N, j)$, where

$$\begin{aligned}
C_1(z, N, j) &= \frac{1}{K} \sum_{k=1}^{K} \int \delta P(z|\delta) P(\delta, j) d\delta = \frac{1}{\sqrt{2\pi} K} \sum_{k=1}^{K} \frac{z S_{kj}^2}{(\sigma_0^2 + S_{kj}^2)^{\frac{3}{2}}} e^{-\frac{z^2}{2(\sigma_0^2 + S_{kj}^2)}}, \\
C_0(z, N, j) &= \frac{1}{K} \sum_{k=1}^{K} \int P(z|\delta) P(\delta, j) d\delta = \frac{1}{\sqrt{2\pi} K} \sum_{k=1}^{K} \frac{1}{(\sigma_0^2 + S_{kj}^2)^{\frac{1}{2}}} e^{-\frac{z^2}{2(\sigma_0^2 + S_{kj}^2)}},
\end{aligned} \tag{26}$$

Supplementary Figure 1a. Simulations: polygenic overlap estimates in bivariate analysis, without genetic correlation (simulated rho12=0)

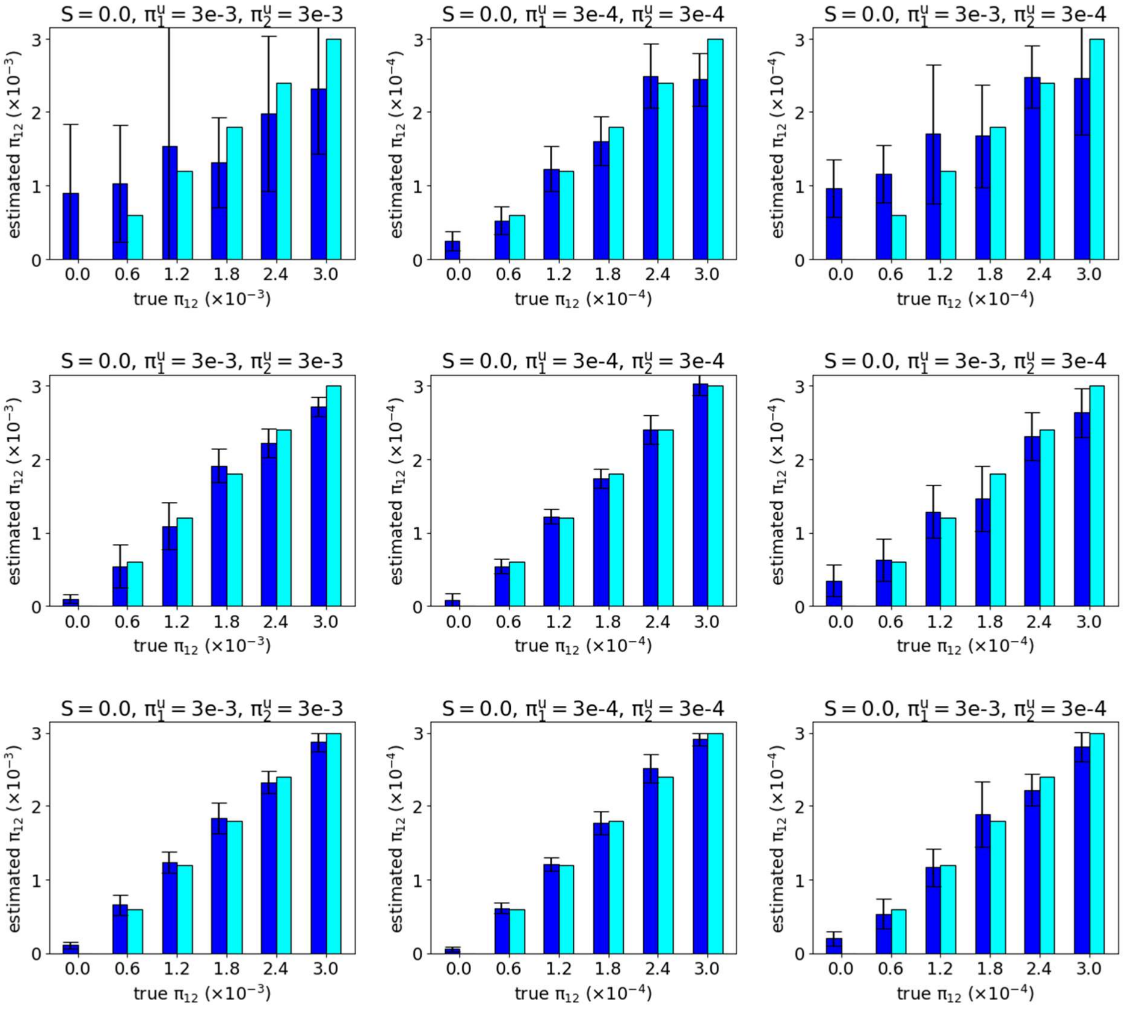

Simulation with synthetic GWAS summary statistics, generated according to MiXeR model, showing accuracy of π_{12} parameter in scenarios without genetic correlation (simulated rho12=0.0). The bars in blue indicate an average value of model estimates across 10 simulation runs. The bars in cyan show true (simulated) parameters. Error bars represent standard deviation of the model estimate across 10 simulation runs.

Supplementary Figure 1b. Simulations: polygenic overlap estimates in bivariate analysis, with genetic correlation (simulated rho12=0.5)

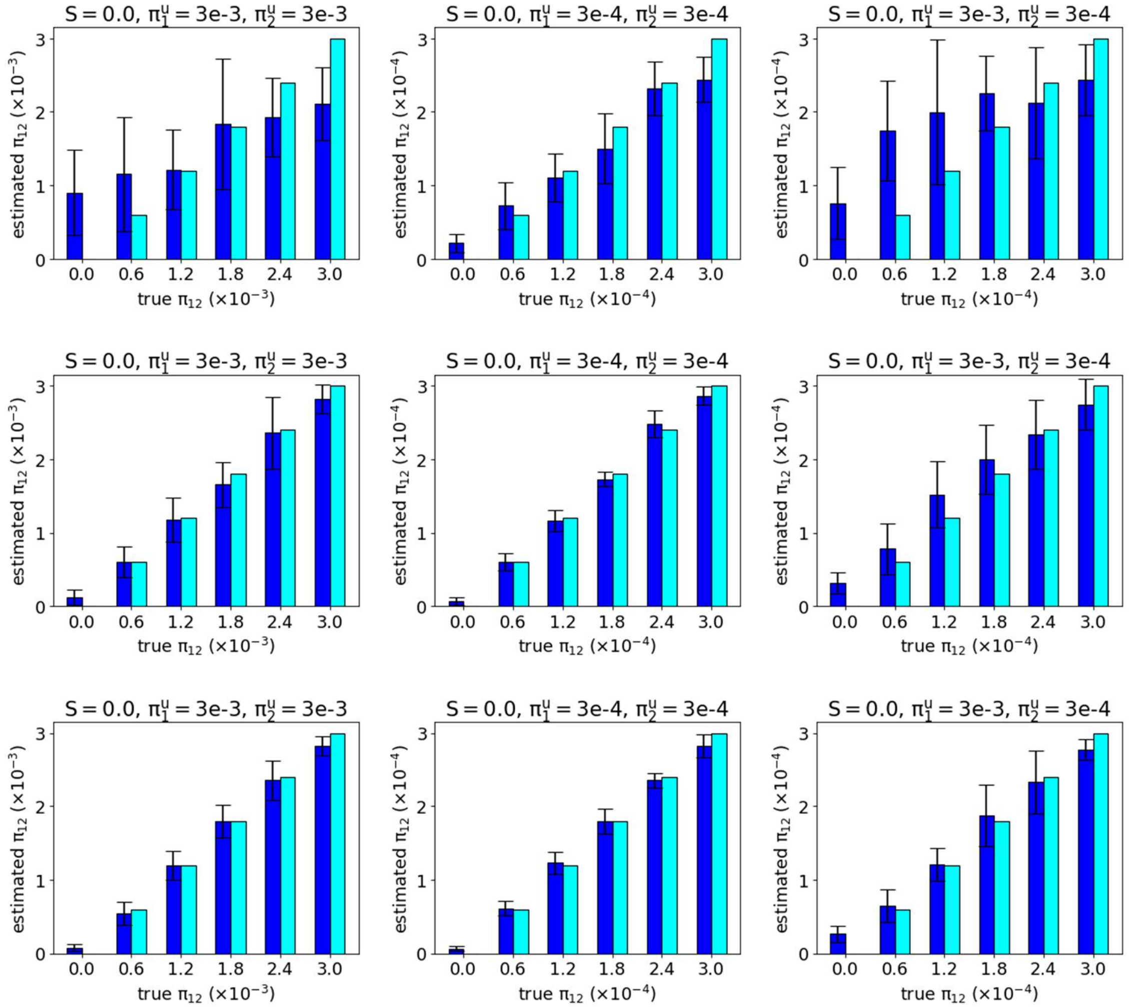

Simulation with synthetic GWAS summary statistics, generated according to MiXeR model, showing accuracy of π_{12} parameter in scenarios with genetic correlation (simulated rho12=0.5). Appearance of the data bars and error bars is the same as on the previous figure.

Supplementary Figure 2a. Simulations: estimates of the correlation of effect sizes in bivariate analysis, simulated rho12=0

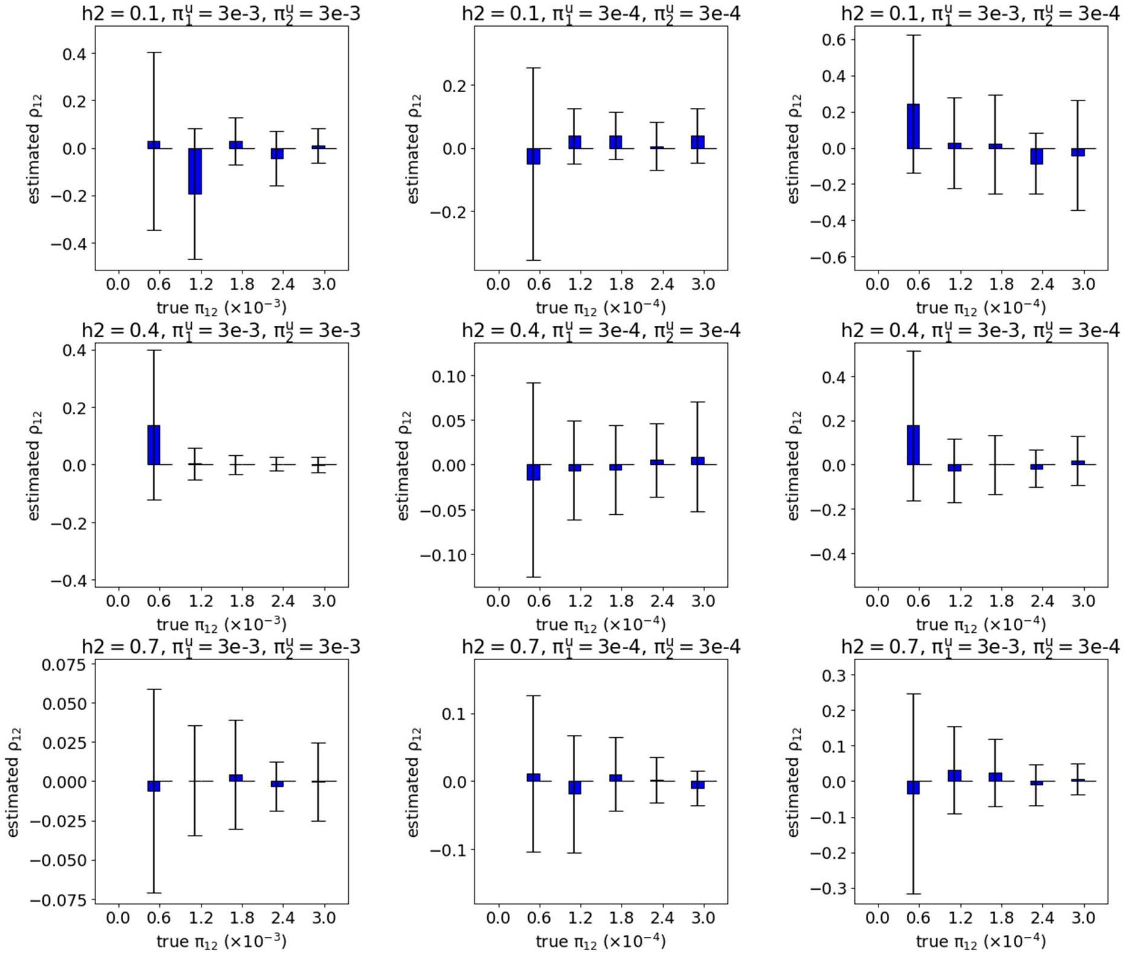

Simulation with synthetic GWAS summary statistics, generated according to MiXeR model, showing accuracy of rho12 parameter in scenarios without genetic correlation (simulated rho12=0). Appearance of the data bars and error bars is the same as on the previous figure.

Supplementary Figure 2b. Simulations: estimates of the correlation of effect sizes in bivariate analysis, simulated rho12=0.5

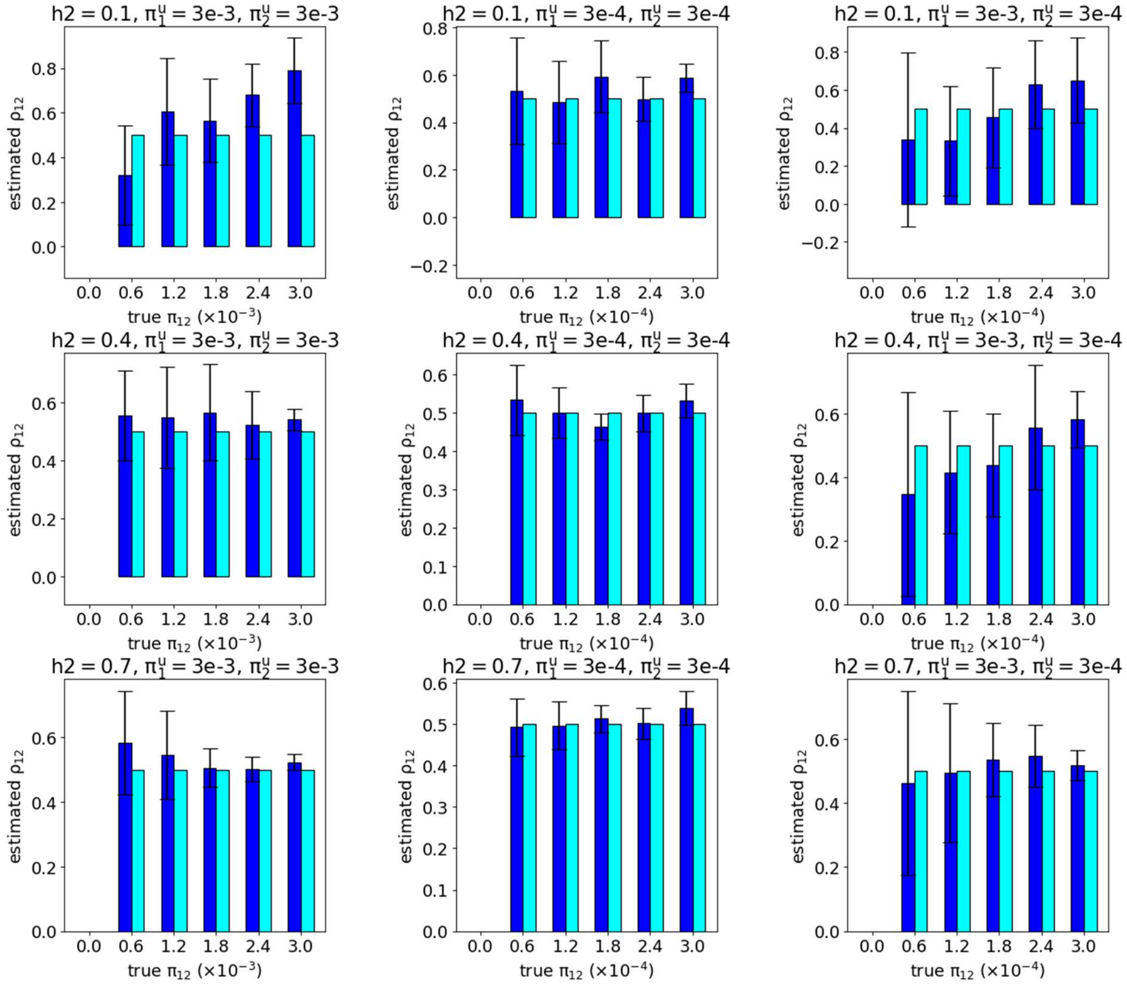

Simulation with synthetic GWAS summary statistics, generated according to MiXeR model, showing accuracy of rho12 parameter in scenarios with genetic correlation (simulated rho12=0.5). Appearance of the data bars and error bars is the same as on the previous figure.

Supplementary Figure 3a. Simulations: estimates of genetic correlation in bivariate analysis, simulated rho12=0

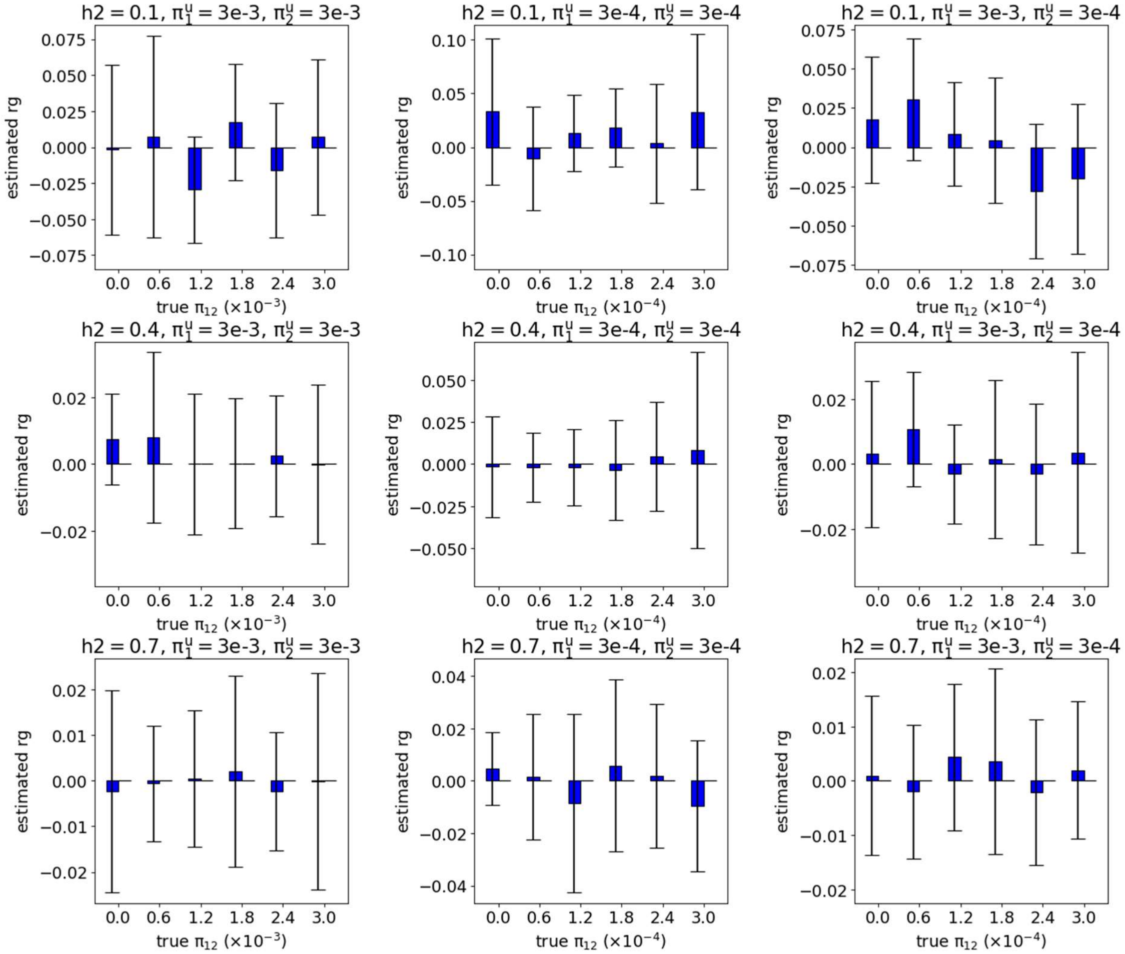

Simulation with synthetic GWAS summary statistics, generated according to MiXeR model, showing accuracy of $r_g = \rho_{12}\pi_{12}/\sqrt{\pi_1^u \pi_2^u}$ parameter in scenarios without genetic correlation (simulated rho12=0).

Supplementary Figure 3b. Simulations: estimates of genetic correlation in bivariate analysis, simulated rho12=0.5

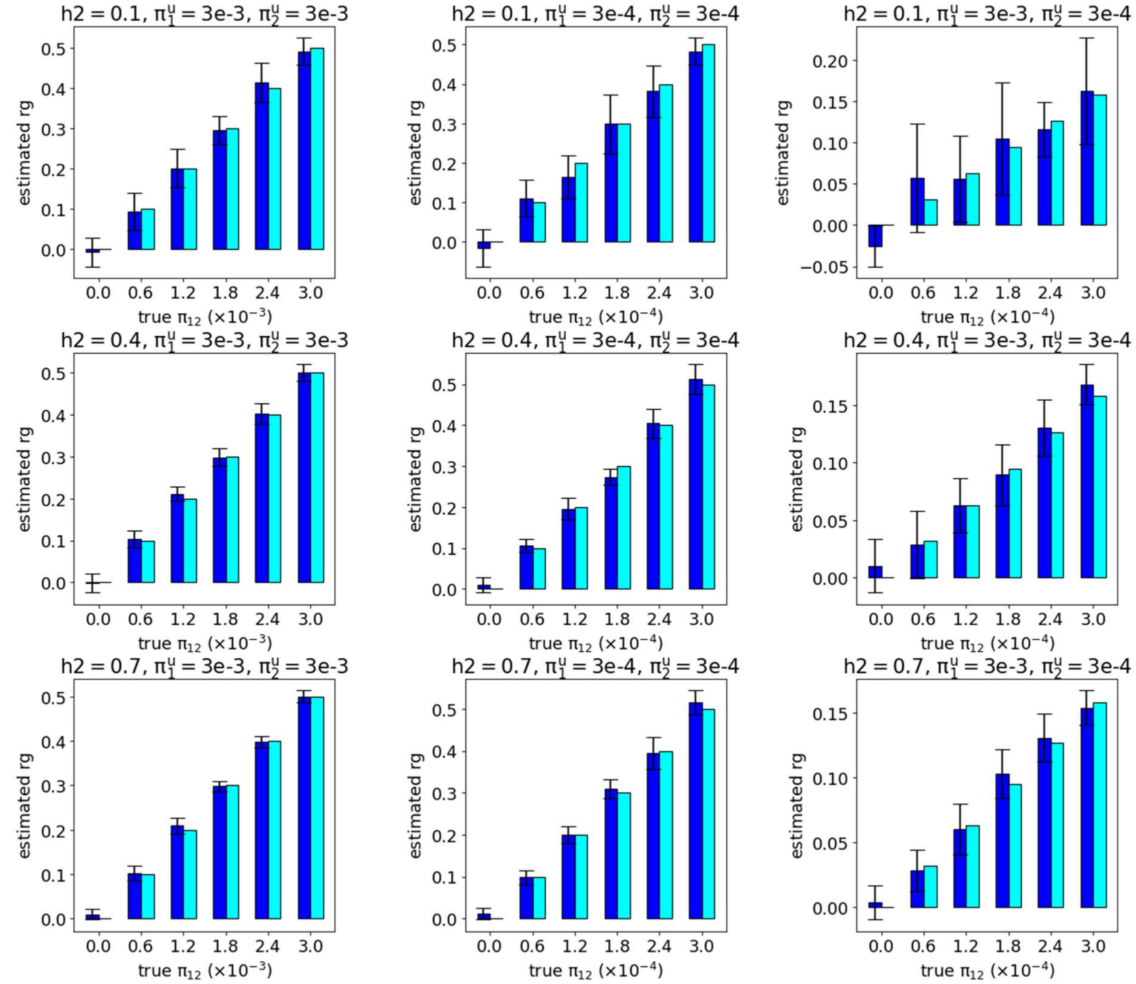

Simulation with synthetic GWAS summary statistics, generated according to MiXeR model, showing accuracy of $r_g = \rho_{12}\pi_{12}/\sqrt{\pi_1^u \pi_2^u}$ parameter in scenarios with genetic correlation (simulated rho12=0.5).

Supplementary Figure 4. Simulations: polygenicity and heritability estimates in univariate analysis

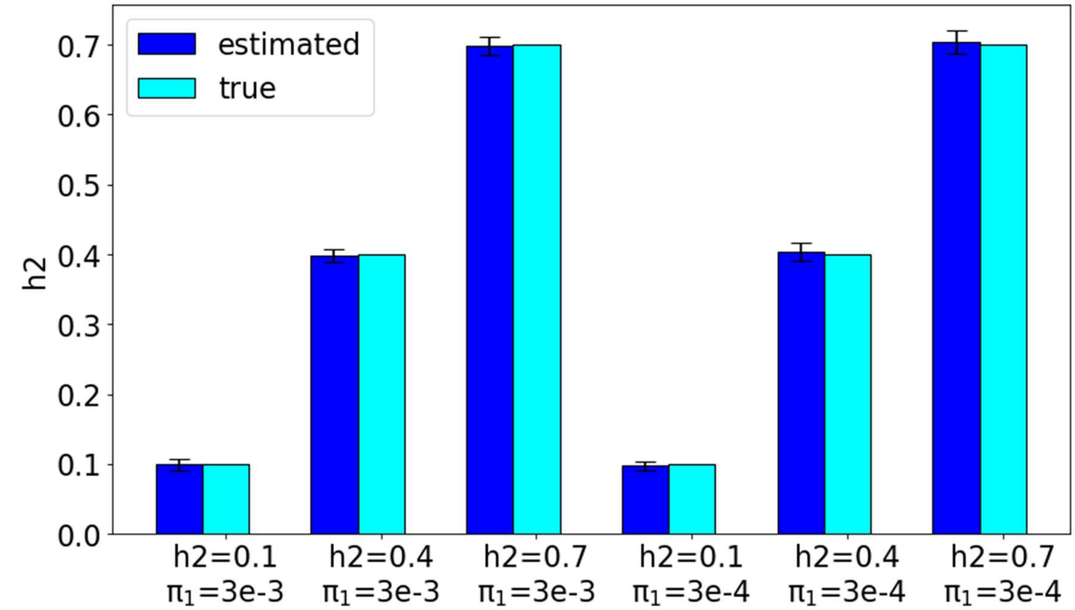

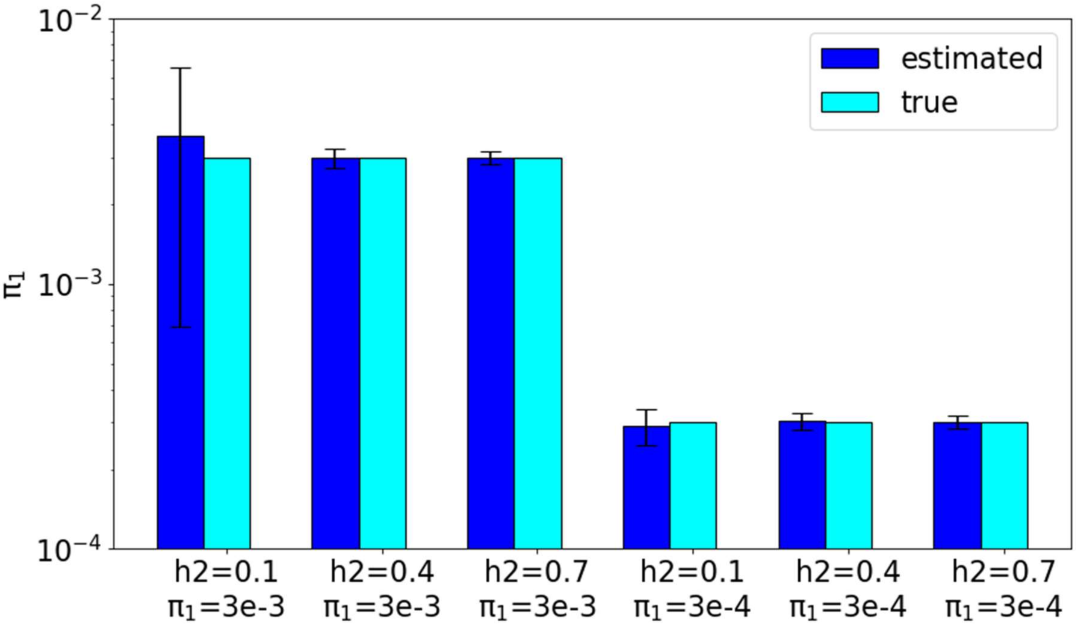

Simulations with univariate model. Top figure: validation of the heritability estimation. Bottom figure: validation of the polygenicity estimation. In total six scenarios are tested, with simulated heritability h2 set to 0.1, 0.4, 0.7 and polygenicity π_1 set to 3e-3, 3e-4. The bars in blue indicate an average value of model estimates across 120 simulation runs. The bars in cyan show true (simulated) parameters. Error bars represent standard deviation of the model estimate across 120 simulation runs.

Supplementary Figure 5. Simulations: Q-Q plots with simulated data

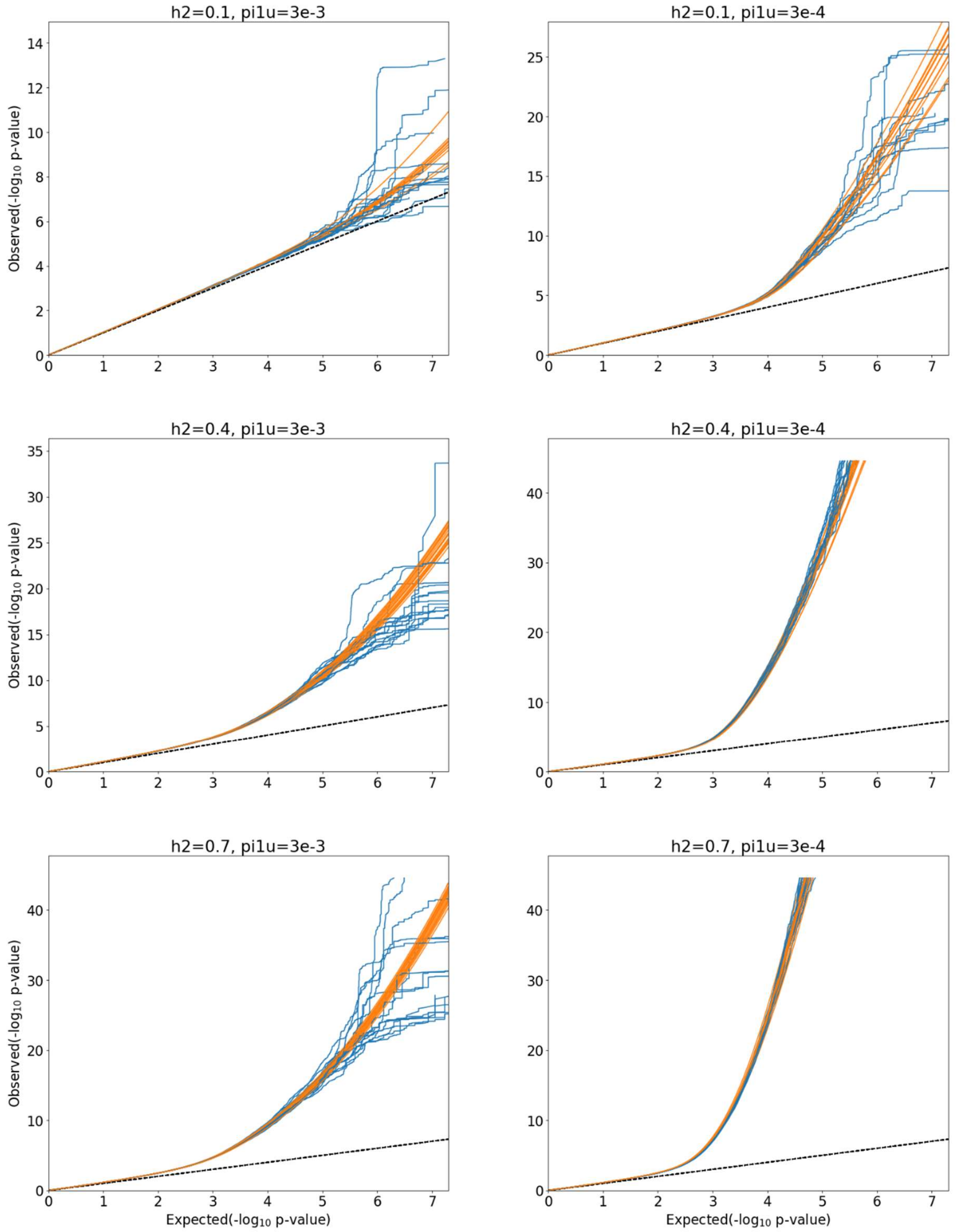

Simulated QQ plots and model prediction across six scenarios (two levels of polygenicity: 3e-3, 3e-4; three levels of heritability: 0.1, 0.4, 0.7). Each simulation was repeated 10 times with random instantiation of causal variants and their effects. Points on the QQ plot are weighted according to LD structure, using n=64 iterations of random pruning at LD threshold r2=0.1.

Supplementary Figure 6a. Simulations: Q-Q plots of SNPs partitioned into a grid of MAF and LD score, high polygenicity

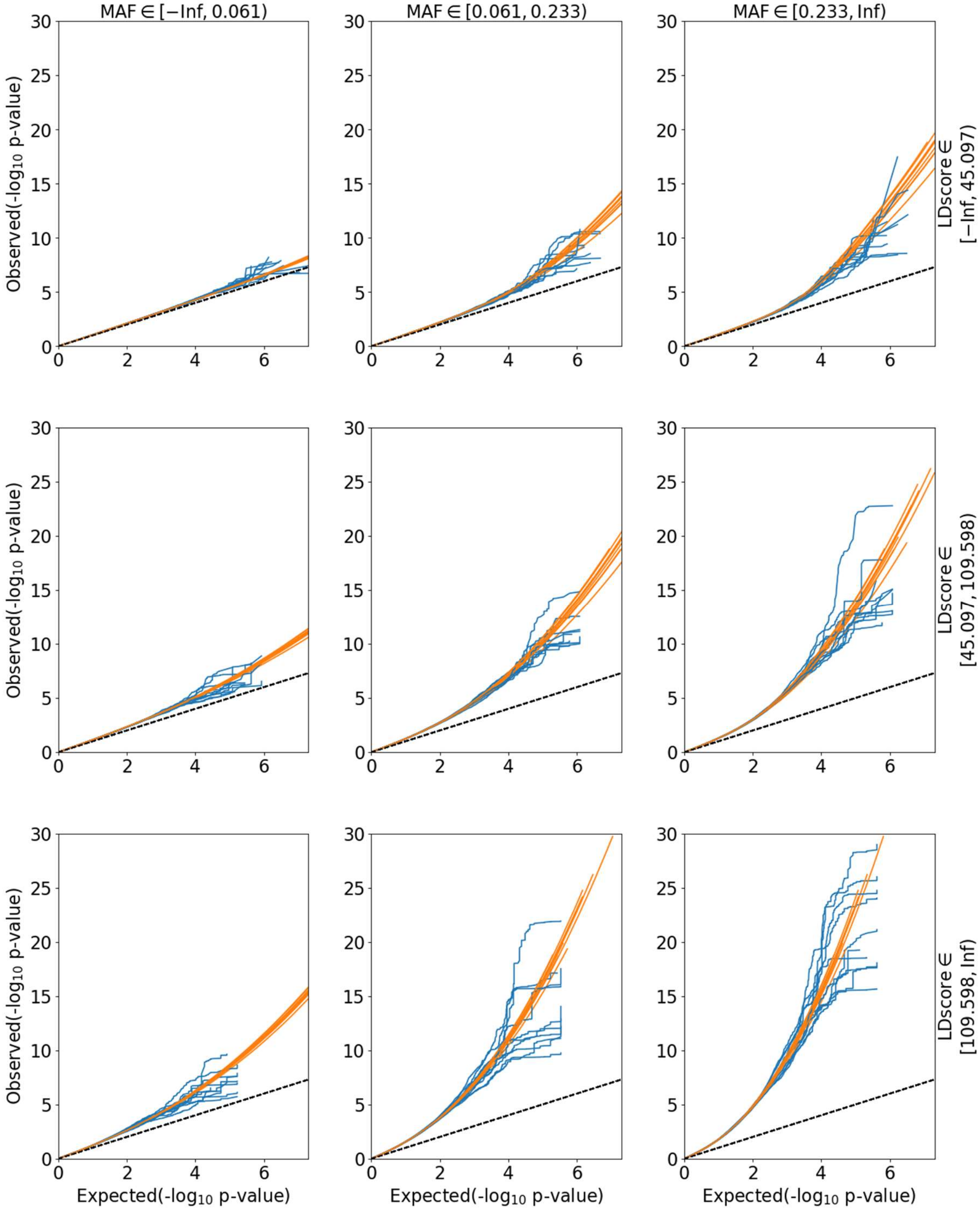

Simulated data. QQ plots for subsets of SNPs, partitioned into 9 groups according to minor allele frequency (MAF) and total LD score. Scenario with high polygenicity (pi1u=3e-03), heritability h2=0.4. Points on the QQ plot are weighted according to LD structure, using n=64 iterations of random pruning at LD threshold r2=0.1.

Supplementary Figure 6b. Simulations: Q-Q plots of SNPs partitioned into a grid of MAF and LD score, low polygenicity

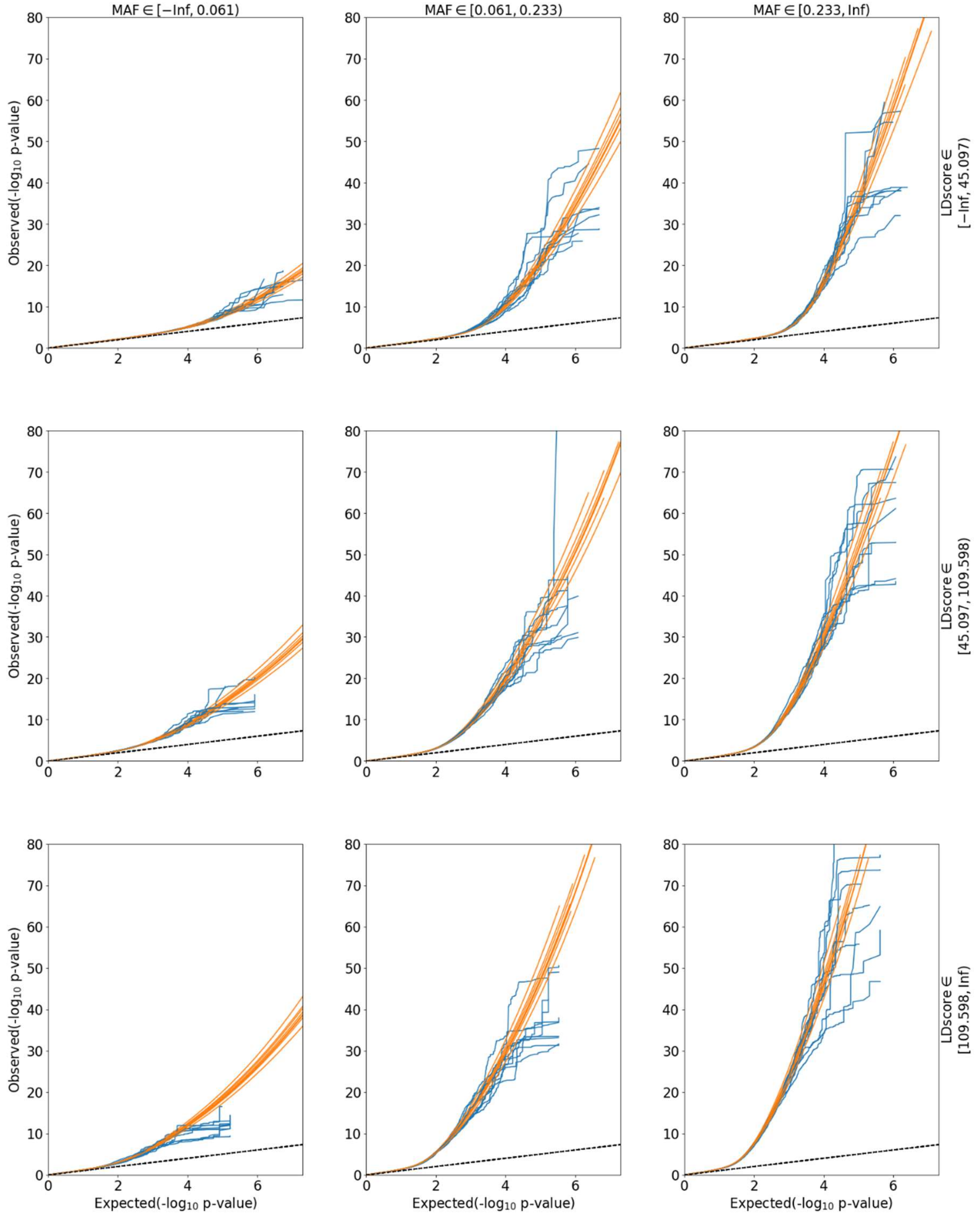

Simulated data. QQ plots for subsets of SNPs, partitioned into 9 groups according to minor allele frequency (MAF) and total LD score, showing scenario with low polygenicity (pi1u=3e-04), heritability h2=0.4. Points on the QQ plot are weighted according to LD structure, using n=64 iterations of random pruning at LD threshold r2=0.1.

Supplementary Figure 7. Simulations: stratified QQ plots for traits with and without polygenic overlap

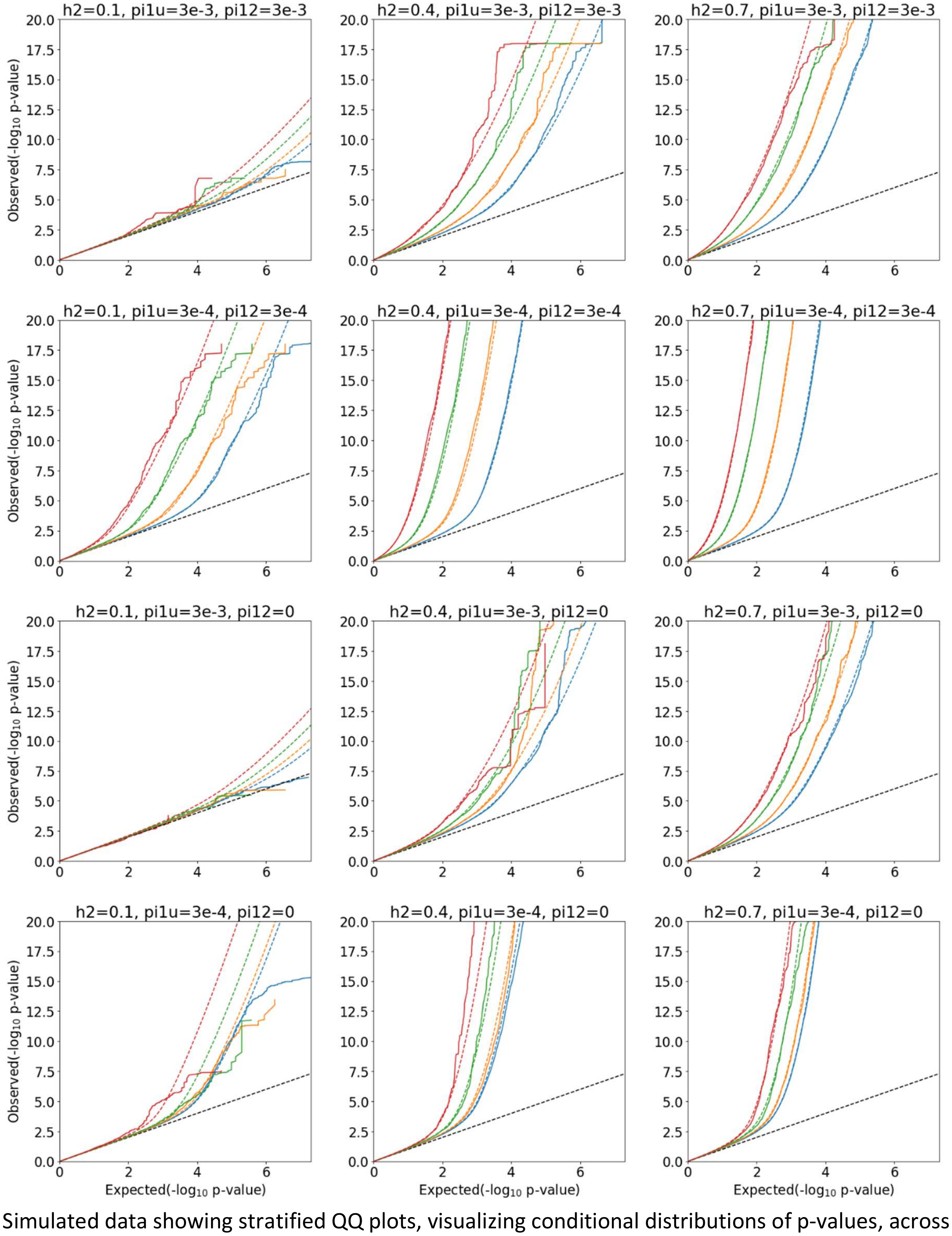

Simulated data showing stratified QQ plots, visualizing conditional distributions of p-values, across 12 scenarios (two levels of polygenicity: 3e-3, 3e-4; three levels of heritability: 0.1, 0.4, 0.7, with and without polygenic overlap). Top six figures represent scenarios with polygenic overlap at causal level. Bottom six figures are showing scenarios without polygenic overlap. Points on the QQ plot are weighted according to LD structure, using n=64 iterations of random pruning at LD threshold r2=0.1.

Supplementary Figure 8a. Sensitivity analysis: differential enrichment profile of synthesized data, simulated with MAF architecture following MiXeR assumptions

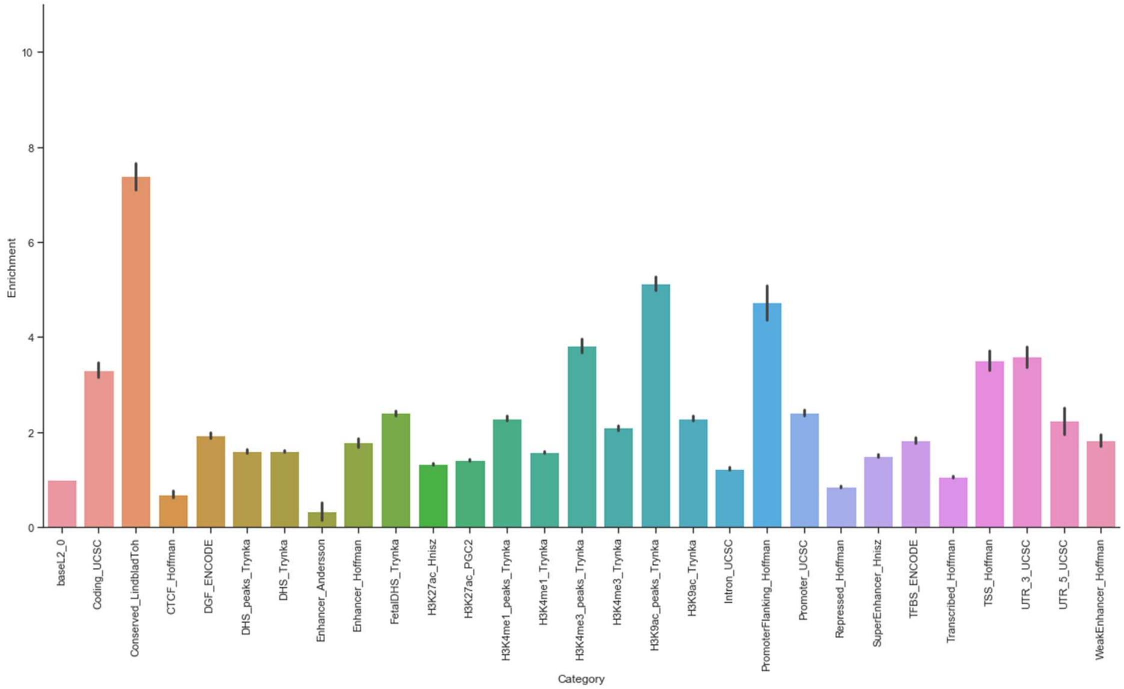

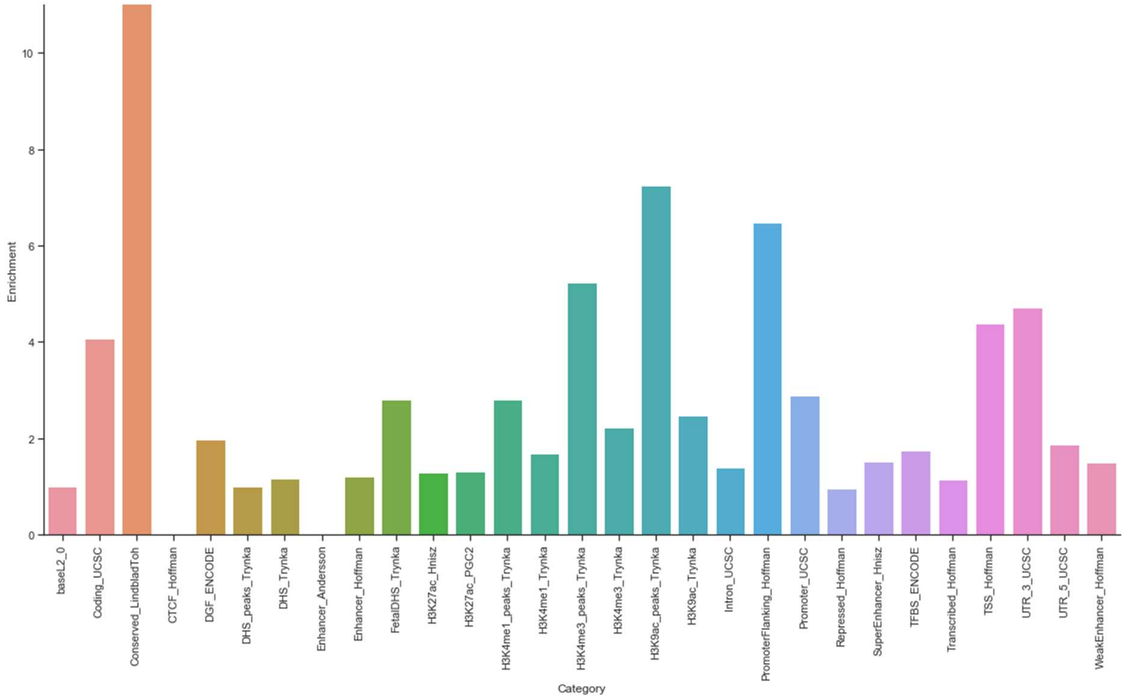

Comparison of enrichment between simulated data (top) and enrichment of Schizophrenia (bottom), estimated with stratified LDSR using `--per-allele` option for LD score estimation

Supplementary Figure 8b. Sensitivity analysis: differential enrichment profile of synthesized data, simulated with MAF architecture following LDSR assumptions

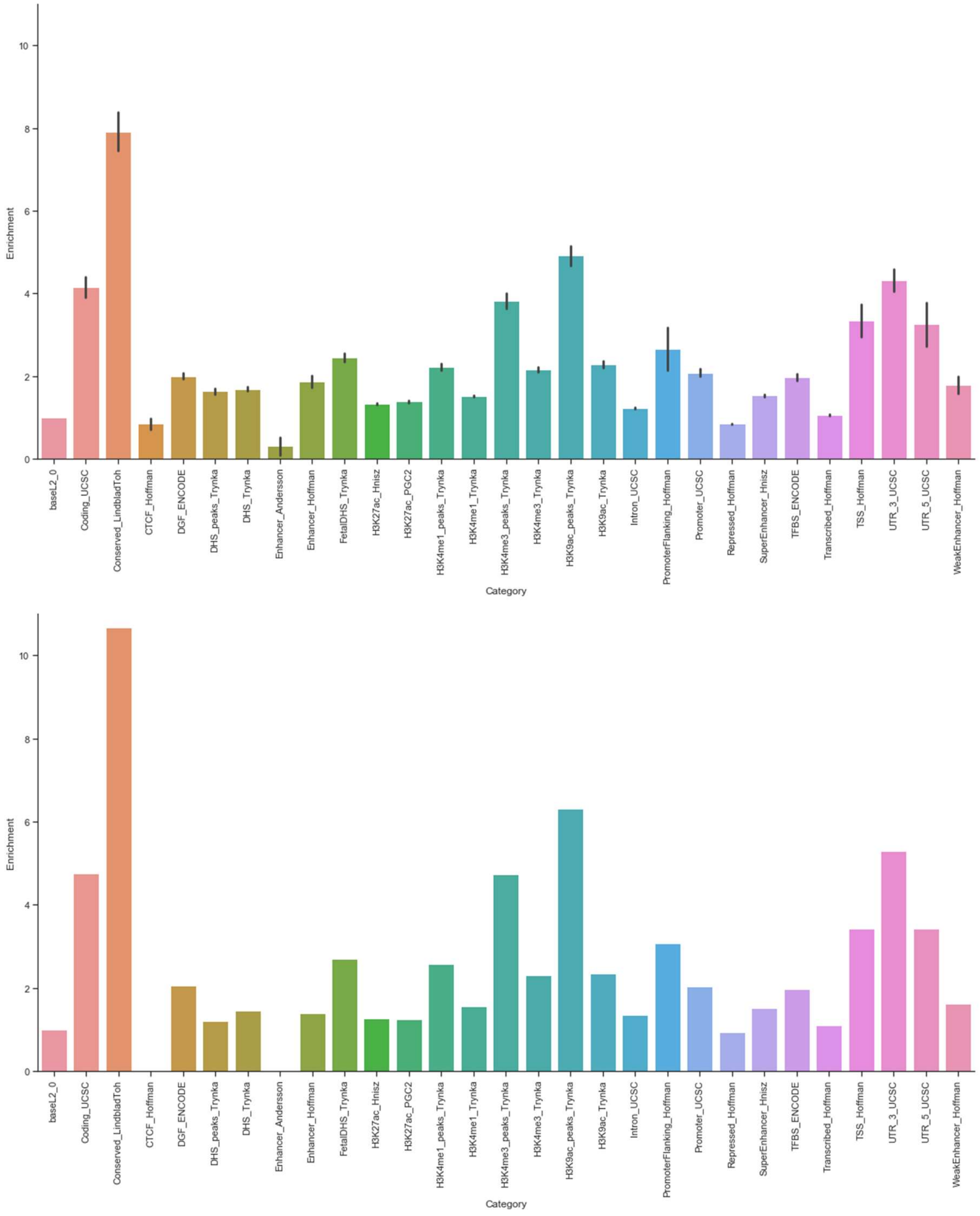

Comparison of enrichment between simulated data (top) and enrichment of Schizophrenia (bottom), estimated with stratified LDSR.

Supplementary Figure 9. Sensitivity analysis: polygenicity and heritability estimates under differential genomic enrichment, with MAF architecture simulated following MiXeR assumptions

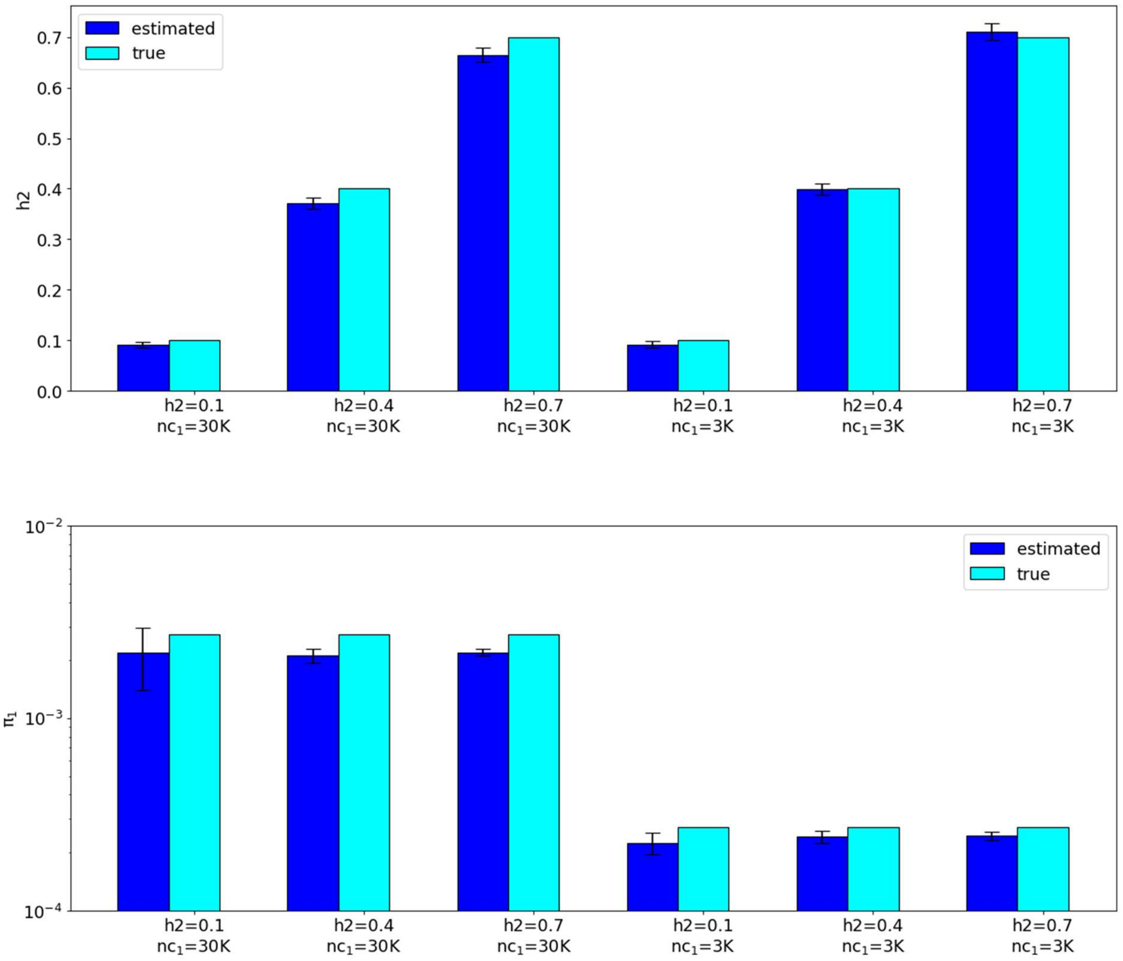

Simulations with model misspecification showing a minor bias in univariate estimates in the presence of genomic annotations. Top figure: validation of the heritability estimation. Bottom figure: validation of the polygenicity estimation. The results of bivariate analysis are presented in a separate table. Appearance of the data bars and error bars is the same as on Supplementary Figure 4.

Supplementary Figure 10a. Sensitivity analysis for misspecified MAF-dependent architecture: univariate MiXeR estimates of heritability and polygenicity

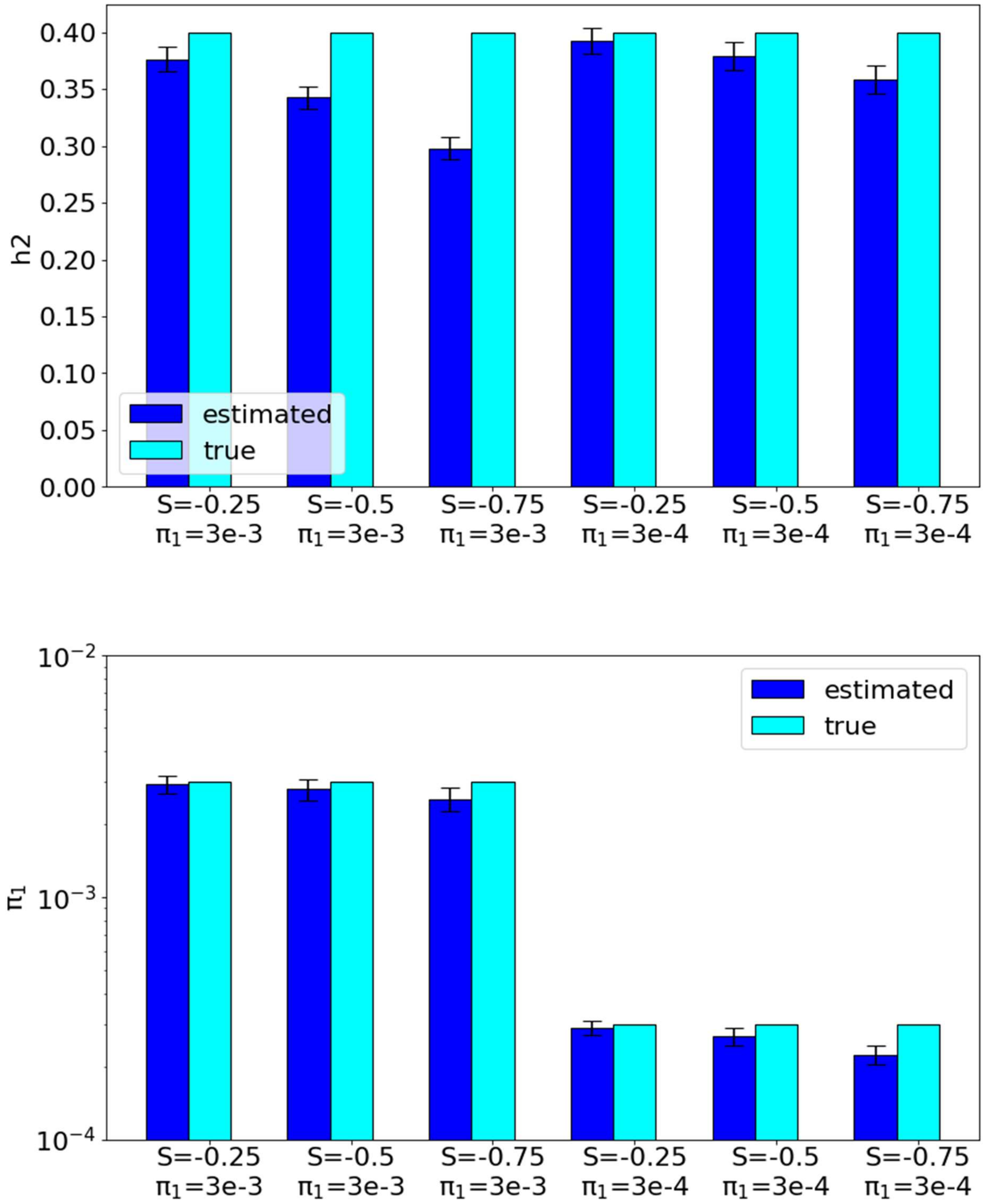

Simulations with model misspecification showing bias in univariate estimates for misspecified MAF-dependent architecture. Top figure: validation of the heritability estimation. Bottom figure: validation of the polygenicity estimation. "S" parameter describes simulated MAF-dependent architecture (with S=-1 corresponding to LDSR MAF model, S=0 corresponding to MiXeR MAF model). Simulated heritability is 0.4. Appearance of the data bars and error bars is the same as on Supplementary Figure 4.

Supplementary Figure 10b. Sensitivity analysis for misspecified MAF-dependent architecture: polygenic overlap estimates in bivariate analysis, simulated rho12=0.

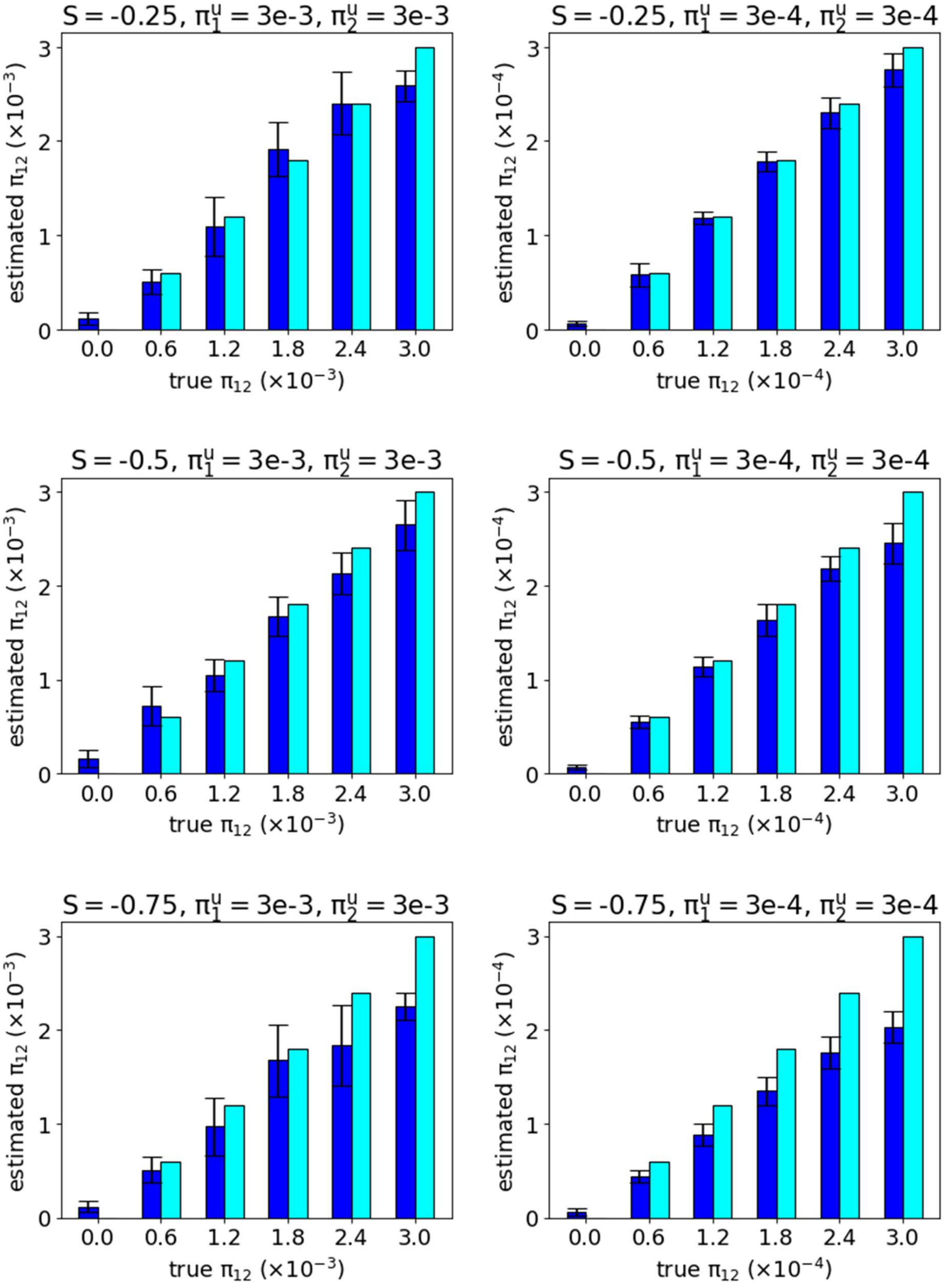

Simulation with synthetic GWAS summary statistics, showing accuracy of π_{12} estimates. "S" parameter describes simulated MAF-dependent architecture. Appearance of the data bars and error bars is the same as in Supplementary Figure 1. Simulated heritability is 0.4, simulated $\rho_{12} = 0$.

Supplementary Figure 10c. Sensitivity analysis for misspecified MAF-dependent architecture: polygenic overlap estimates in bivariate analysis, simulated rho12=0.5

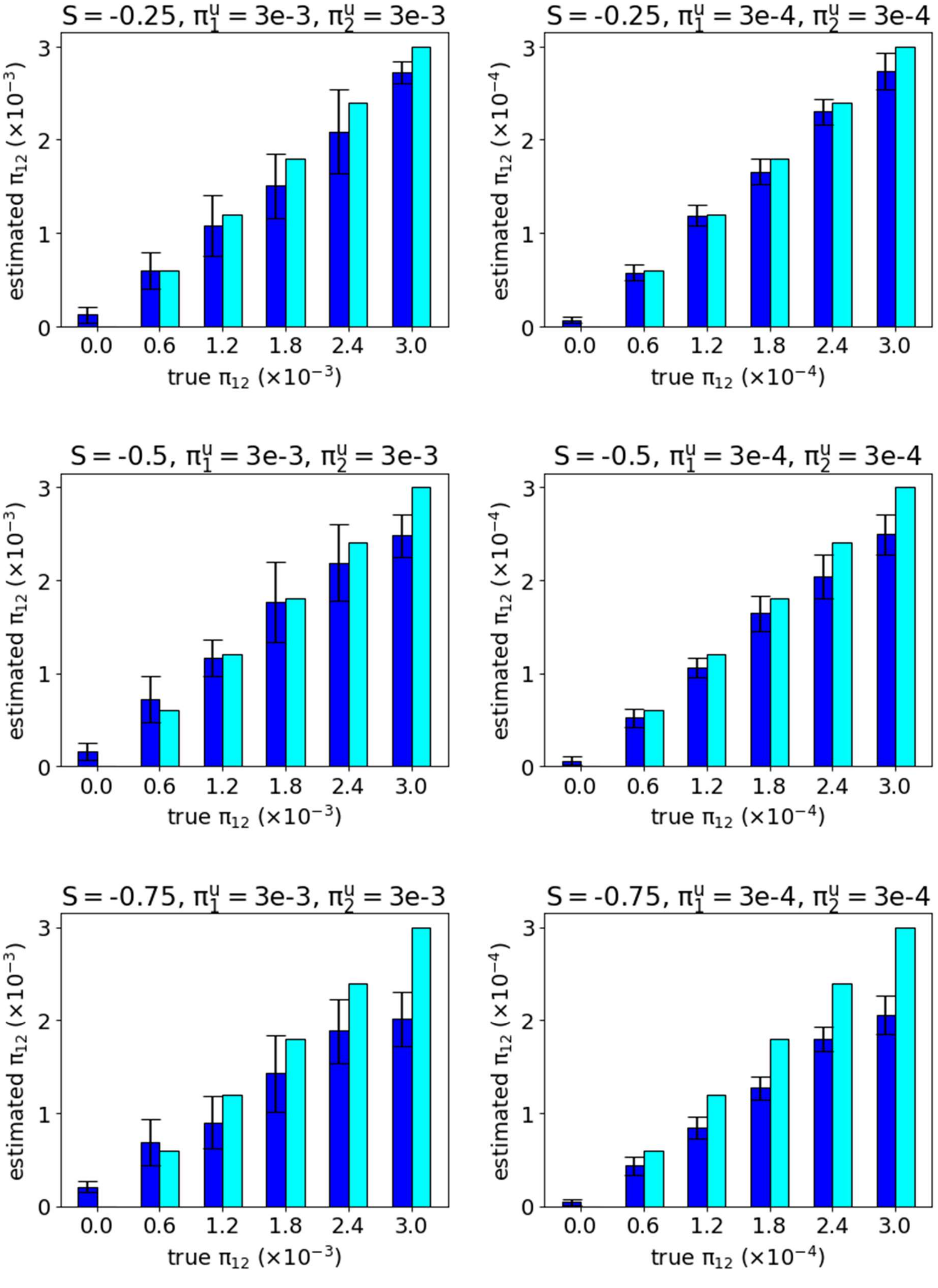

Simulation with synthetic GWAS summary statistics, showing accuracy of π_{12} estimates. "S" parameter describes simulated MAF-dependent architecture. Appearance of the data bars and error bars is the same as in Supplementary Figure 1. Simulated heritability is 0.4, simulated $\rho_{12} = 0.5$.

Supplementary Figure 10d. Sensitivity analysis for misspecified MAF-dependent architecture: estimates of the correlation of effect sizes in bivariate analysis, simulated rho12=0

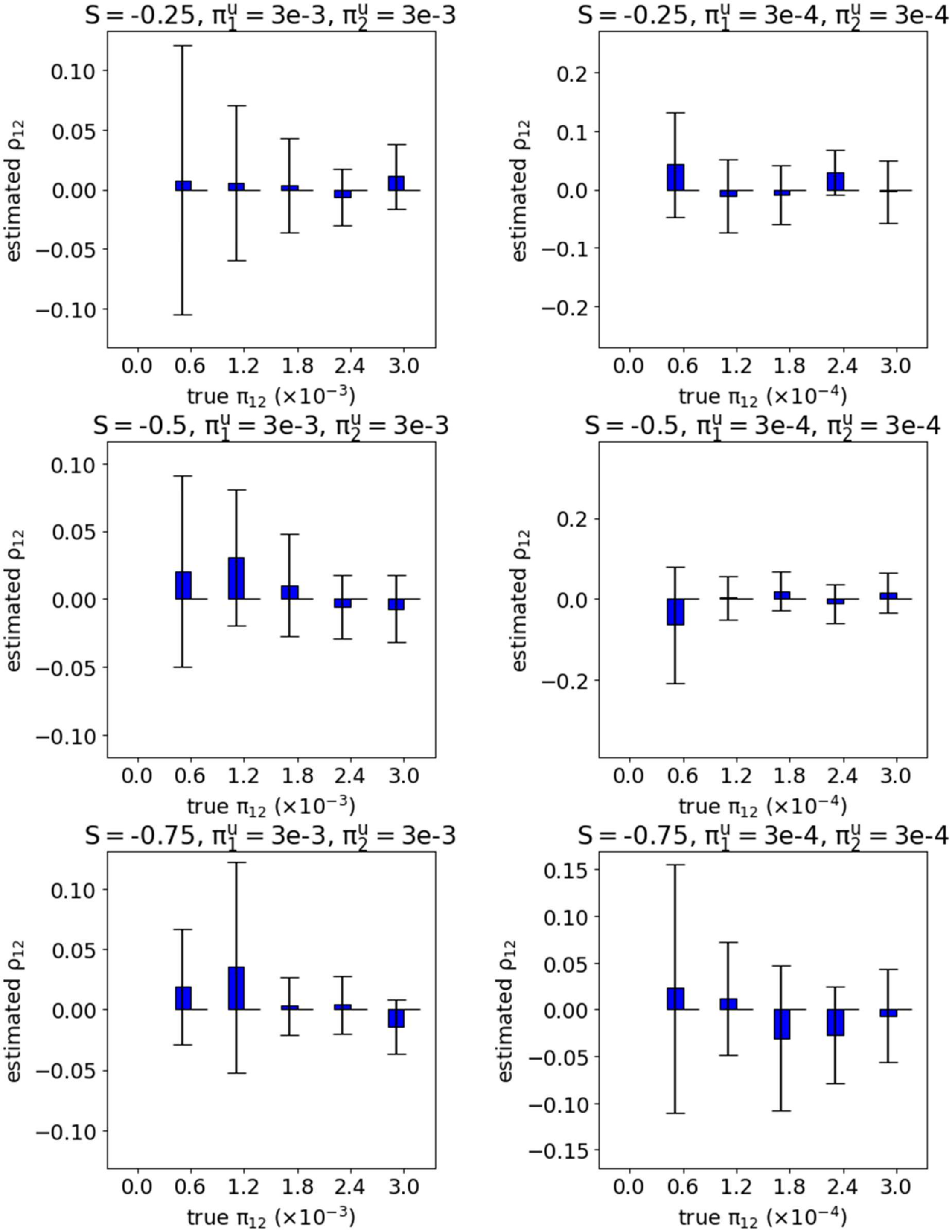

Simulation with synthetic GWAS summary statistics, showing accuracy of ρ_{12} estimates. "S" parameter describes simulated MAF-dependent architecture. Appearance of the data bars and error bars is the same as in Supplementary Figure 1. Simulated heritability is 0.4, simulated $\rho_{12} = 0$.

Supplementary Figure 10e. Sensitivity analysis for misspecified MAF-dependent architecture: estimates of the correlation of effect sizes in bivariate analysis, simulated rho12=0.5

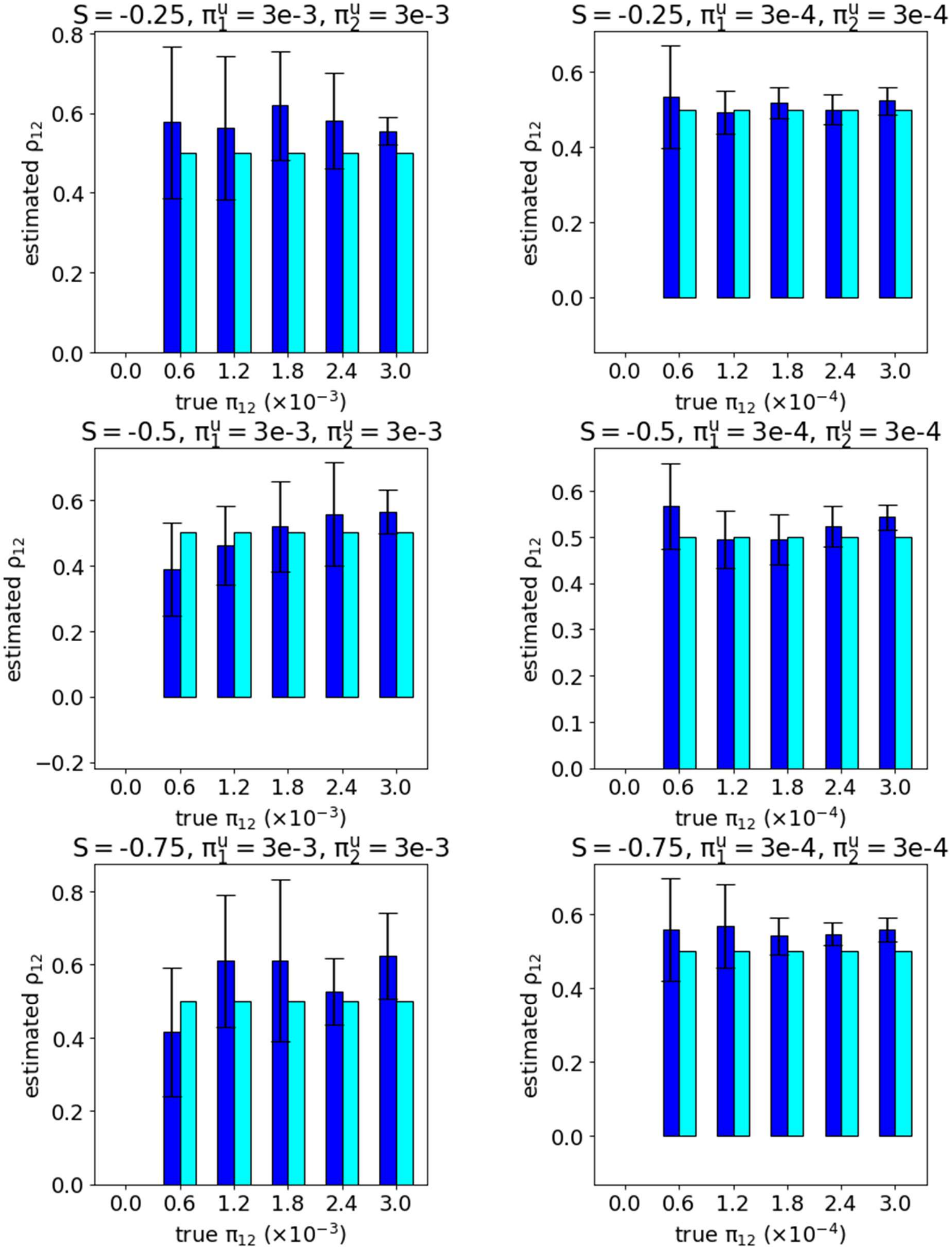

Simulation with synthetic GWAS summary statistics, showing accuracy of ρ_{12} estimates. "S" parameter describes simulated MAF-dependent architecture. Appearance of the data bars and error bars is the same as in Supplementary Figure 1. Simulated heritability is 0.4, simulated $\rho_{12} = 0.5$.

Supplementary Figure 10f. Sensitivity analysis for misspecified MAF-dependent architecture: estimates of genetic correlation in bivariate analysis, simulated rho12=0

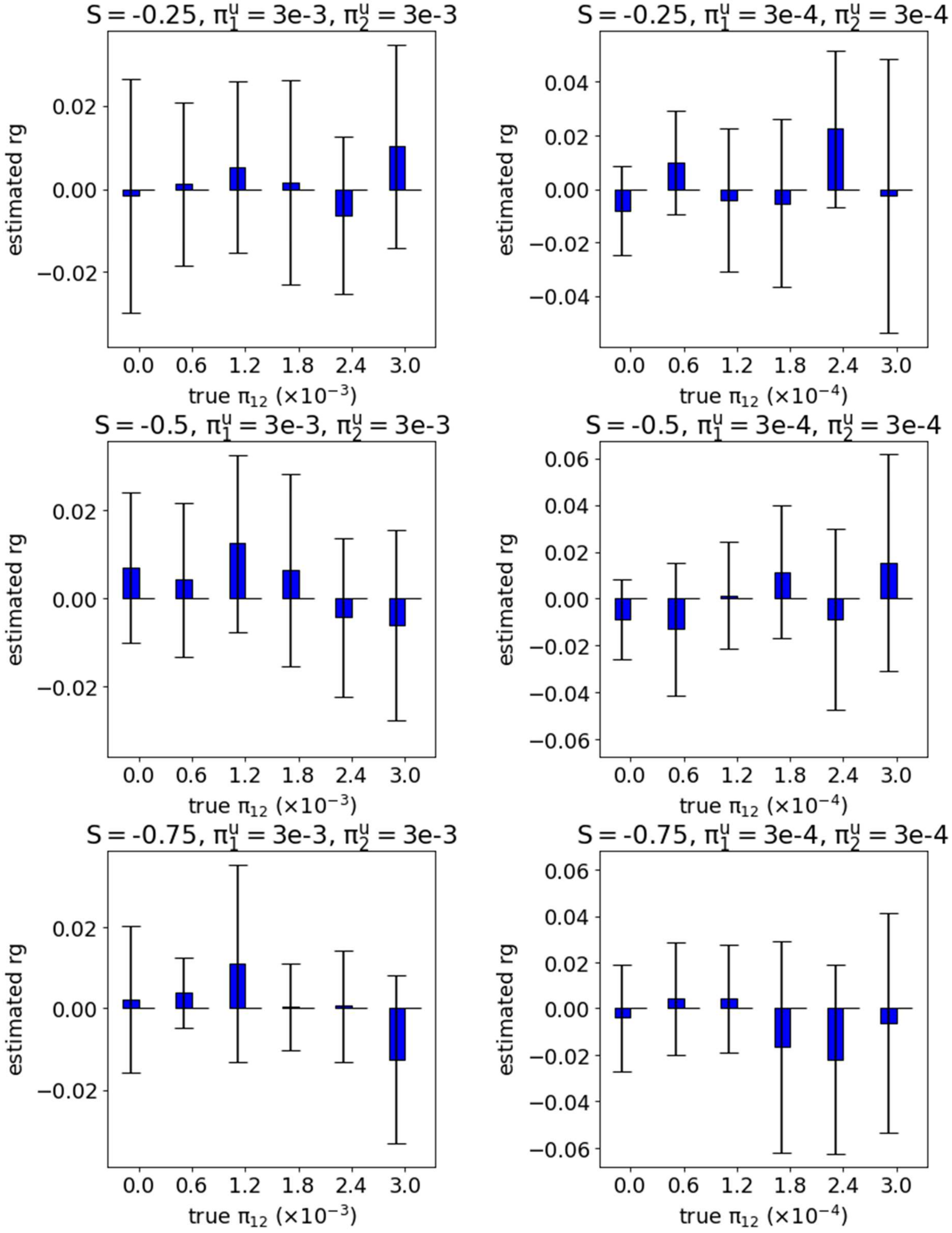

Simulation with synthetic GWAS summary statistics, showing accuracy of $r_g = \rho_{12}\pi_{12}/\sqrt{\pi_1^u \pi_2^u}$ estimates. "S" parameter describes simulated MAF-dependent architecture. Appearance of the data bars and error bars is the same as in Supplementary Figure 1. Simulated heritability is 0.4, simulated $\rho_{12} = 0$.

Supplementary Figure 10g. Sensitivity analysis for misspecified MAF-dependent architecture: estimates of genetic correlation, simulated rho12=0.5

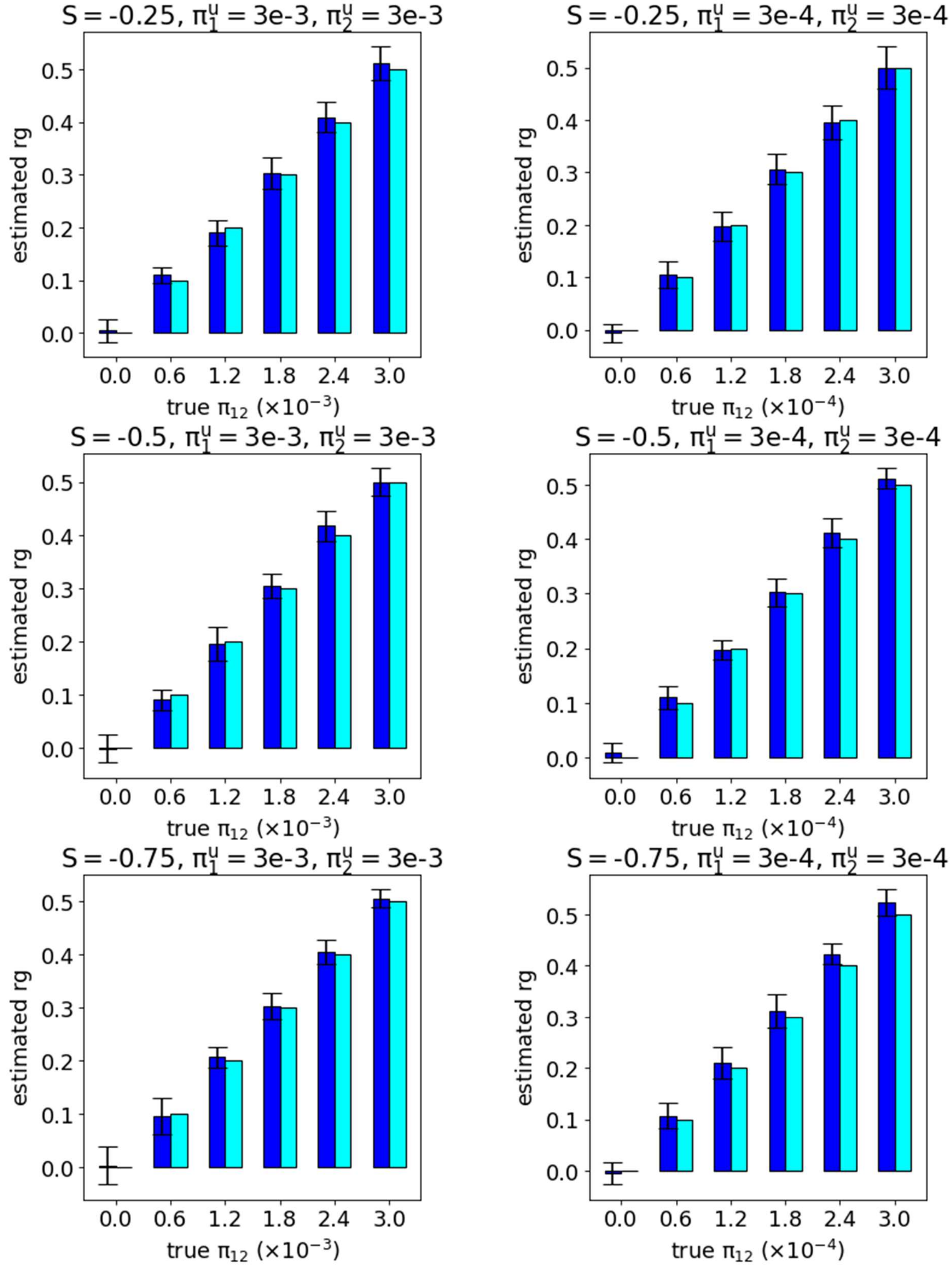

Simulation with synthetic GWAS summary statistics, showing accuracy of $r_g = \rho_{12}\pi_{12}/\sqrt{\pi_1^u \pi_2^u}$ estimates. "S" parameter describes simulated MAF-dependent architecture. Appearance of the data bars and error bars is the same as in Supplementary Figure 1. Simulated heritability is 0.4, simulated $\rho_{12} = 0.5$.

Supplementary Figure 11. Cumulated fraction of explained heritability versus proportion of causal variants

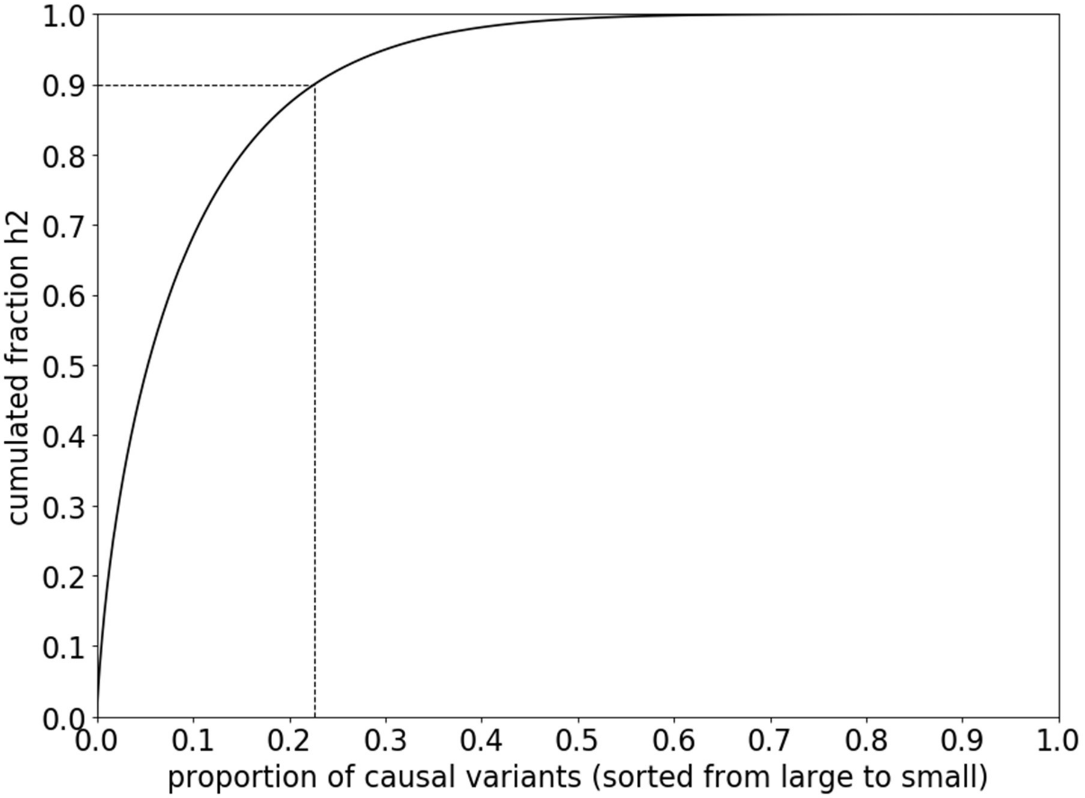

In MiXeR model the contribution of causal variant to heritability depends on the effect of the variant, and on its minor allele frequency: $h_2 = \beta_j^2 \times 2p_j(1 - p_j)$. Since many SNPs have low allele frequency, only a small fraction (22.6%) of causal variants with relatively large effects explains 90% of the total heritability.

Supplementary Figure 12. Venn diagrams across all traits

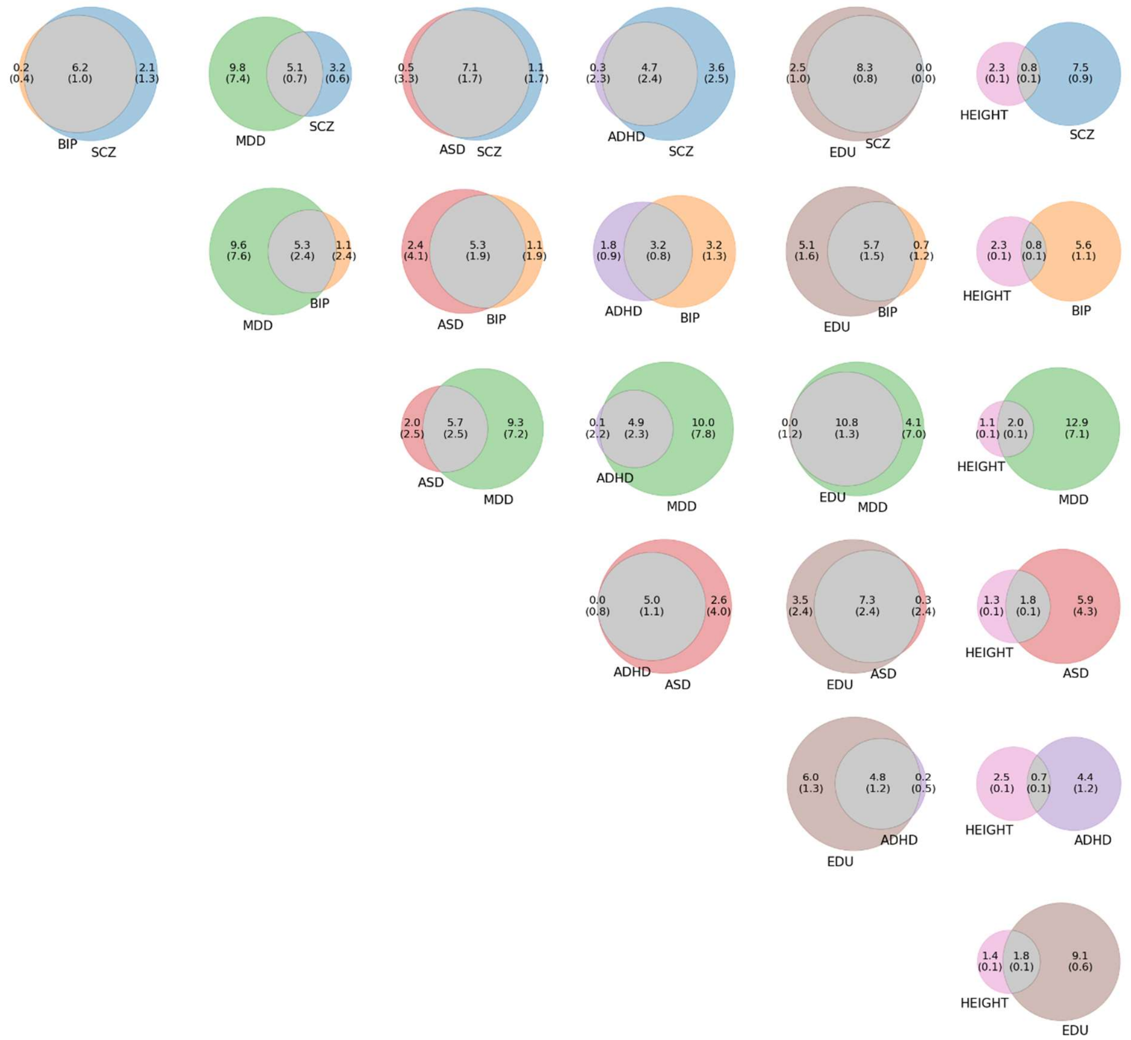

Venn diagrams of unique and shared polygenic component at the causal level. SCZ: schizophrenia; BIP: bipolar disorder; MDD: major depressive disorder; ASD: autism spectrum disorder; ADHD: attention deficit / hyperactivity disorder; EDU: educational attainment. The numbers indicate estimated quantity of causal variants (in 1,000) per component, explaining 90% of SNP heritability in each phenotype, followed by the standard error. The size of circles reflects the polygenicity (scaled individually for each of the Venn diagrams).

Supplementary Figure 13a. Venn diagrams and conditional cross-trait QQ plots for schizophrenia

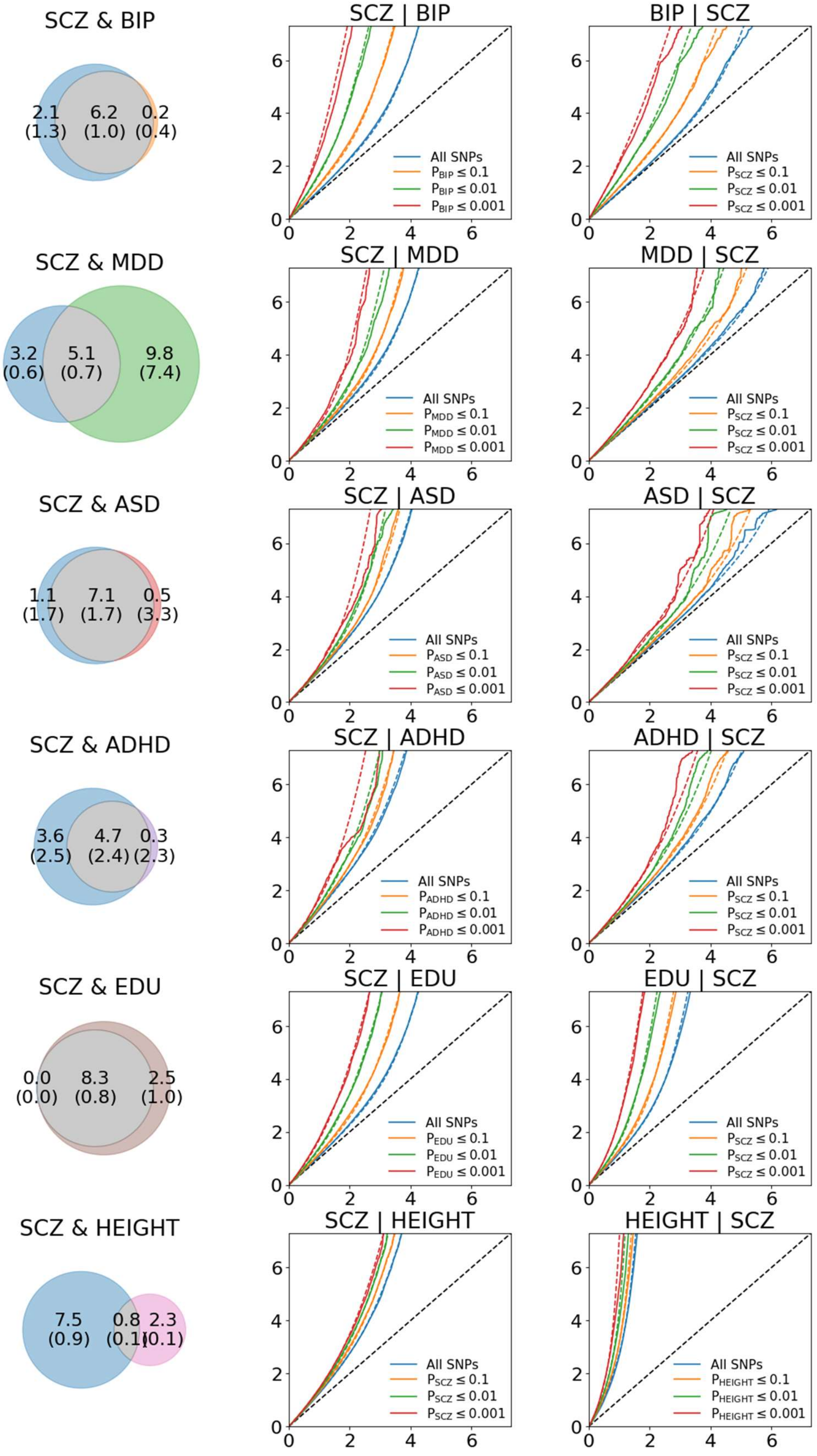

Venn Diagrams and stratified QQ plots, visualizing conditional distribution of p-values.

Supplementary Figure 13b. Venn diagrams and conditional cross-trait QQ plots for bipolar disorder

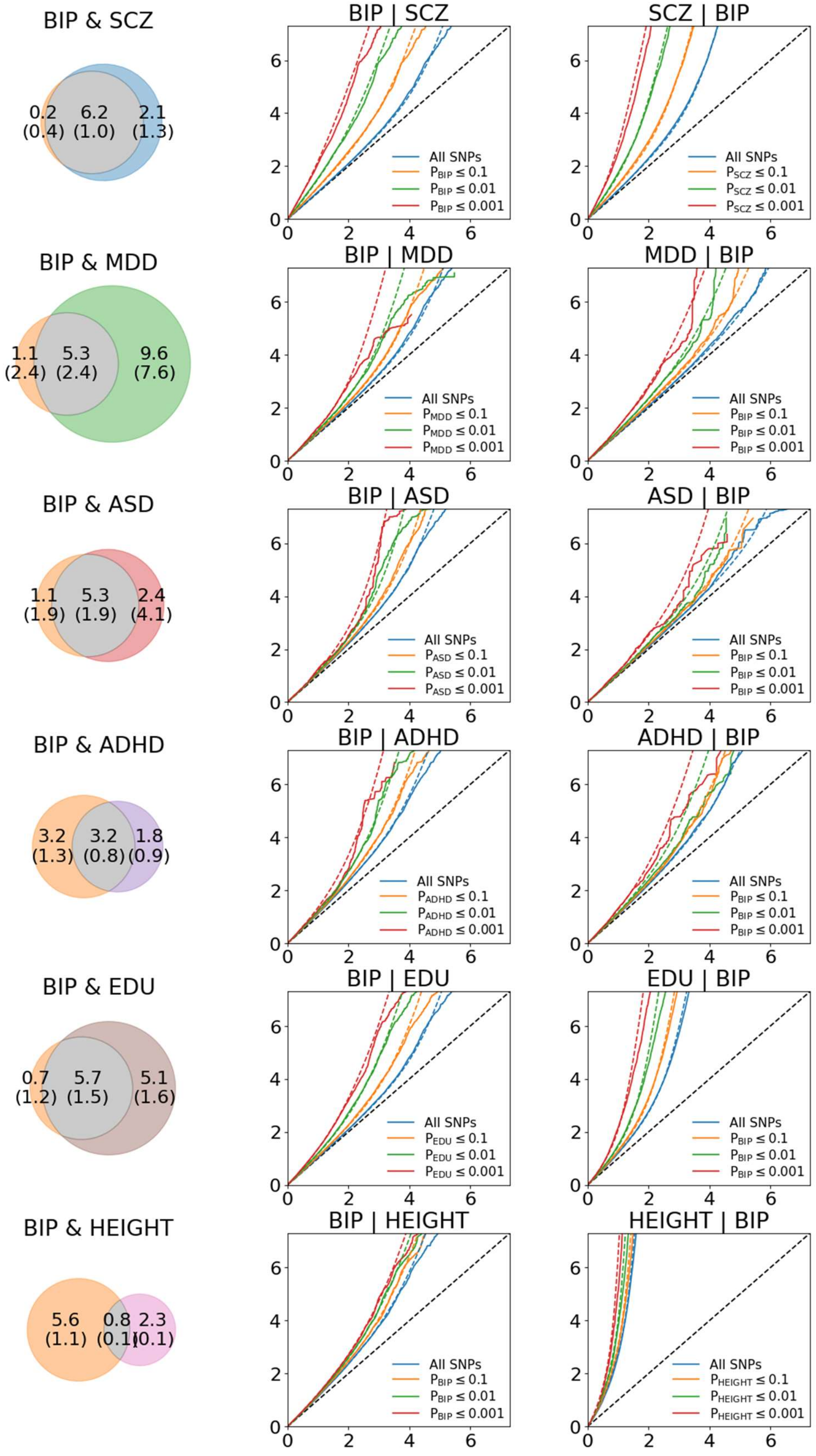

Venn Diagrams and stratified QQ plots, visualizing conditional distribution of p-values.

Supplementary Figure 13c. Venn diagrams and conditional cross-trait QQ plots for major depressive disorder

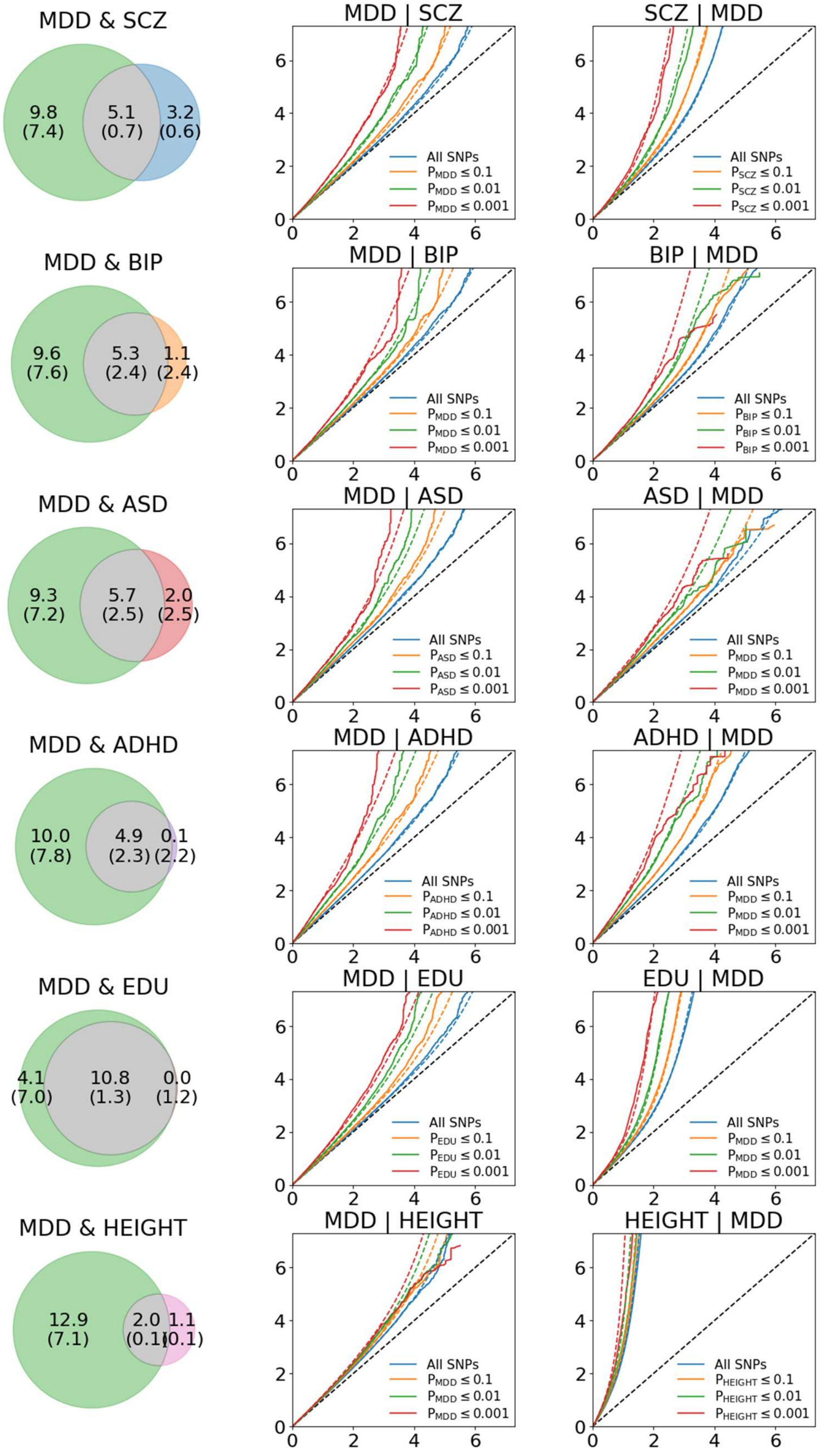

Venn Diagrams and stratified QQ plots, visualizing conditional distribution of p-values.

Supplementary Figure 13d. Venn diagrams and conditional cross-trait QQ plots for autism spectrum disorder

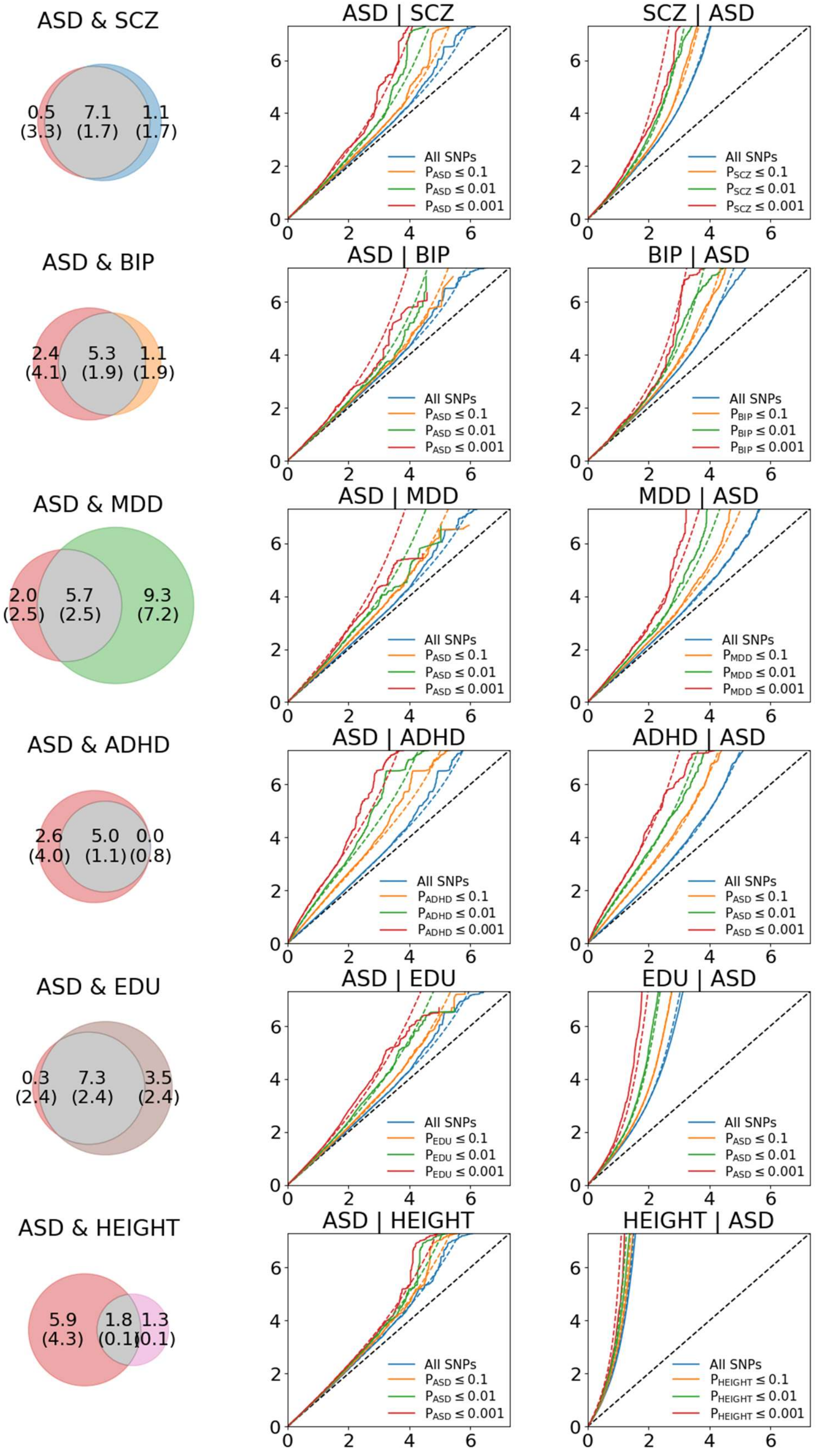

Venn Diagrams and stratified QQ plots, visualizing conditional distribution of p-values.

Supplementary Figure 13e. Venn diagrams and conditional cross-trait QQ plots for ADHD

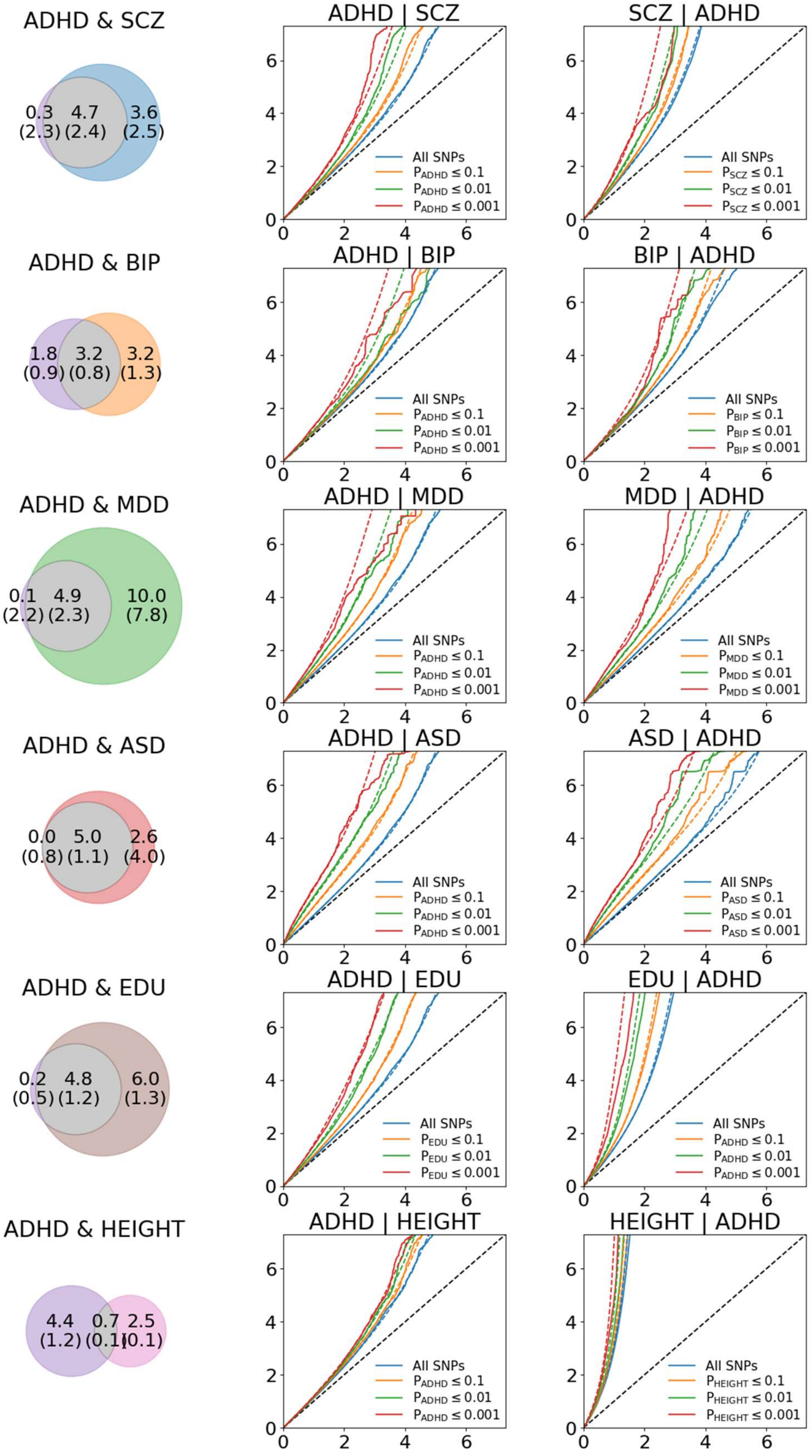

Venn Diagrams and stratified QQ plots, visualizing conditional distribution of p-values.

Supplementary Figure 13f. Venn diagrams and conditional cross-trait QQ plots for educational attainment

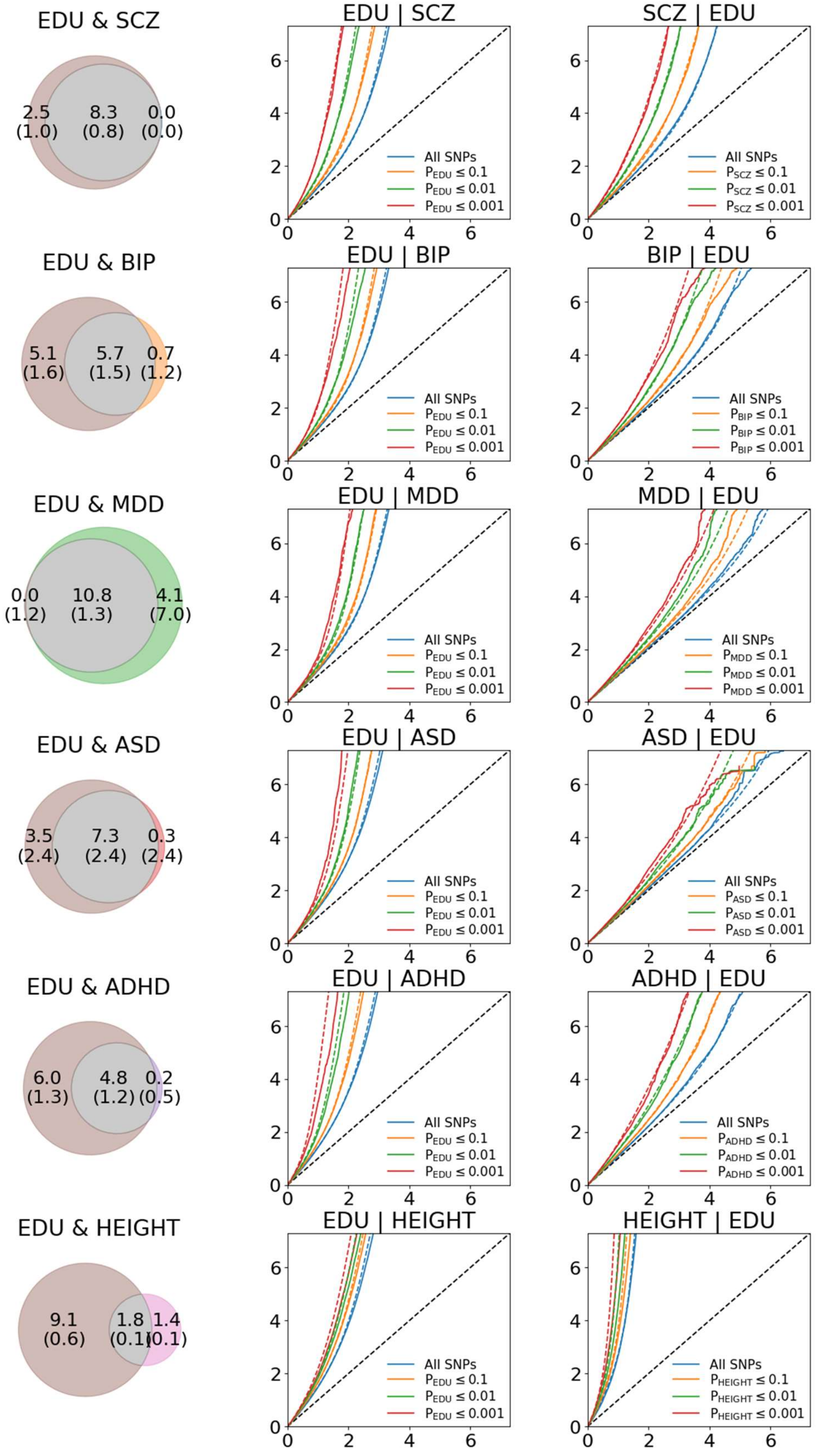

Venn Diagrams and stratified QQ plots, visualizing conditional distribution of p-values.

Supplementary Figure 13g. Venn diagrams and conditional cross-trait QQ plots for height

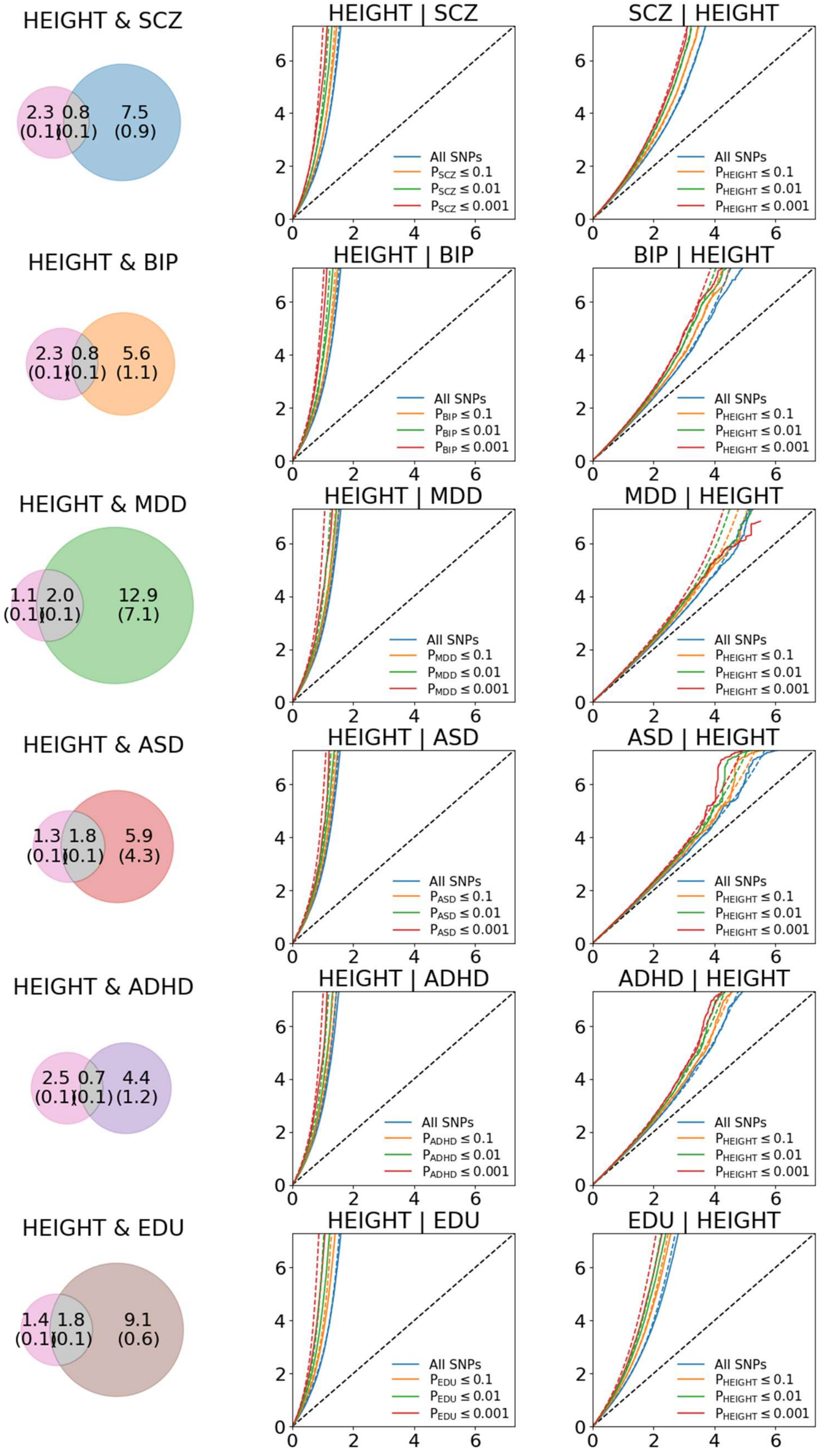

Venn Diagrams and stratified QQ plots, visualizing conditional distribution of p-values.

Supplementary Figure 13h. Venn diagrams and conditional cross-trait QQ plots for autoimmune disorders

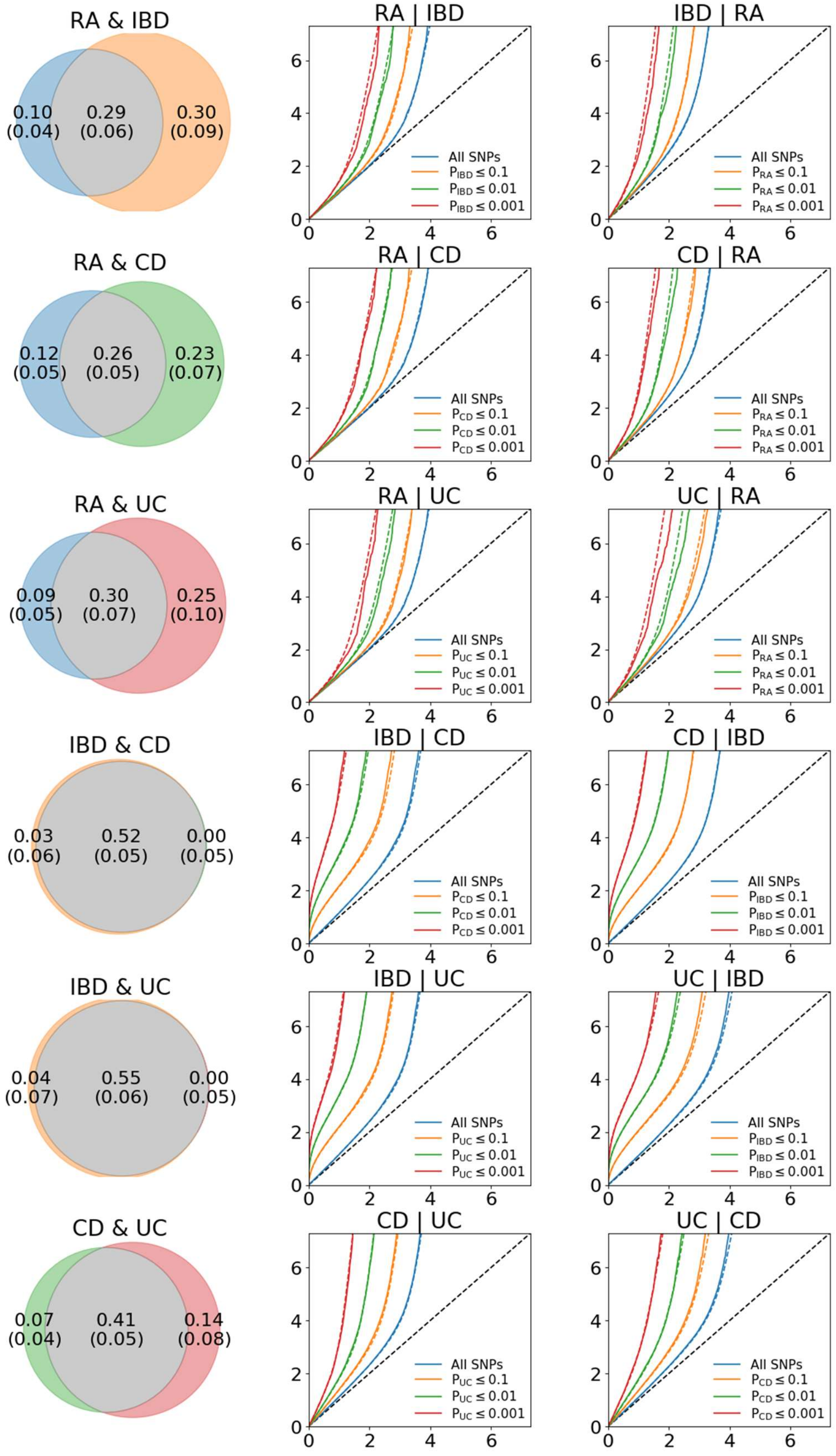

Venn Diagrams and stratified QQ plots, visualizing conditional distribution of p-values.

Supplementary Figure 13i. Venn diagrams and conditional cross-trait QQ plots for anthropomorphic traits

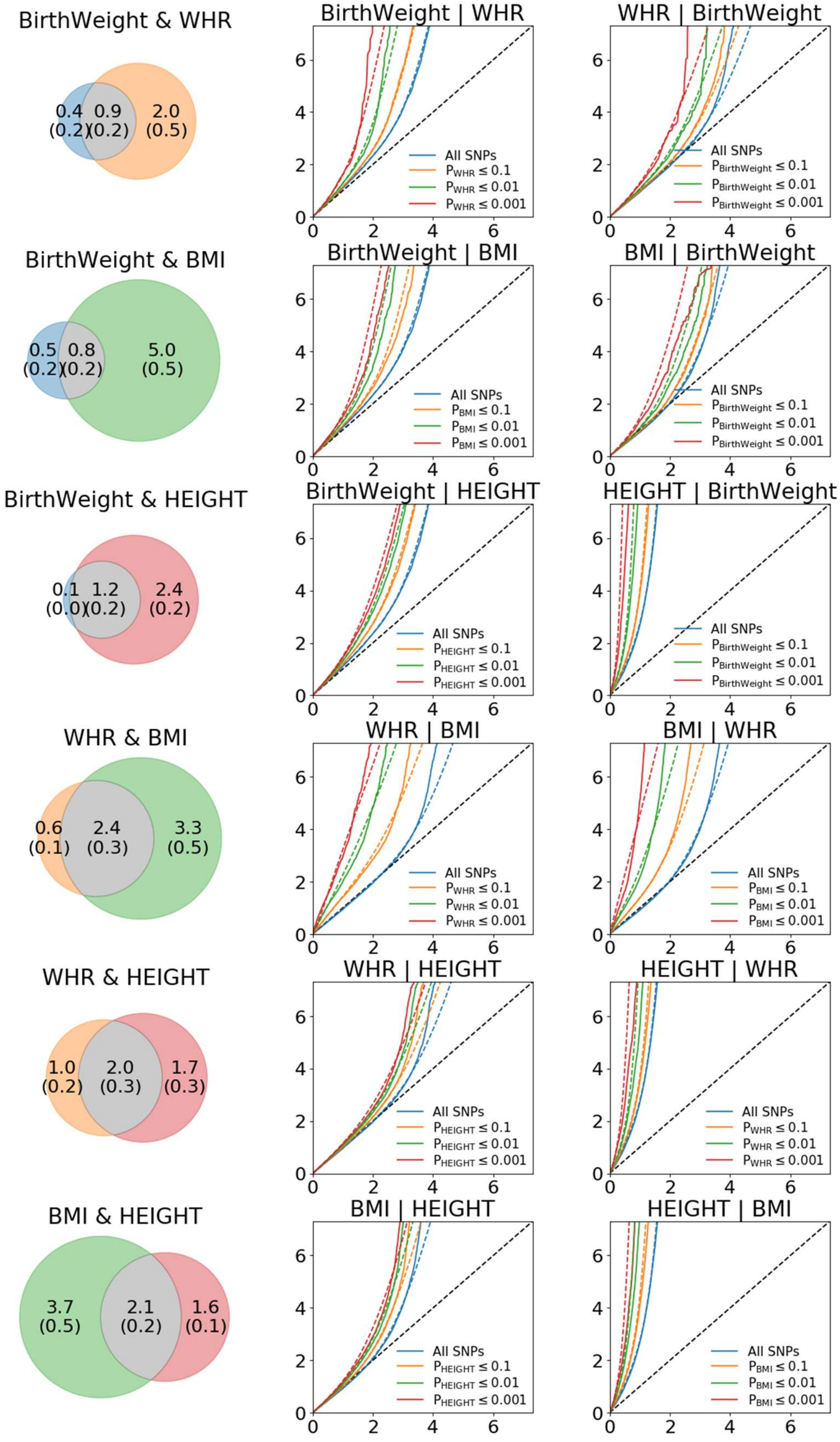

Venn Diagrams and stratified QQ plots, visualizing conditional distribution of p-values.

Supplementary Figure 14a. Observed and predicted bivariate density of GWAS association statistics, schizophrenia

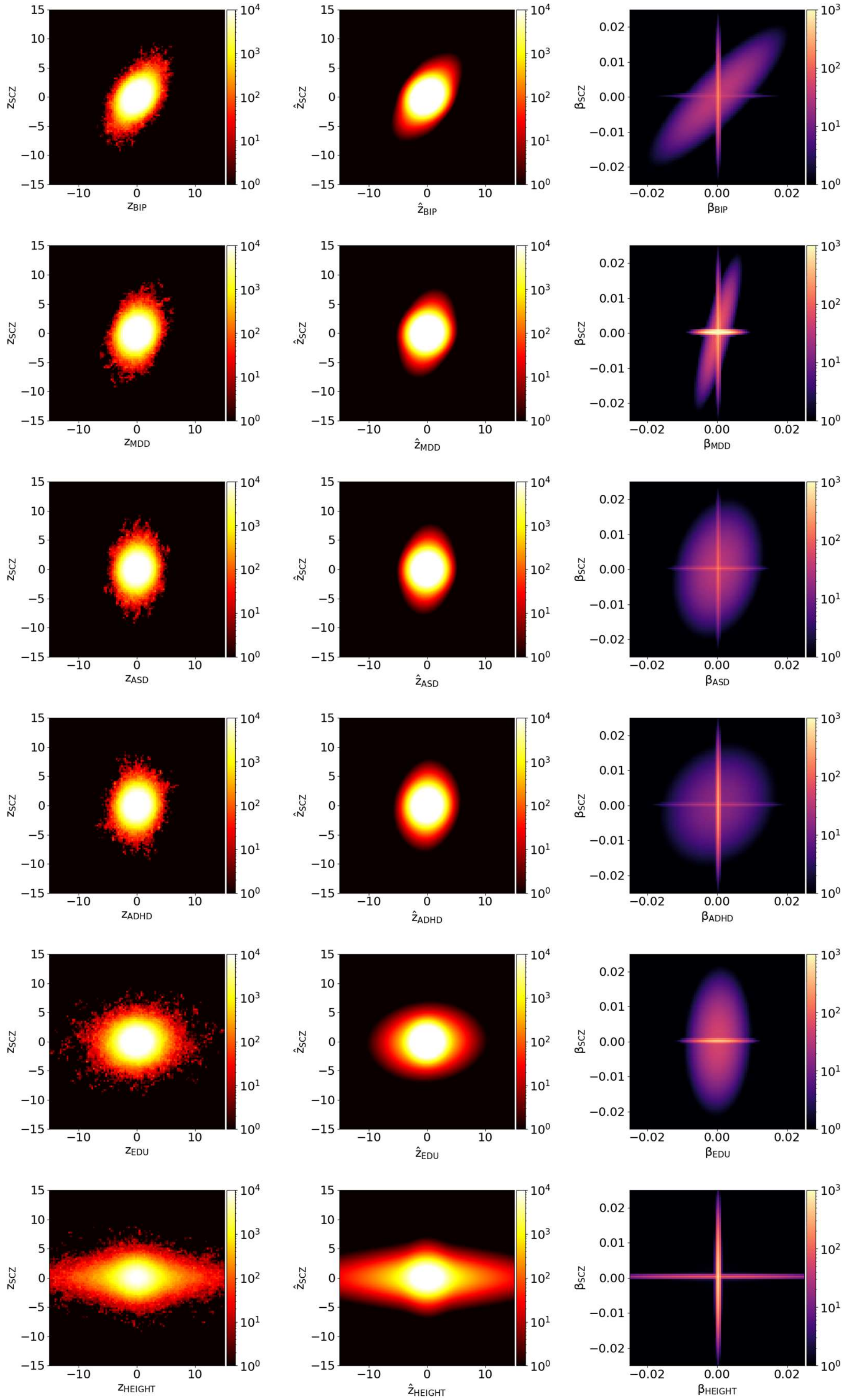

Supplementary Figure 14b. Observed and predicted bivariate density of GWAS association statistics, bipolar disorder

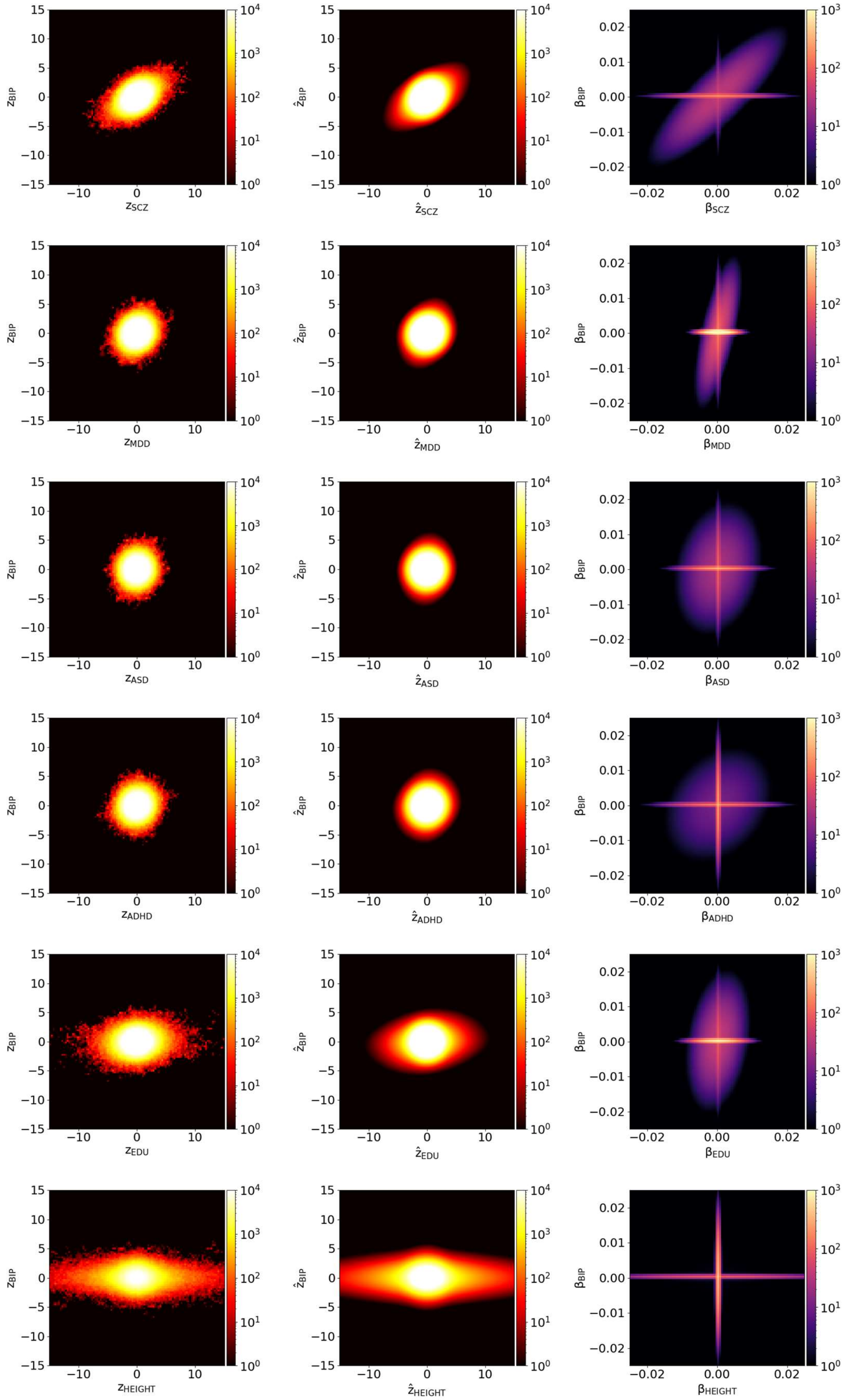

Supplementary Figure 14c. Observed and predicted bivariate density of GWAS association statistics, major depressive disorder

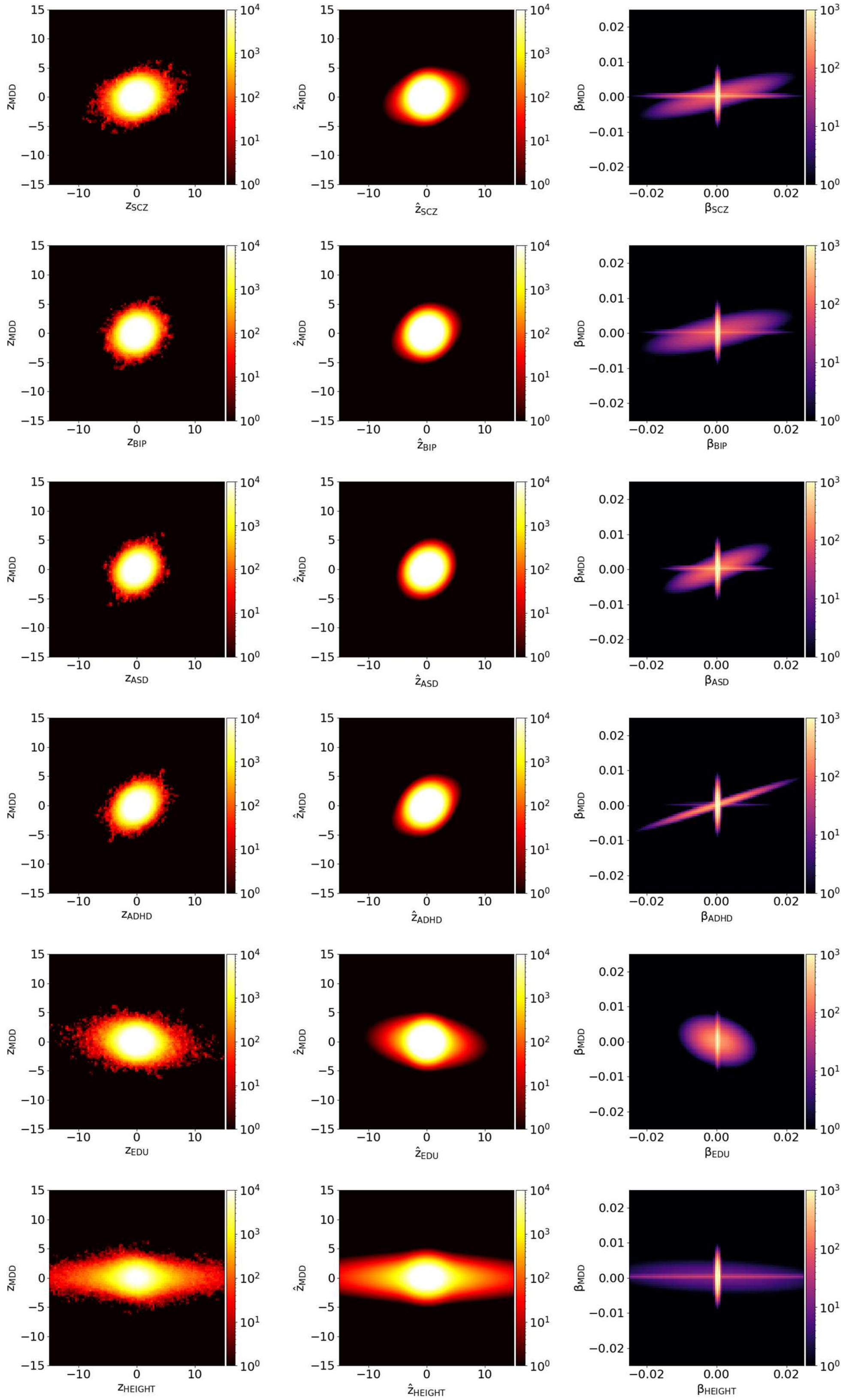

Supplementary Figure 14d. Observed and predicted bivariate density of GWAS association statistics, autism spectrum disorder

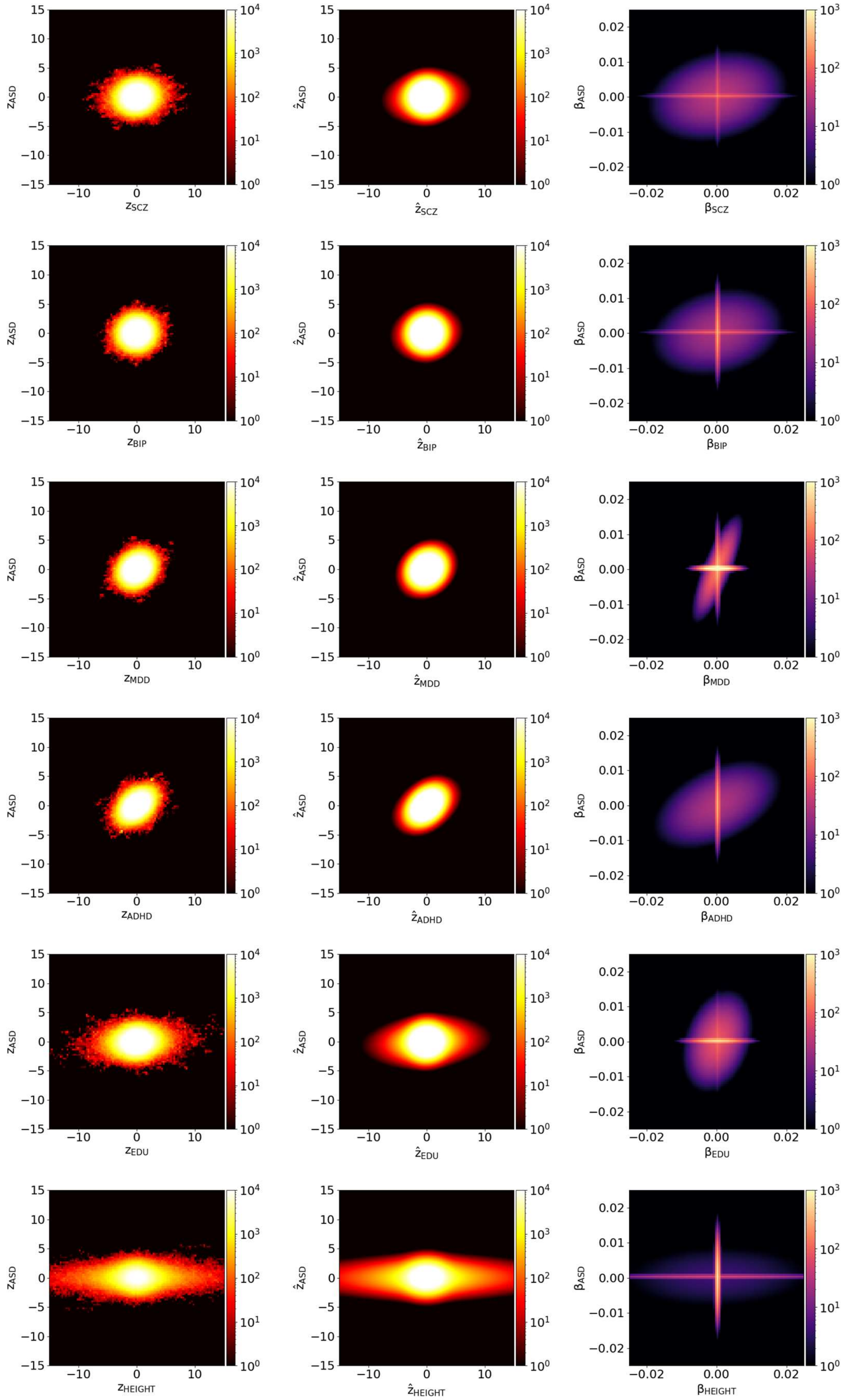

Supplementary Figure 14e. Observed and predicted bivariate density of GWAS association statistics, attention deficit / hyperactivity disorder

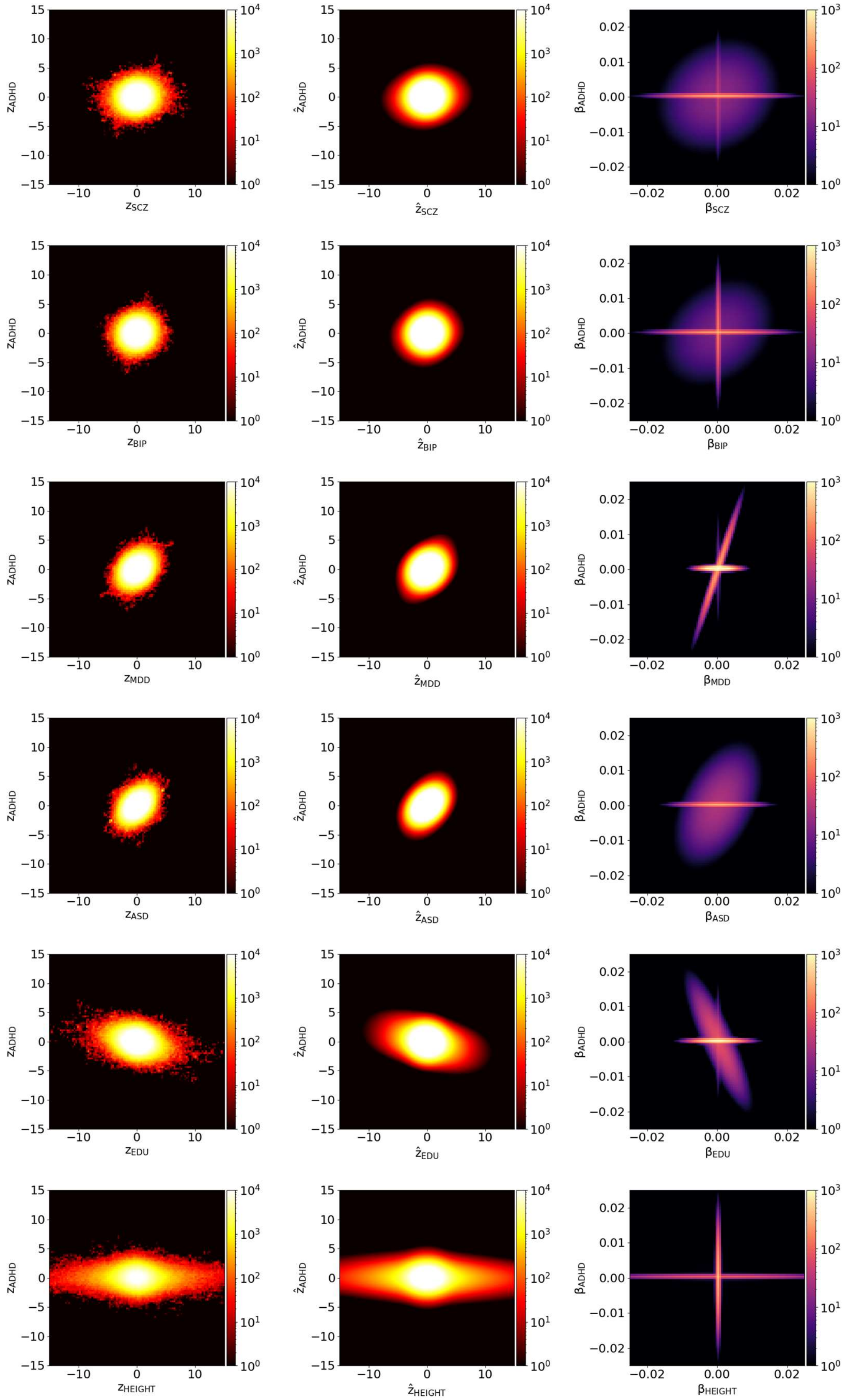

Supplementary Figure 14f. Observed and predicted bivariate density of GWAS association statistics, educational attainment

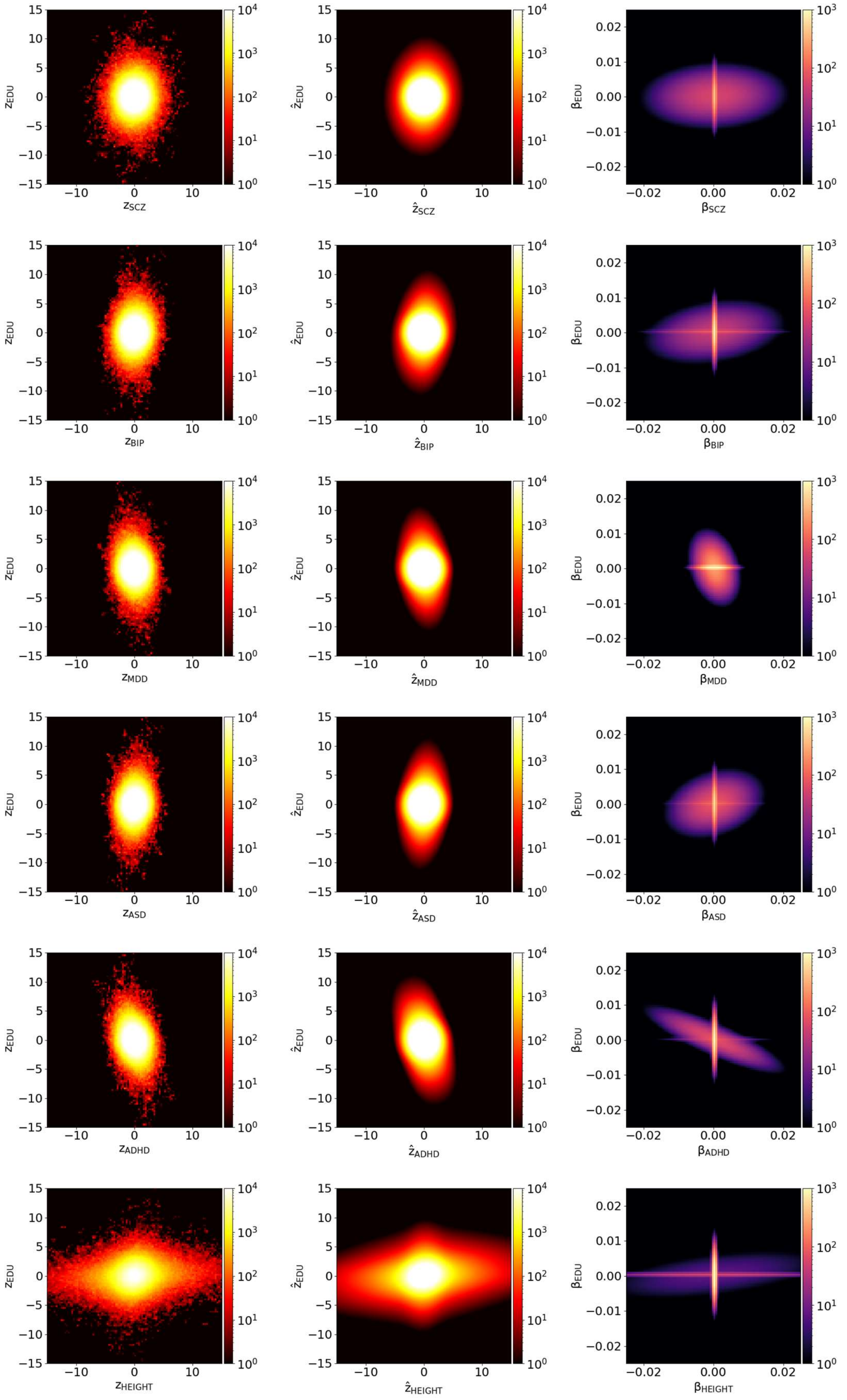

Supplementary Figure 14g. Observed and predicted bivariate density of GWAS association statistics, height

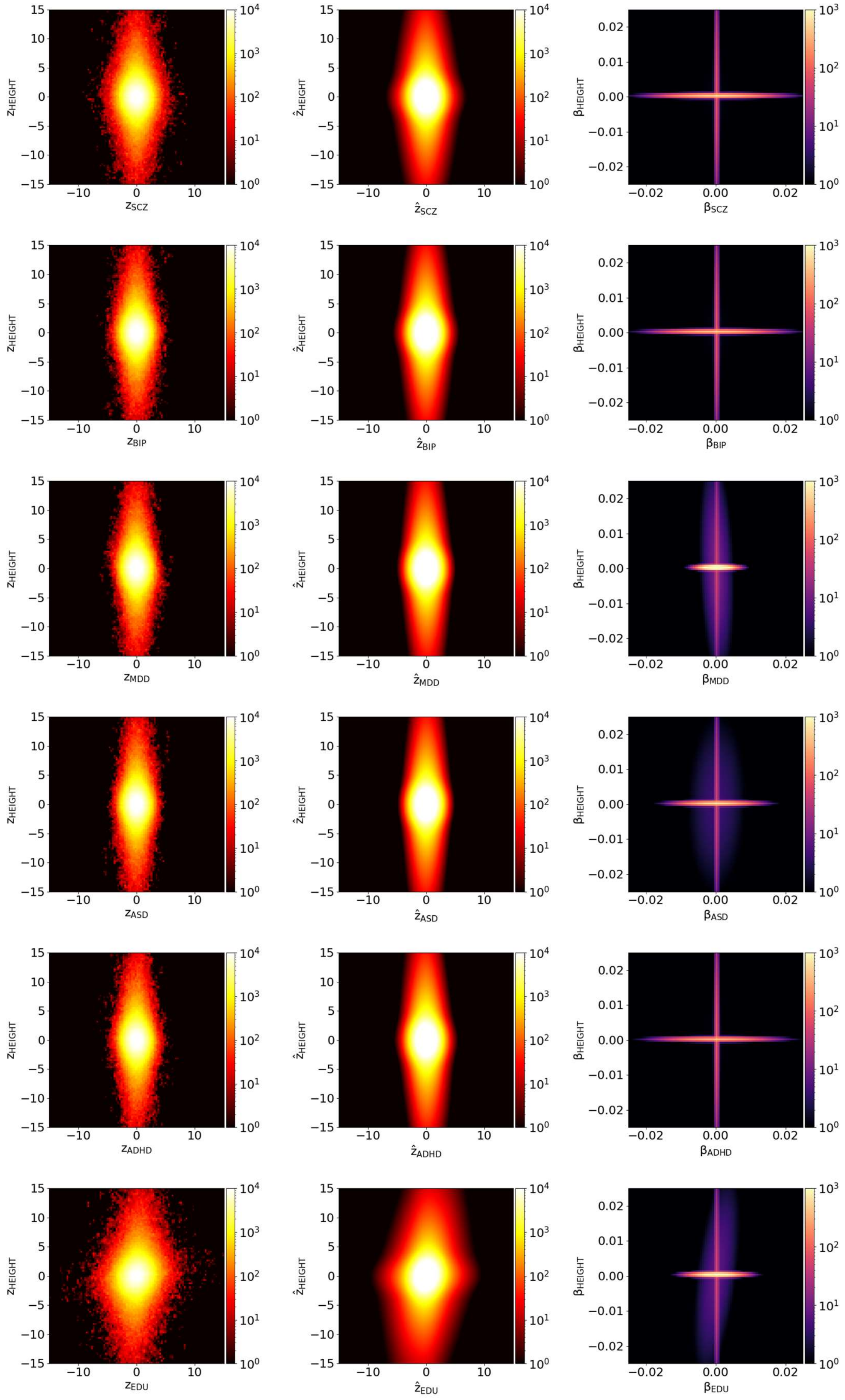

Supplementary Figure 14h. Observed and predicted bivariate density of GWAS association statistics, autoimmune disorders

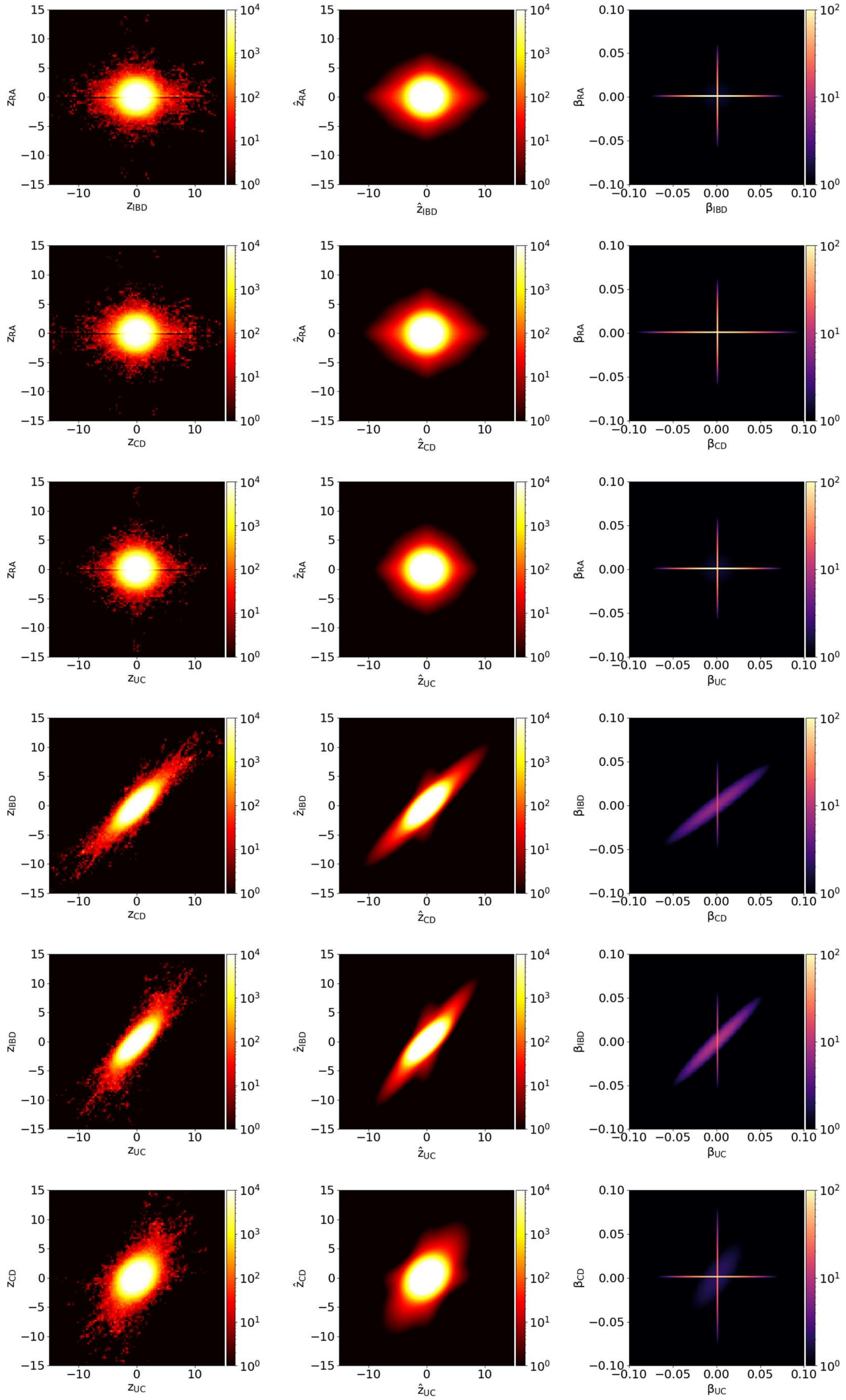

Supplementary Figure 14i. Observed and predicted bivariate density of GWAS association statistics, anthropomorphic traits

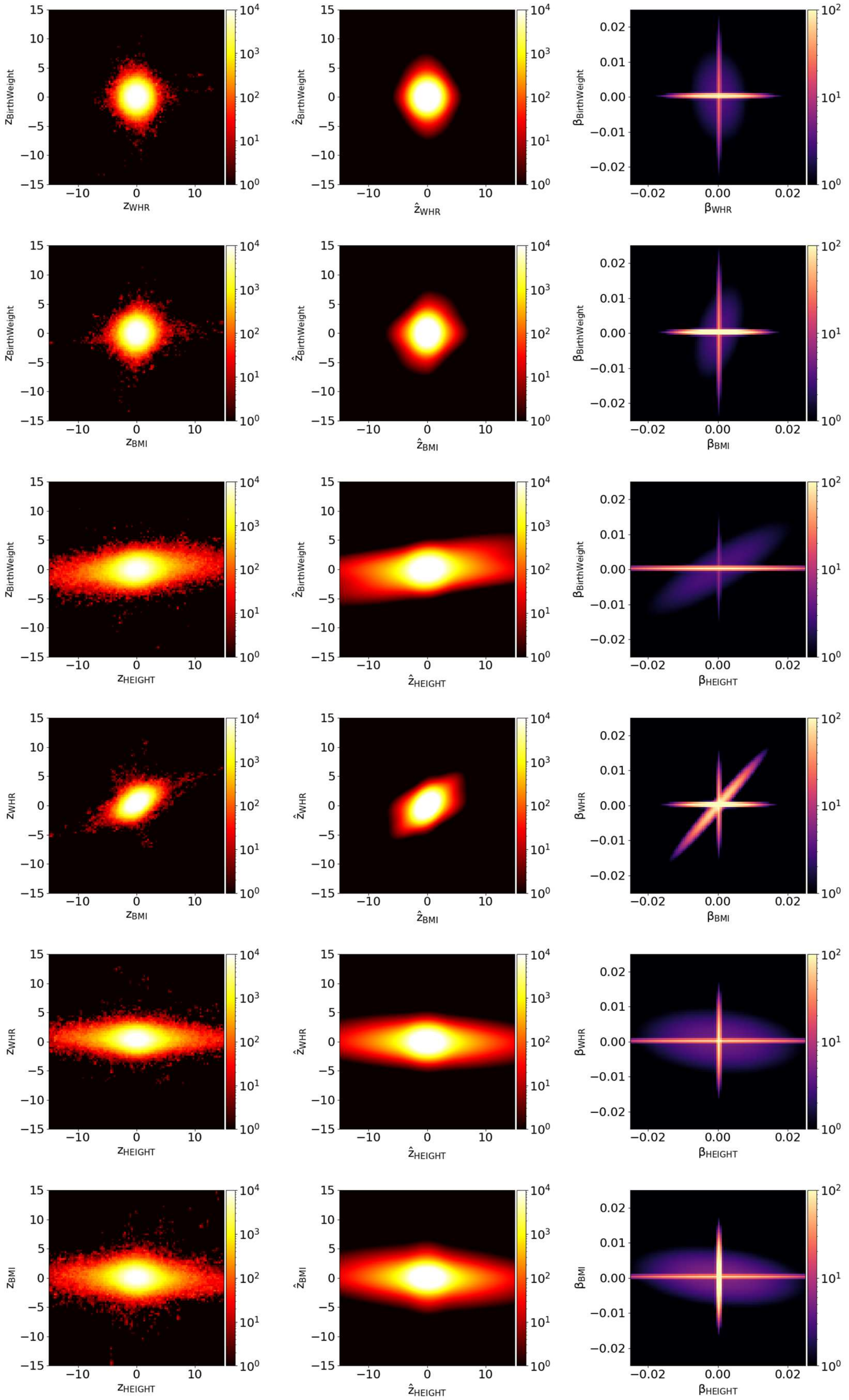

Supplementary Figure 15. Projected power plot for future GWAS sample sizes

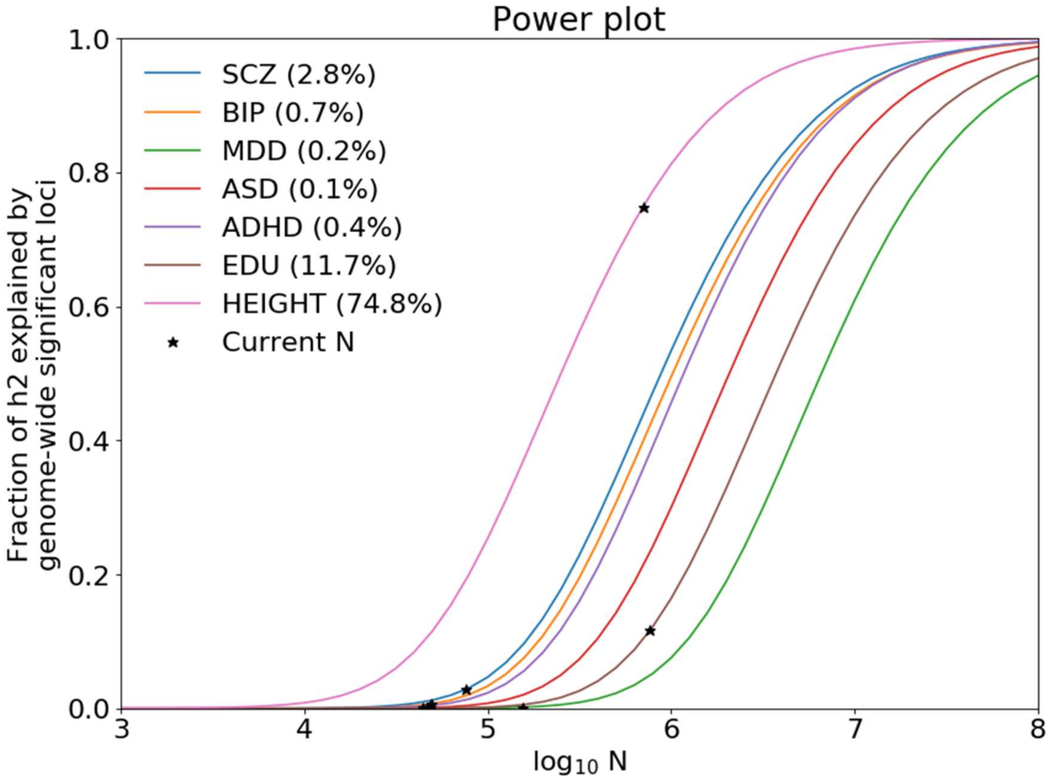

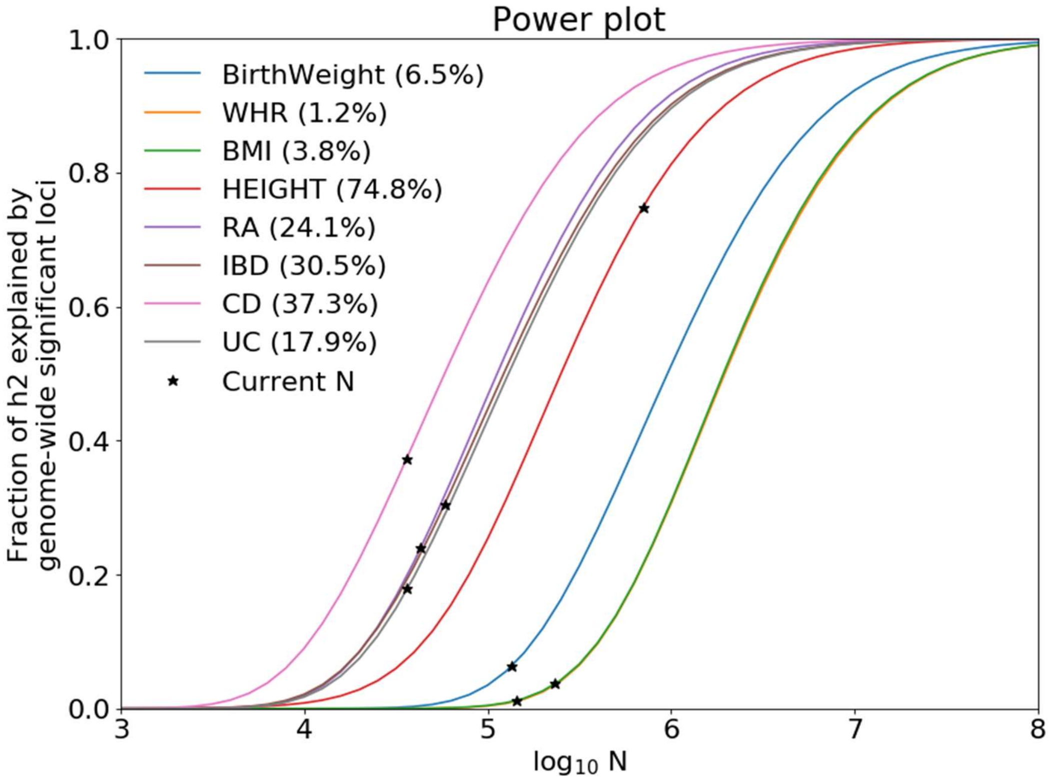

Proportion of SNP-heritability, captured by genome-wide significant SNPs, projected to the future GWAS sample size, N. Values for current GWAS sample sizes are shown in parentheses.

Supplementary Figure 16. Univariate Q-Q plots

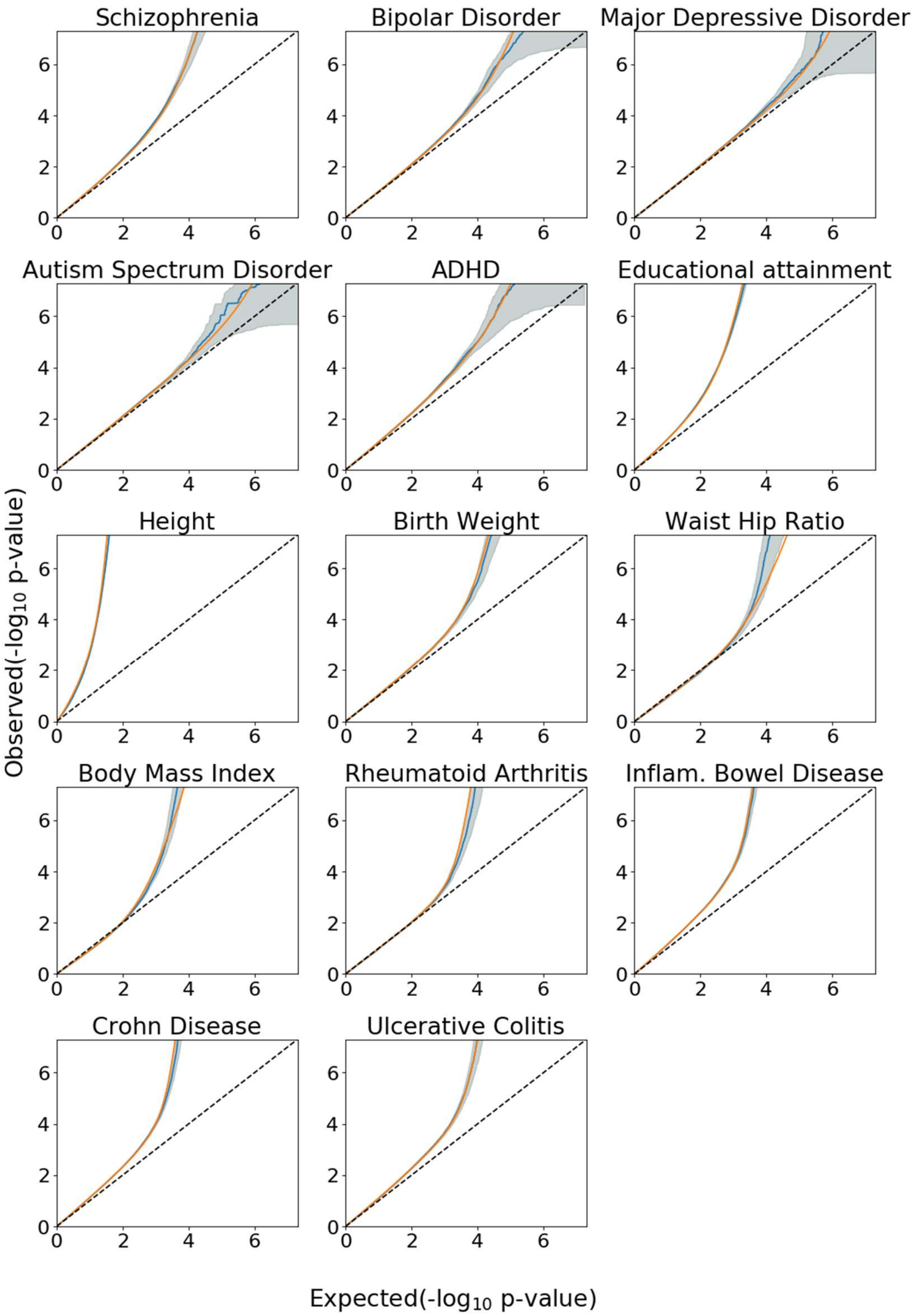

QQ plots for observed GWAS p-values (in blue) and model prediction (in orange). The dashed line is the expected QQ plot under null (no SNPs associated with the phenotype). The vertical axis is limited to the standard GWAS threshold of $p<5\times10^{-8}$, to highlight behavior of polygenic component. Points on the QQ plot are weighted according to LD structure, using n=64 iterations of random pruning at LD threshold r2=0.1. QQ plots are calculated on the entire set SNPs available in GWAS, constrained to LD Score Regression reference panel of 9.997.231 SNPs.

Supplementary Figure 17a. Q-Q plots of SNPs partitioned by MAF and LD score, schizophrenia

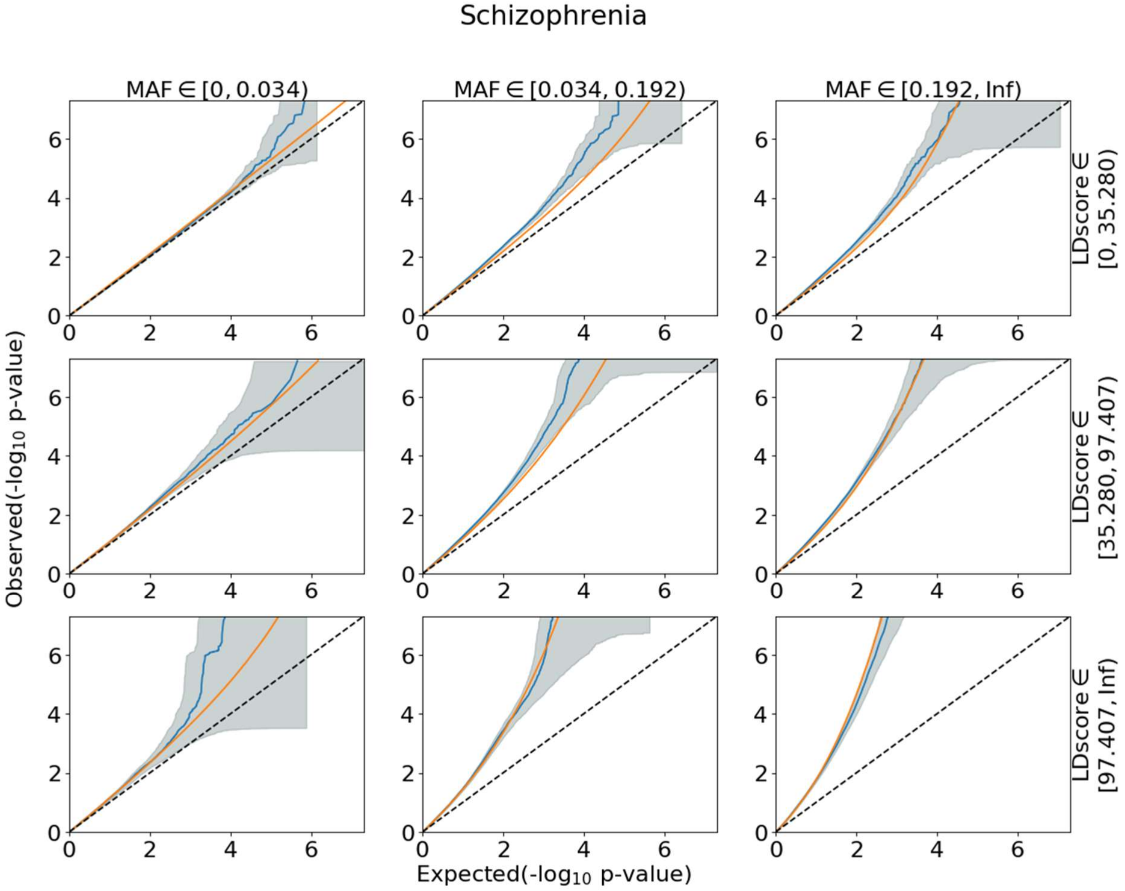

QQ plots for subsets of SNPs, showing observed GWAS p-values (in blue) and model prediction (in orange). All SNPs were partitioned into 9 groups according to minor allele frequency (MAF) and total LD score. The model was fit only once, so that all model predictions are based on the same set of parameters. Observed QQ plots show a stronger GWAS signal for SNPs with to higher MAF and higher LD score. Model's prediction follows the same pattern, indicating that model correctly captures dependency of GWAS association statistics on MAF and Total LD score.

Supplementary Figure 17b. Q-Q plots of SNPs partitioned by MAF and LD score, bipolar disorder

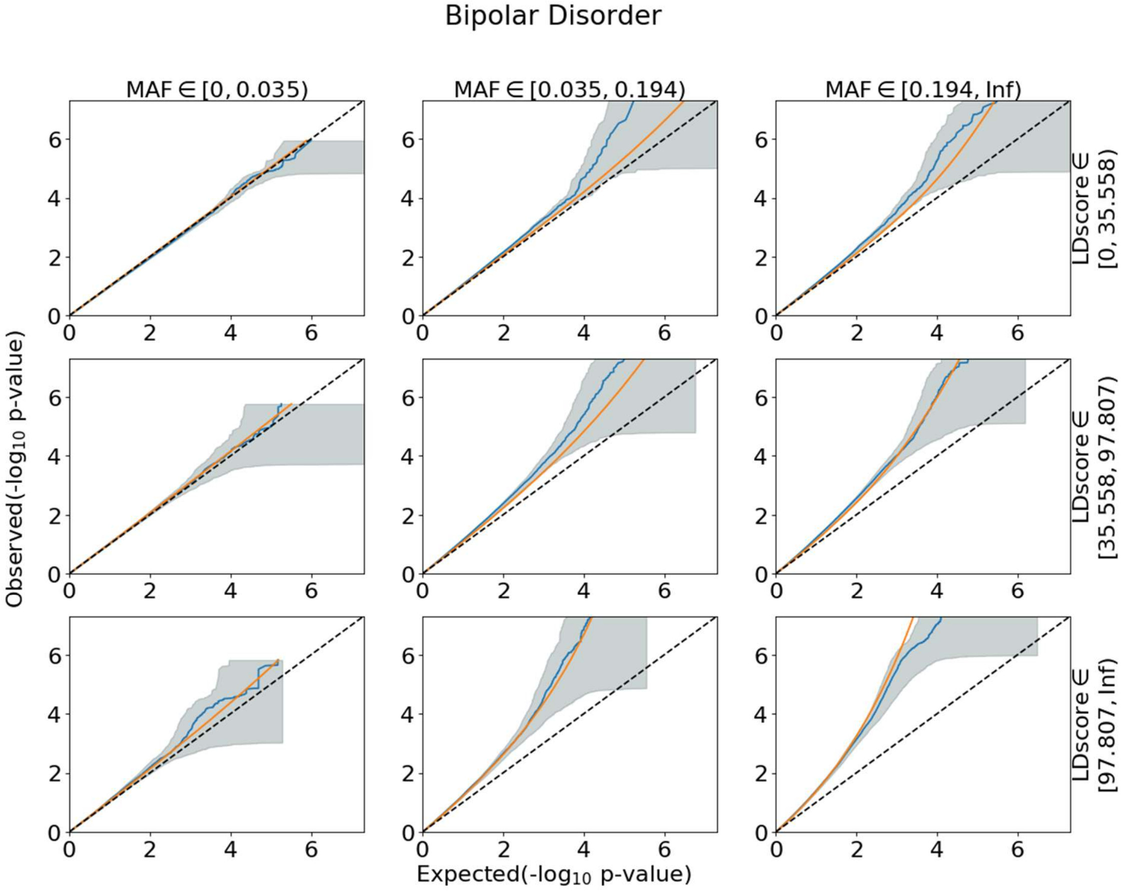

QQ plots of SNPs partitioned by MAF and LD score. Appearance of the Q-Q plot is as described on the previous figure.

Supplementary Figure 17c. Q-Q plots of SNPs partitioned by MAF and LD score, major depressive disorder

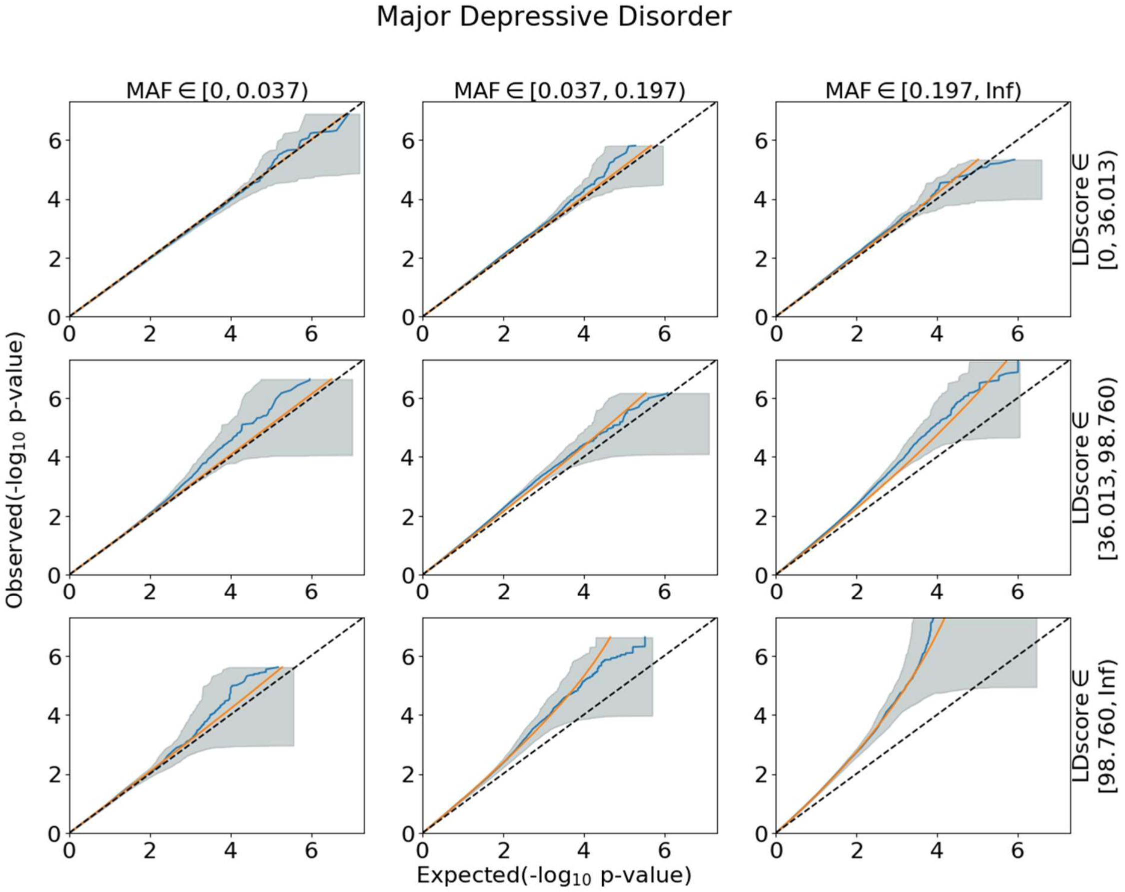

QQ plots of SNPs partitioned by MAF and LD score. Appearance of the Q-Q plot is as described on the previous figure.

Supplementary Figure 17d. Q-Q plots of SNPs partitioned by MAF and LD score, autism spectrum disorder

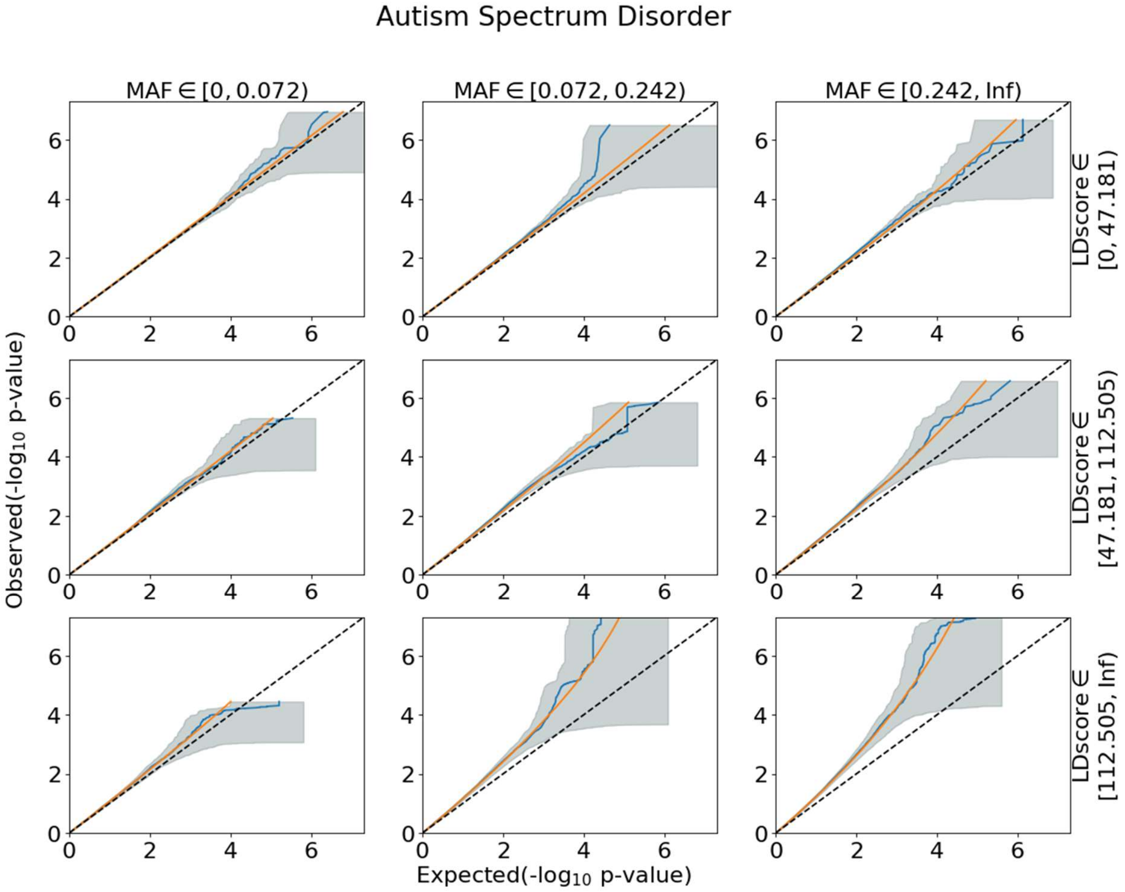

QQ plots of SNPs partitioned by MAF and LD score. Appearance of the Q-Q plot is as described on the previous figure.

Supplementary Figure 17e. Q-Q plots of SNPs partitioned by MAF and LD score, ADHD

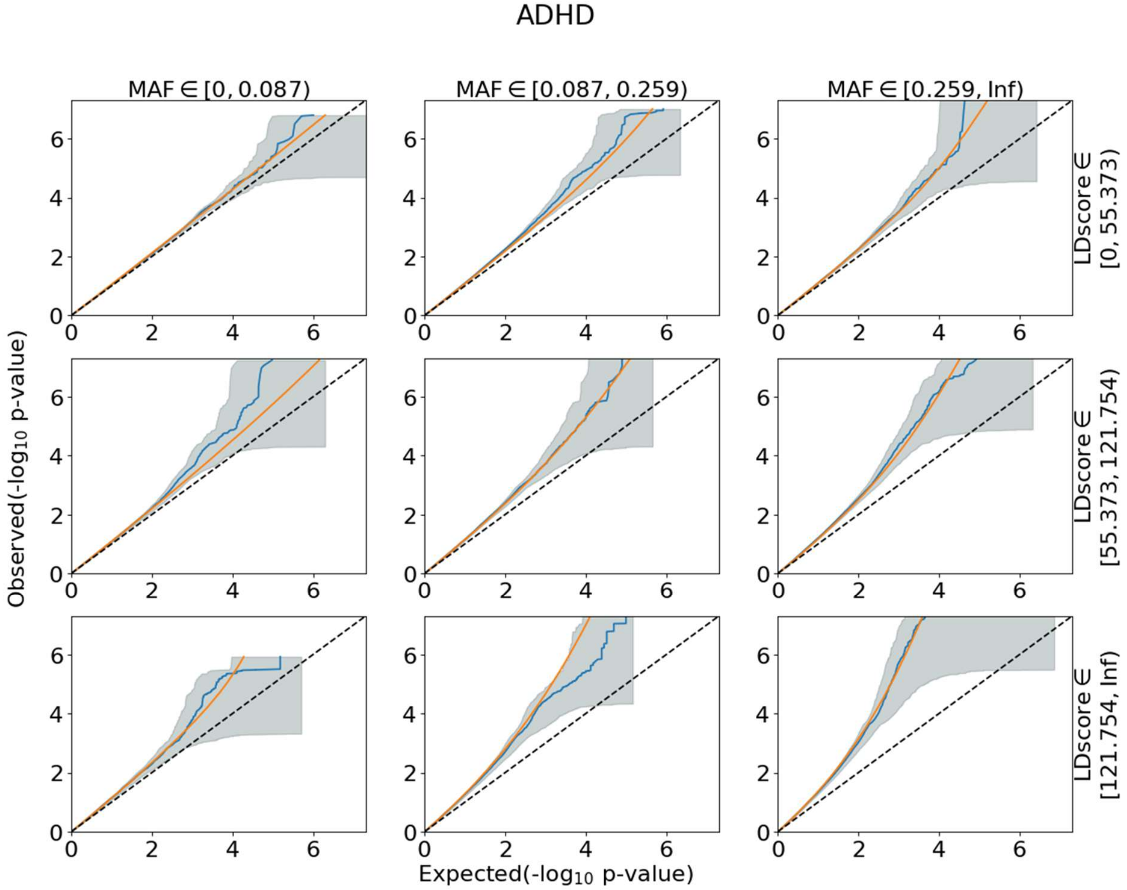

QQ plots of SNPs partitioned by MAF and LD score. Appearance of the Q-Q plot is as described on the previous figure.

Supplementary Figure 17f. Q-Q plots of SNPs partitioned by MAF and LD score, educational attainment

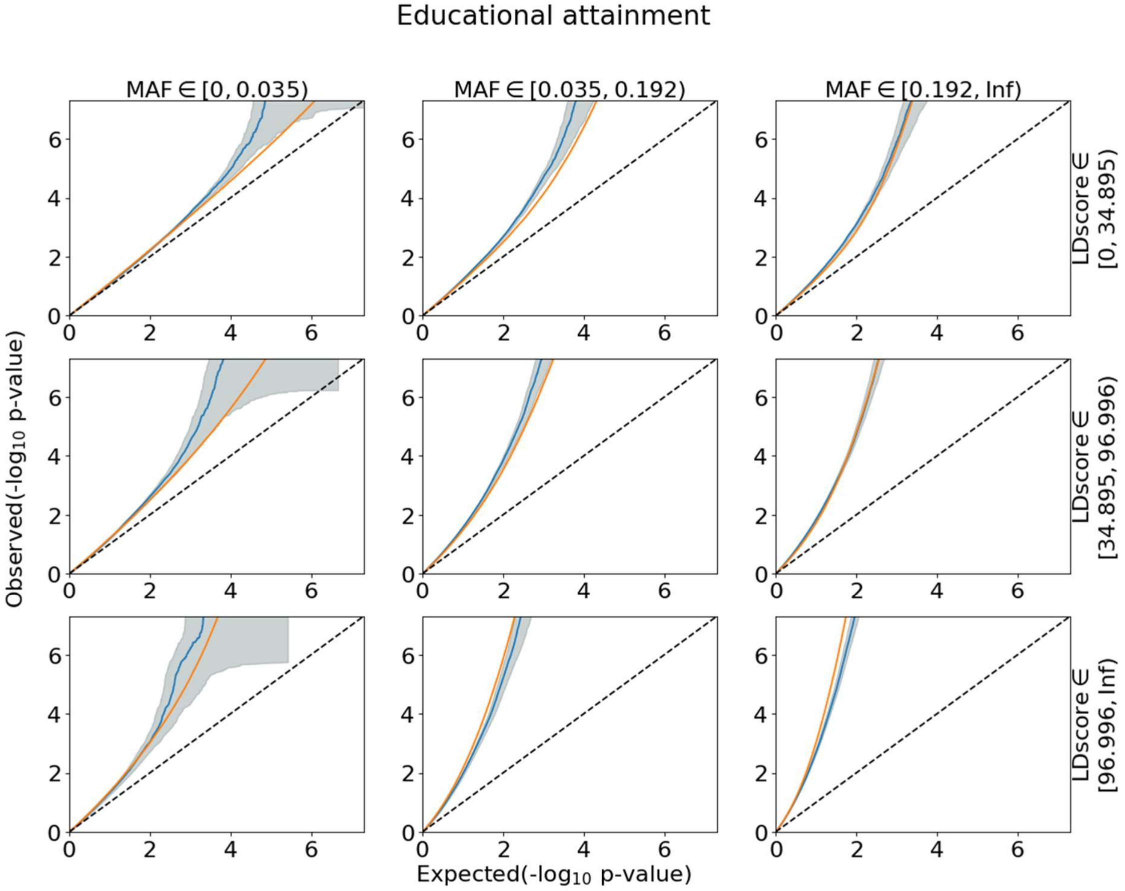

QQ plots of SNPs partitioned by MAF and LD score. Appearance of the Q-Q plot is as described on the previous figure.

Supplementary Figure 17g. Q-Q plots of SNPs partitioned by MAF and LD score, height

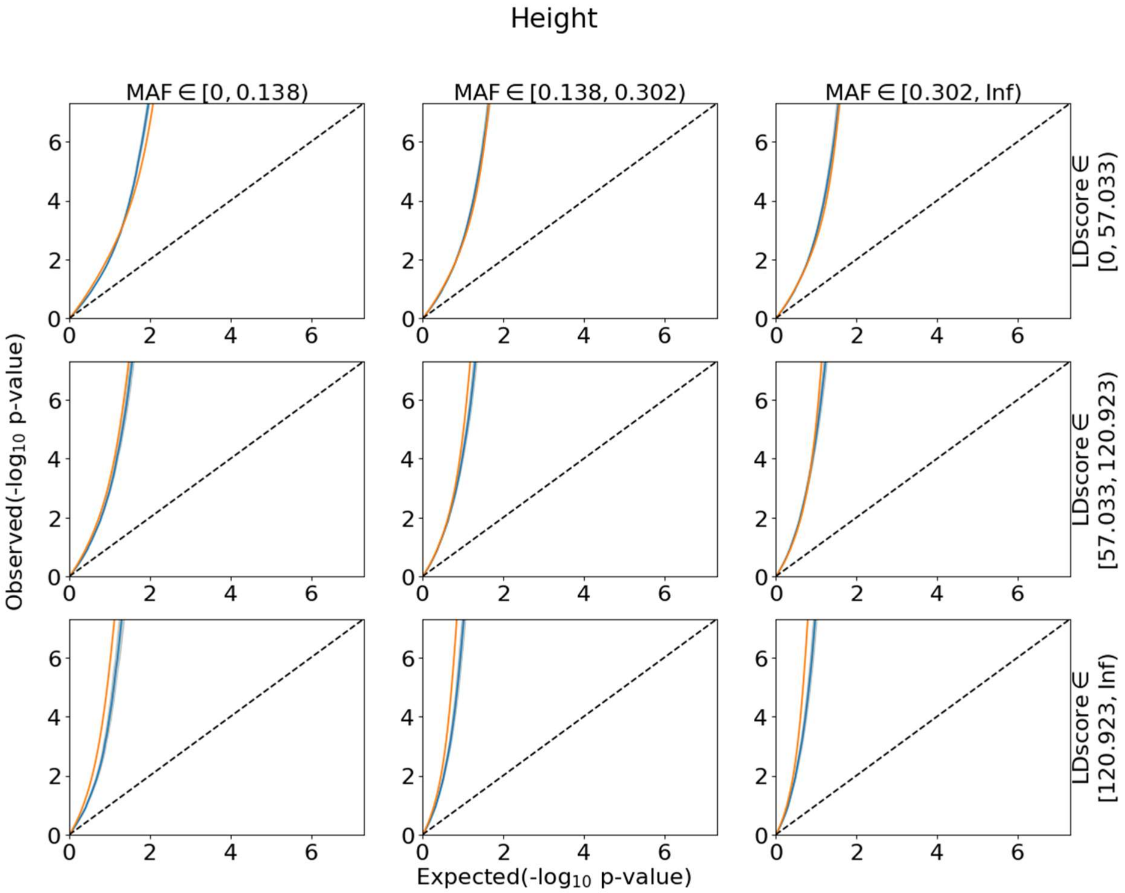

QQ plots of SNPs partitioned by MAF and LD score. Appearance of the Q-Q plot is as described on the previous figure.

Supplementary Figure 17h. Q-Q plots of SNPs partitioned by MAF and LD score, birth weight

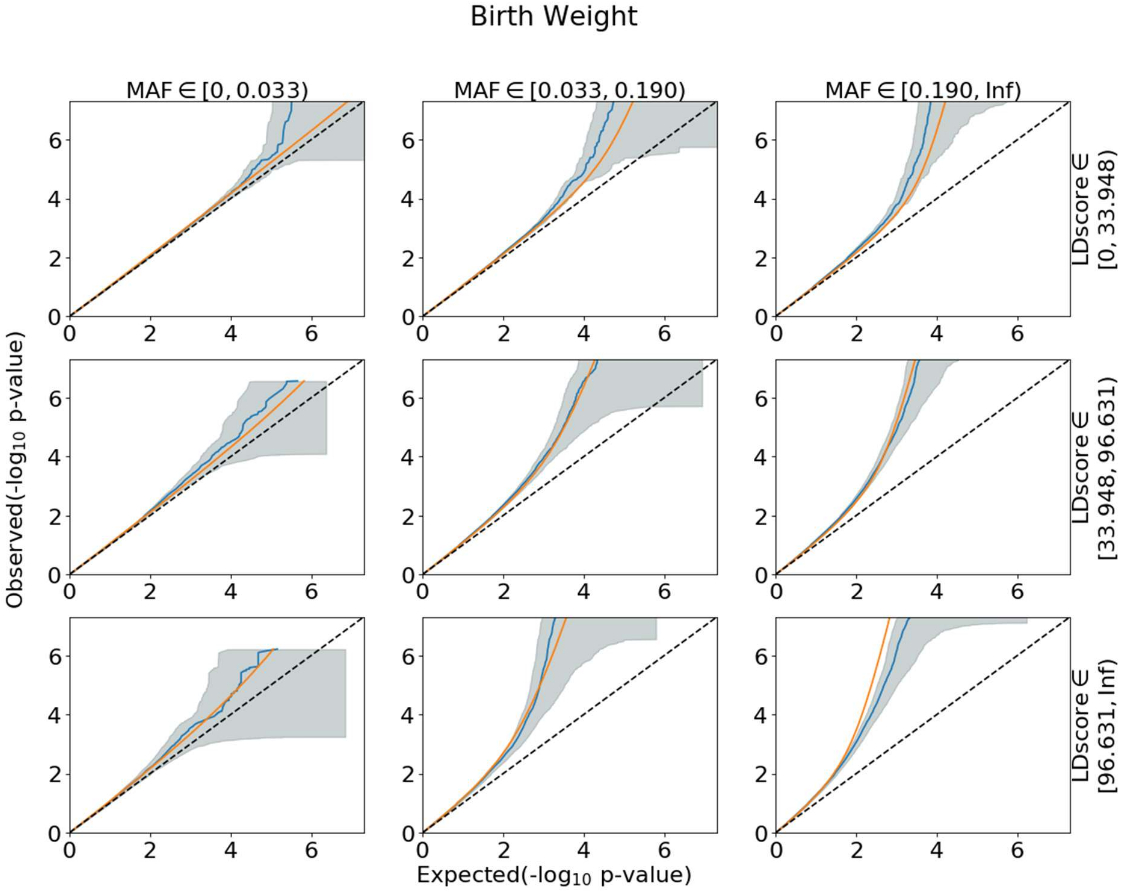

QQ plots of SNPs partitioned by MAF and LD score. Appearance of the Q-Q plot is as described on the previous figure.

Supplementary Figure 17i. Q-Q plots of SNPs partitioned by MAF and LD score, body mass index

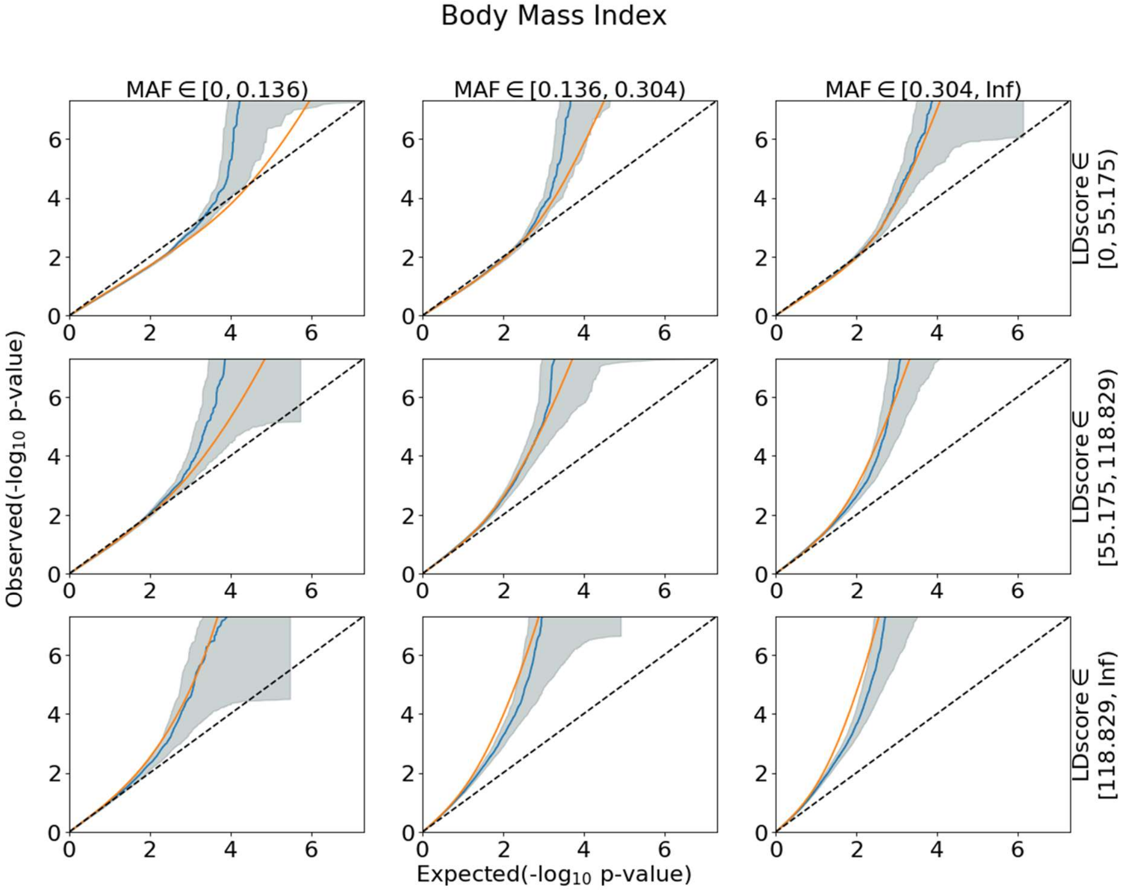

QQ plots of SNPs partitioned by MAF and LD score. Appearance of the Q-Q plot is as described on the previous figure.

Supplementary Figure 17j. Q-Q plots of SNPs partitioned by MAF and LD score, waist hip ratio

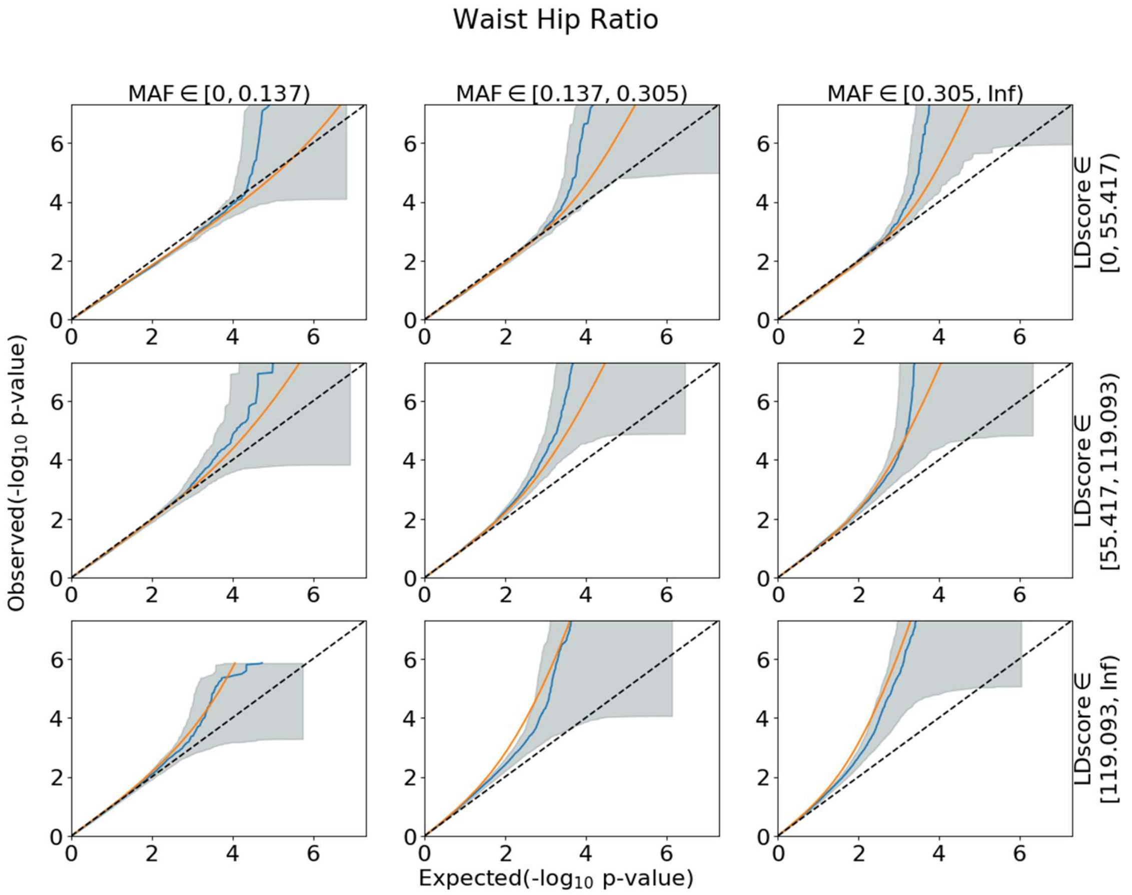

QQ plots of SNPs partitioned by MAF and LD score. Appearance of the Q-Q plot is as described on the previous figure.

Supplementary Figure 17k. Q-Q plots of SNPs partitioned by MAF and LD score, rheumatoid arthritis

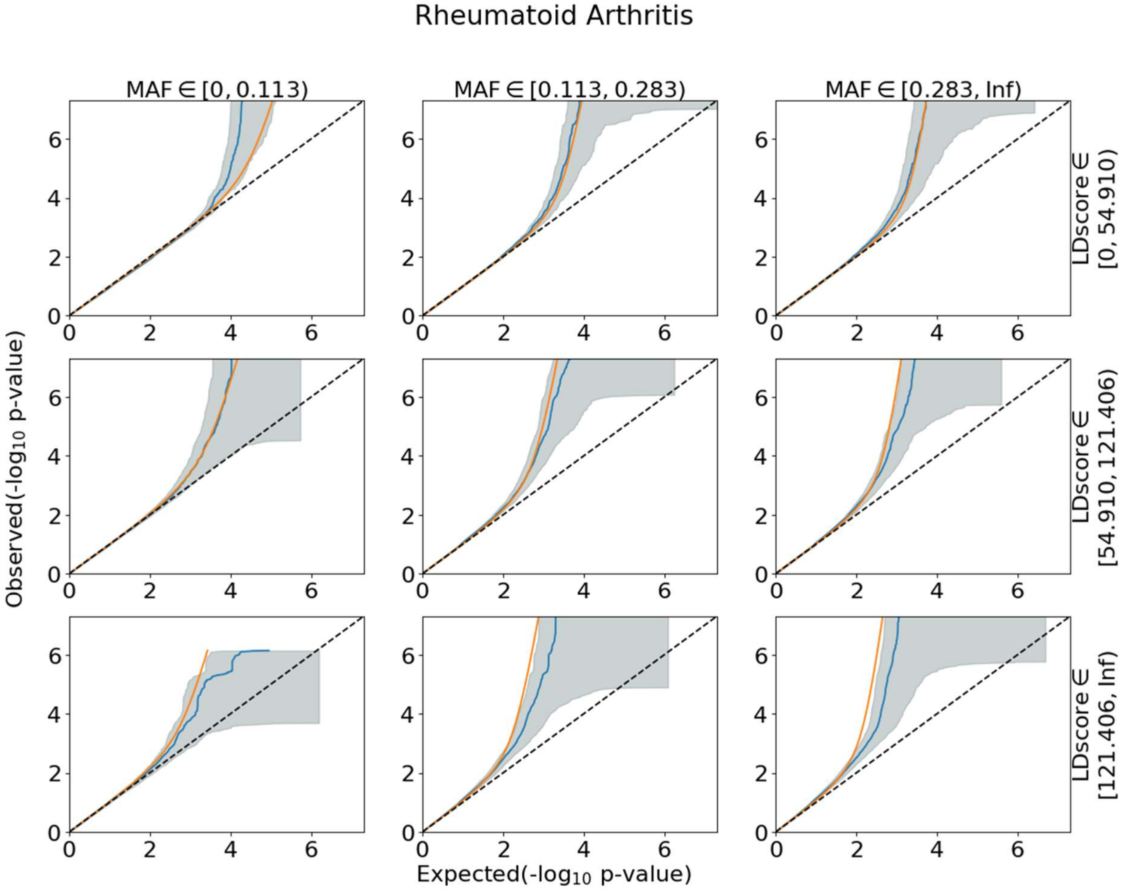

QQ plots of SNPs partitioned by MAF and LD score. Appearance of the Q-Q plot is as described on the previous figure.

Supplementary Figure 17l. Q-Q plots of SNPs partitioned by MAF and LD score, inflammatory bowel disease

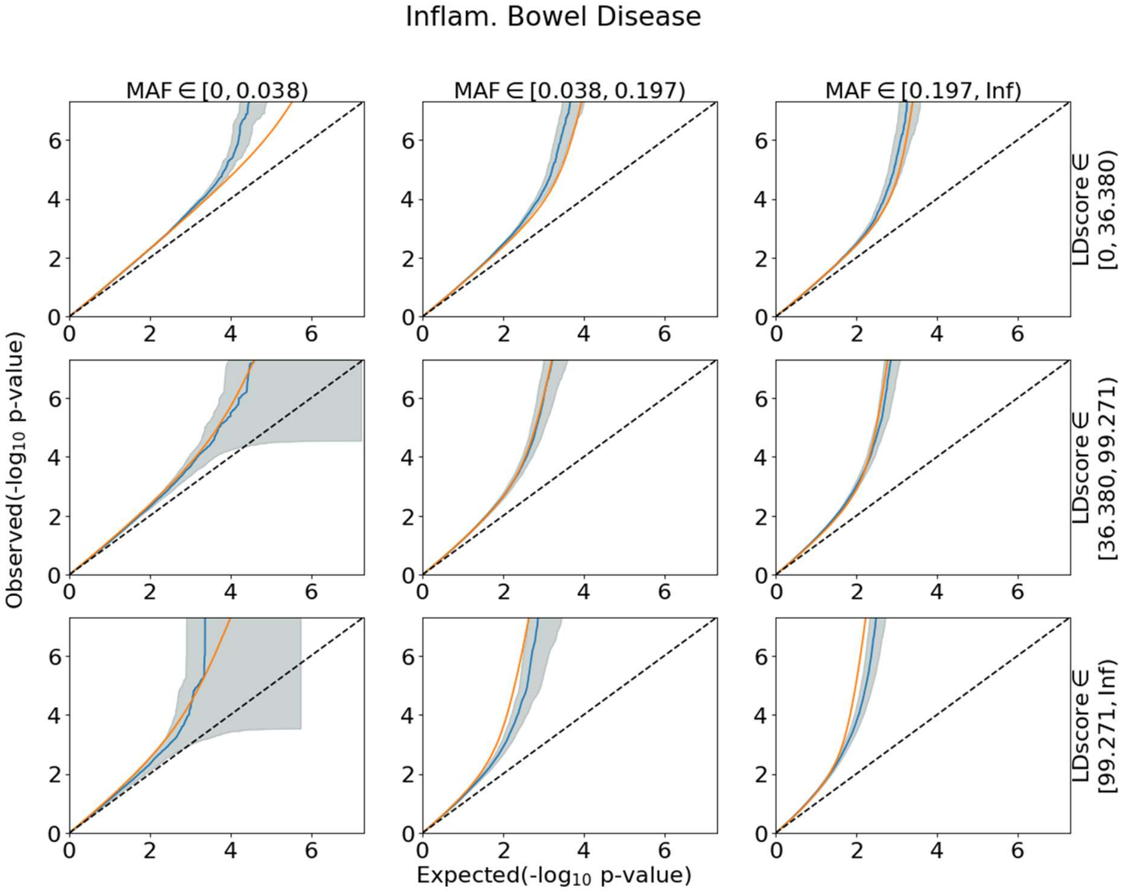

QQ plots of SNPs partitioned by MAF and LD score. Appearance of the Q-Q plot is as described on the previous figure.

Supplementary Figure 17m. Q-Q plots of SNPs partitioned by MAF and LD score, Crohn's disease

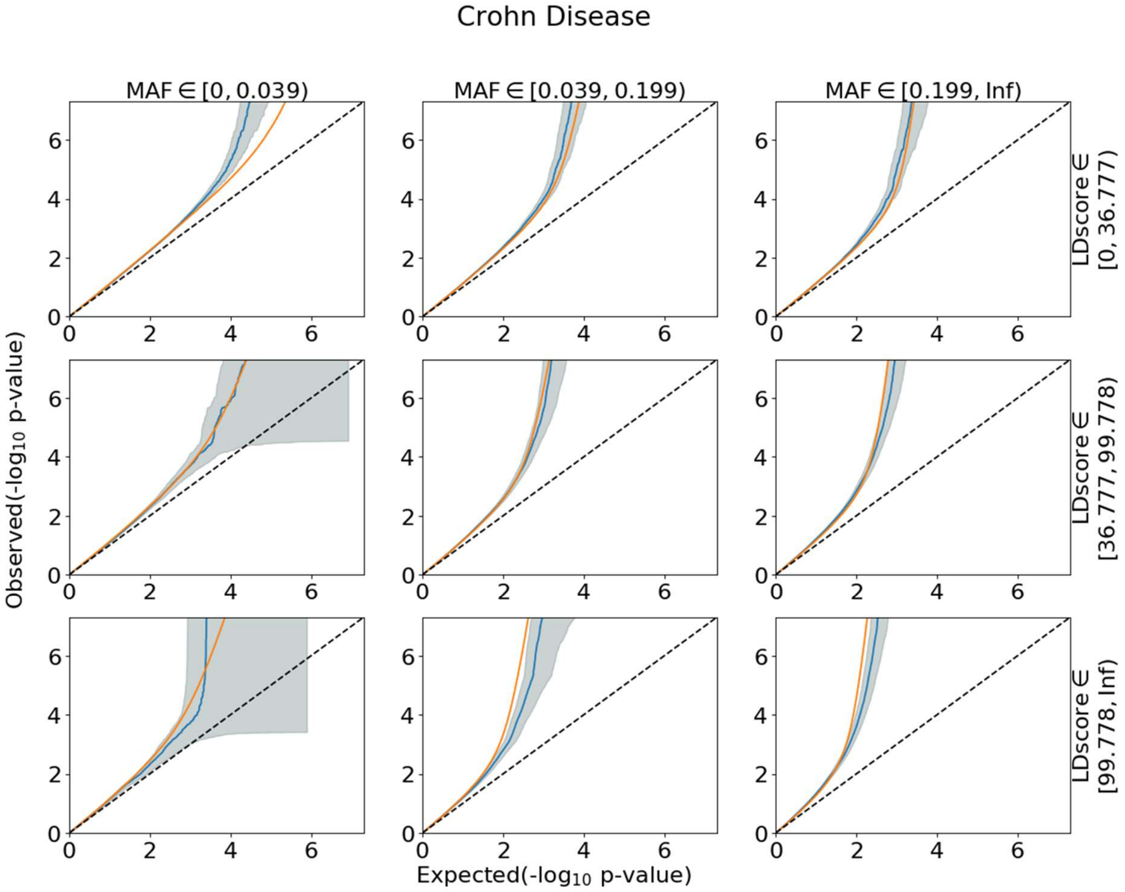

QQ plots of SNPs partitioned by MAF and LD score. Appearance of the Q-Q plot is as described on the previous figure.

Supplementary Figure 17n. Q-Q plots of SNPs partitioned by MAF and LD score, ulcerative colitis

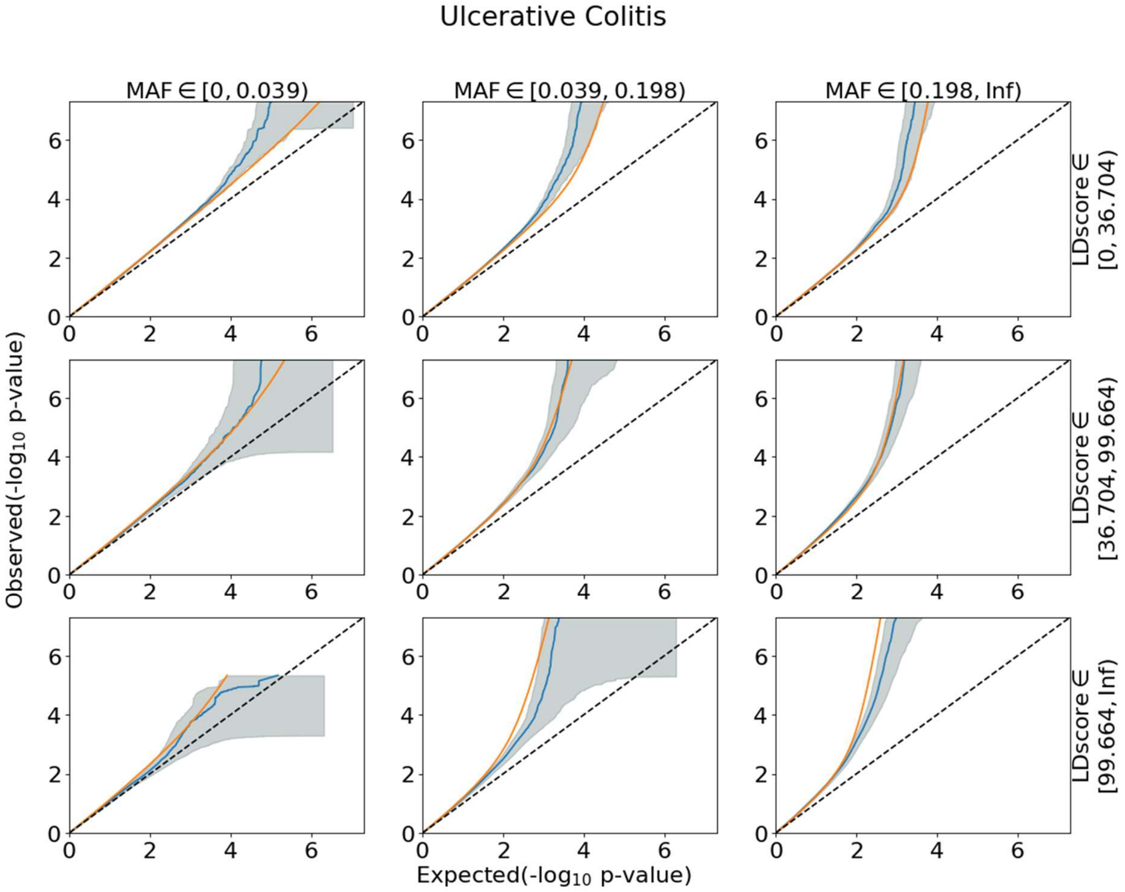

QQ plots of SNPs partitioned by MAF and LD score. Appearance of the Q-Q plot is as described on the previous figure.

Supplementary Figure 18. Univariate likelihood as a function of polygenicity parameter

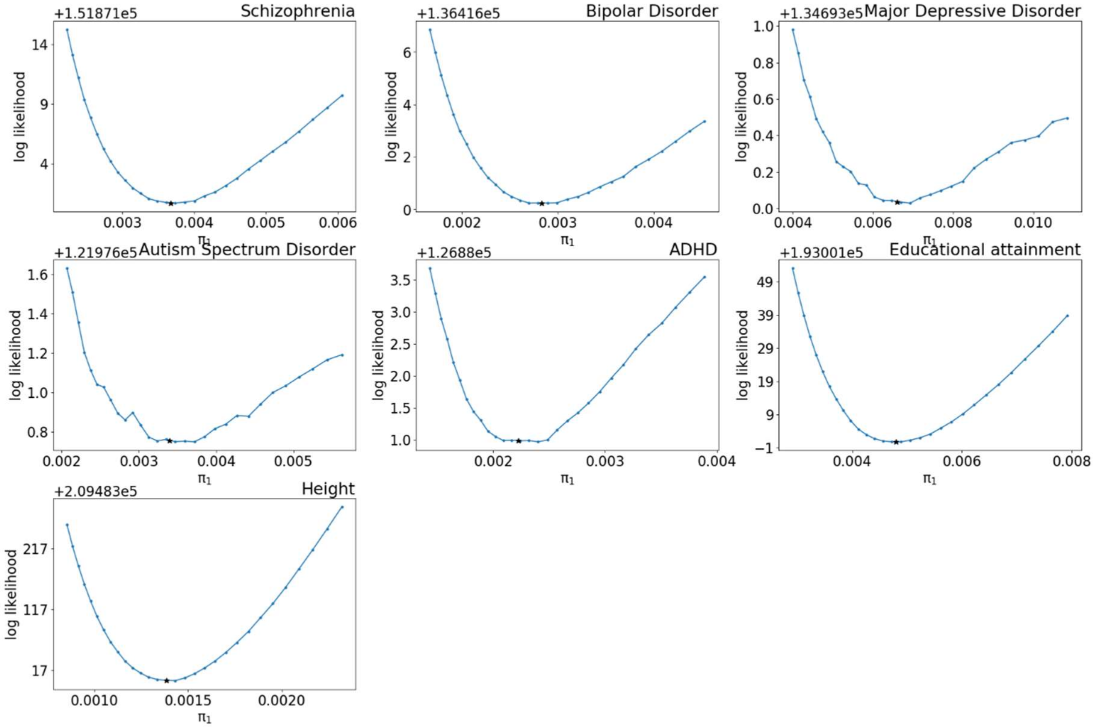

Log-likelihood of the univariate fit as a function of π_1 parameter. The remaining parameters of the model were constrained to the fitted values of heritability (h2) and variance distortion (σ_0^2). Asterix indicates fitted value of π_1 parameter.

Supplementary Figure 19. Bivariate likelihood as a function of polygenic overlap parameter

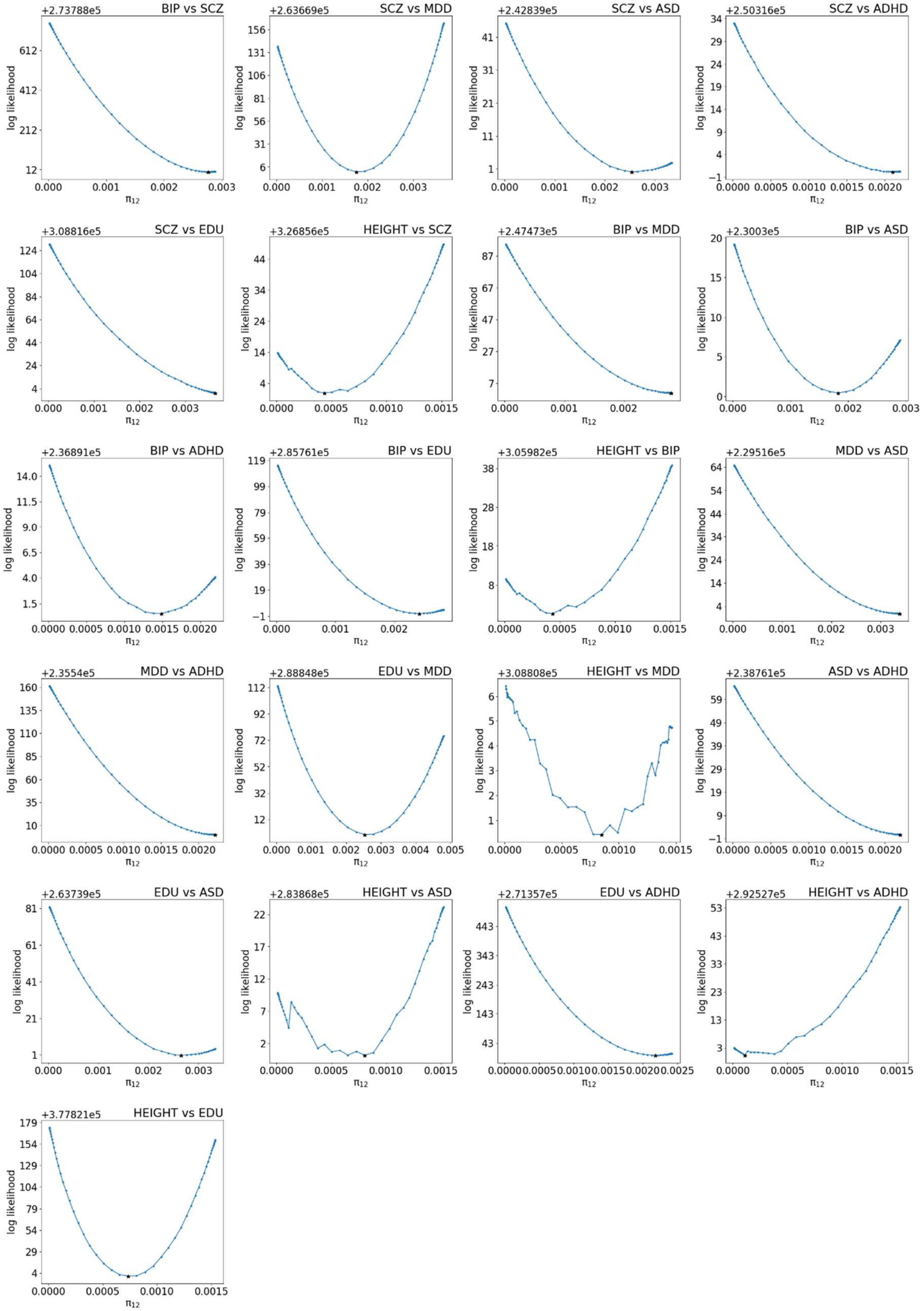

Log-likelihood of the bivariate fit as a function of π_{12} parameter. The remaining parameters of the model were constrained to their fitted values. Asterix indicates fitted value of π_{12} parameter.

Supplementary Figure 20. Comparison of the LD structure between 1000 Genomes EUR population and the simulated Hapgen2 panel

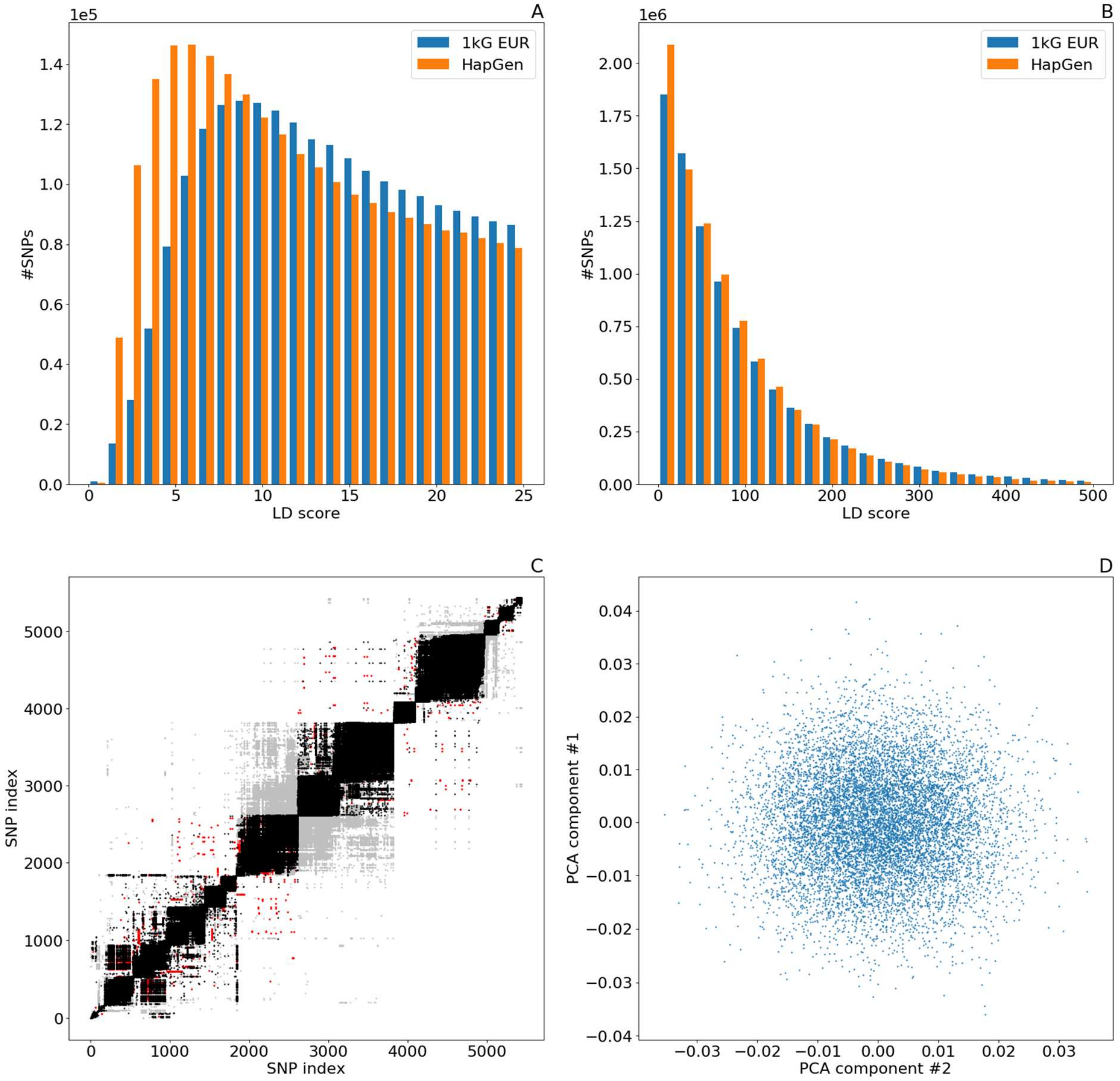

Figure (A) shows the histogram of LD scores, restricted to small values of LD (up to 25) to highlight differences between 1kG and Hapgen2 panels. Figure (B) shows the entire range of LD scores, highlighting the overall consistency in the tails of the LD score distributions. Figure (C) shows sparsity patterns of the LD structure for the initial 2000 KB region on the chromosome 1, containing 5439 SNPs. Points in black indicate SNPs with consistent LD r2 across the two panels (LD r2 correlation between SNPs exceeds a fixed threshold of LD r2=0.2 in both 1kG and Hapgen2); points in grey indicate SNPs that are in high LD in 1kG panel but not in Hapgen2 panel; points in red indicate SNPs that are in LD in Hapgen2 panel but not in 1kG panel. The large area of SNPs in grey color, with indices from ca. 2000 to 4000, is because Hapgen2 panel was simulated in chunks of ca. 2900 SNPs, breaking through the original LD blocks in 1kG panel, however it has reasonably small effect on the LD histograms (A) and (B). Figure (D) shows the first and the second PCA components of the random 10K subjects in Hapgen2 panel.

Supplementary Table 1. Simulations: parameter estimates in bivariate analysis

Simulations wtih synthetic data following assumptions of the MiXeR model. Columns: true_pi1u, true_pi2u – simulated total polygenicity of trait1 and trait2; true_pi12 – simulated polygenicity of the shared component; true_h2 – simulated heritability; true_rg - simulated value of rho12 parameter (correlation of effect sizes within shared polygenic component); spow – value of "S" parameter that describes MAF-dependent architecture (with S=-1 corresponding to LDSR MAF model, S=0 corresponding to MiXeR MAF model); pi12 – estimates of shared polygenicity, followed by standard errors (the first number in parenthesis is the mean of theoretical standard errors derived from variance formulas; the second one is the empirical standard errors across 10 simulation runs); rg – estimates of genetic correlation, rho12 – estimates of correlation of effect sizes in shared component.

true_pi1u	true_pi2u	true_pi12	true_h2	true_rg	spow	pi12	rg	rho12
3.00E-03	3.00E-03	0.00E+00	0.1	0	0	8.96e-04 (6.44e-04 / 9.43e-04)	-0.002 (0.079 / 0.059)	0.046 (0.309 / 0.378)
3.00E-03	3.00E-03	0.00E+00	0.1	0.5	0	9.04e-04 (7.52e-04 / 5.82e-04)	-0.009 (0.070 / 0.035)	-0.088 (0.244 / 0.251)
3.00E-03	3.00E-03	0.00E+00	0.4	0	0	1.00e-04 (8.62e-05 / 5.87e-05)	0.008 (0.018 / 0.014)	0.167 (0.348 / 0.386)
3.00E-03	3.00E-03	0.00E+00	0.4	0.5	0	1.19e-04 (1.28e-04 / 1.08e-04)	-0.002 (0.028 / 0.023)	-0.057 (0.326 / 0.419)
3.00E-03	3.00E-03	0.00E+00	0.7	0	0	1.09e-04 (8.55e-05 / 4.24e-05)	-0.002 (0.020 / 0.022)	0.037 (0.289 / 0.598)
3.00E-03	3.00E-03	0.00E+00	0.7	0.5	0	8.00e-05 (1.12e-04 / 4.47e-05)	0.010 (0.029 / 0.012)	0.325 (0.294 / 0.458)
3.00E-03	3.00E-03	1.20E-03	0.1	0	0	1.53e-03 (8.75e-04 / 2.00e-03)	-0.030 (0.050 / 0.037)	-0.195 (0.186 / 0.276)
3.00E-03	3.00E-03	1.20E-03	0.1	0.5	0	1.22e-03 (5.45e-04 / 5.40e-04)	0.201 (0.097 / 0.047)	0.607 (0.279 / 0.239)
3.00E-03	3.00E-03	1.20E-03	0.4	0	0	1.09e-03 (1.79e-04 / 3.17e-04)	-0.000 (0.015 / 0.021)	0.004 (0.047 / 0.055)
3.00E-03	3.00E-03	1.20E-03	0.4	0.5	0	1.18e-03 (2.04e-04 / 3.00e-04)	0.211 (0.037 / 0.017)	0.549 (0.136 / 0.176)
3.00E-03	3.00E-03	1.20E-03	0.7	0	0	1.23e-03 (1.04e-04 / 1.44e-04)	0.000 (0.011 / 0.015)	0.001 (0.027 / 0.035)
3.00E-03	3.00E-03	1.20E-03	0.7	0.5	0	1.19e-03 (1.11e-04 / 2.01e-04)	0.209 (0.014 / 0.018)	0.546 (0.055 / 0.137)
3.00E-03	3.00E-03	1.80E-03	0.1	0	0	1.32e-03 (7.17e-04 / 6.13e-04)	0.017 (0.054 / 0.040)	0.028 (0.130 / 0.100)
3.00E-03	3.00E-03	1.80E-03	0.1	0.5	0	1.84e-03 (6.44e-04 / 8.82e-04)	0.296 (0.077 / 0.035)	0.566 (0.095 / 0.186)
3.00E-03	3.00E-03	1.80E-03	0.4	0	0	1.91e-03 (2.01e-04 / 2.33e-04)	0.000 (0.016 / 0.019)	0.001 (0.026 / 0.033)
3.00E-03	3.00E-03	1.80E-03	0.4	0.5	0	1.66e-03 (2.27e-04 / 3.10e-04)	0.298 (0.029 / 0.020)	0.566 (0.089 / 0.166)
3.00E-03	3.00E-03	1.80E-03	0.7	0	0	1.83e-03 (1.20e-04 / 2.11e-04)	0.002 (0.011 / 0.021)	0.004 (0.019 / 0.035)
3.00E-03	3.00E-03	1.80E-03	0.7	0.5	0	1.79e-03 (1.28e-04 / 2.20e-04)	0.298 (0.014 / 0.011)	0.506 (0.032 / 0.059)
3.00E-03	3.00E-03	2.40E-03	0.1	0	0	1.98e-03 (1.05e-03 / 1.06e-03)	-0.016 (0.058 / 0.047)	-0.042 (0.133 / 0.115)
3.00E-03	3.00E-03	2.40E-03	0.1	0.5	0	1.92e-03 (6.88e-04 / 5.35e-04)	0.415 (0.147 / 0.049)	0.679 (0.219 / 0.140)
3.00E-03	3.00E-03	2.40E-03	0.4	0	0	2.22e-03 (2.25e-04 / 1.96e-04)	0.003 (0.015 / 0.018)	0.003 (0.021 / 0.024)
3.00E-03	3.00E-03	2.40E-03	0.4	0.5	0	2.36e-03 (2.07e-04 / 4.89e-04)	0.403 (0.027 / 0.025)	0.523 (0.036 / 0.117)
3.00E-03	3.00E-03	2.40E-03	0.7	0	0	2.32e-03 (1.34e-04 / 1.45e-04)	-0.002 (0.011 / 0.013)	-0.003 (0.014 / 0.016)
3.00E-03	3.00E-03	2.40E-03	0.7	0.5	0	2.35e-03 (1.49e-04 / 2.69e-04)	0.398 (0.016 / 0.013)	0.502 (0.022 / 0.037)
3.00E-03	3.00E-03	3.00E-03	0.1	0	0	2.32e-03 (1.04e-03 / 8.94e-04)	0.007 (0.044 / 0.054)	0.011 (0.078 / 0.072)
3.00E-03	3.00E-03	3.00E-03	0.1	0.5	0	2.11e-03 (7.55e-04 / 5.00e-04)	0.492 (0.211 / 0.034)	0.790 (0.363 / 0.145)
3.00E-03	3.00E-03	3.00E-03	0.4	0	0	2.71e-03 (1.76e-04 / 1.27e-04)	-0.000 (0.013 / 0.024)	-0.000 (0.015 / 0.027)
3.00E-03	3.00E-03	3.00E-03	0.4	0.5	0	2.82e-03 (2.01e-04 / 1.93e-04)	0.500 (0.027 / 0.019)	0.541 (0.012 / 0.039)
3.00E-03	3.00E-03	3.00E-03	0.7	0	0	2.88e-03 (1.25e-04 / 1.23e-04)	-0.000 (0.010 / 0.024)	-0.000 (0.011 / 0.025)
3.00E-03	3.00E-03	3.00E-03	0.7	0.5	0	2.83e-03 (1.64e-04 / 1.29e-04)	0.500 (0.023 / 0.014)	0.523 (0.008 / 0.025)
3.00E-03	3.00E-03	6.00E-04	0.1	0	0	1.03e-03 (5.60e-04 / 7.98e-04)	0.007 (0.058 / 0.070)	0.029 (0.193 / 0.375)
3.00E-03	3.00E-03	6.00E-04	0.1	0.5	0	1.16e-03 (6.28e-04 / 7.77e-04)	0.093 (0.079 / 0.046)	0.320 (0.242 / 0.222)
3.00E-03	3.00E-03	6.00E-04	0.4	0	0	5.44e-04 (1.85e-04 / 2.98e-04)	0.008 (0.022 / 0.026)	0.138 (0.115 / 0.259)
3.00E-03	3.00E-03	6.00E-04	0.4	0.5	0	6.03e-04 (1.95e-04 / 2.08e-04)	0.103 (0.042 / 0.020)	0.555 (0.197 / 0.156)
3.00E-03	3.00E-03	6.00E-04	0.7	0	0	6.54e-04 (1.03e-04 / 1.36e-04)	-0.001 (0.011 / 0.013)	-0.006 (0.051 / 0.065)
3.00E-03	3.00E-03	6.00E-04	0.7	0.5	0	5.43e-04 (9.26e-05 / 1.60e-04)	0.101 (0.014 / 0.017)	0.584 (0.098 / 0.159)
3.00E-03	3.00E-04	0.00E+00	0.1	0	0	9.61e-05 (6.18e-05 / 3.92e-05)	0.018 (0.051 / 0.040)	0.168 (0.354 / 0.383)
3.00E-03	3.00E-04	0.00E+00	0.1	0.5	0	7.58e-05 (4.94e-05 / 4.92e-05)	-0.025 (0.043 / 0.025)	-0.329 (0.432 / 0.311)
3.00E-03	3.00E-04	0.00E+00	0.4	0	0	3.49e-05 (1.73e-05 / 2.15e-05)	0.003 (0.012 / 0.023)	-0.037 (0.252 / 0.558)
3.00E-03	3.00E-04	0.00E+00	0.4	0.5	0	3.14e-05 (2.18e-05 / 1.41e-05)	0.010 (0.022 / 0.024)	0.240 (0.331 / 0.585)
3.00E-03	3.00E-04	0.00E+00	0.7	0	0	1.99e-05 (1.26e-05 / 1.03e-05)	0.001 (0.012 / 0.015)	-0.027 (0.284 / 0.726)
3.00E-03	3.00E-04	0.00E+00	0.7	0.5	0	2.68e-05 (1.43e-05 / 1.11e-05)	0.003 (0.011 / 0.013)	0.140 (0.273 / 0.450)
3.00E-03	3.00E-04	1.20E-04	0.1	0	0	1.70e-04 (6.97e-05 / 9.43e-05)	0.008 (0.033 / 0.033)	0.027 (0.209 / 0.249)
3.00E-03	3.00E-04	1.20E-04	0.1	0.5	0	2.00e-04 (6.52e-05 / 9.85e-05)	0.056 (0.040 / 0.052)	0.332 (0.211 / 0.289)
3.00E-03	3.00E-04	1.20E-04	0.4	0	0	1.29e-04 (2.78e-05 / 3.53e-05)	-0.003 (0.011 / 0.015)	-0.025 (0.088 / 0.144)
3.00E-03	3.00E-04	1.20E-04	0.4	0.5	0	1.52e-04 (2.42e-05 / 4.51e-05)	0.063 (0.013 / 0.024)	0.415 (0.089 / 0.193)
3.00E-03	3.00E-04	1.20E-04	0.7	0	0	1.16e-04 (1.88e-05 / 2.49e-05)	0.004 (0.009 / 0.013)	0.032 (0.074 / 0.122)
3.00E-03	3.00E-04	1.20E-04	0.7	0.5	0	1.21e-04 (2.41e-05 / 2.17e-05)	0.060 (0.019 / 0.019)	0.494 (0.175 / 0.217)

Supplementary Table 1 (continuation)

true_pi1u	true_pi2u	true_pi12	true_h2	true_rg	spow	pi12	rg	rho12
3.00E-03	3.00E-04	1.80E-04	0.1	0	0	1.68e-04 (8.31e-05 / 6.95e-05)	0.004 (0.044 / 0.040)	0.020 (0.248 / 0.273)
3.00E-03	3.00E-04	1.80E-04	0.1	0.5	0	2.26e-04 (6.90e-05 / 5.08e-05)	0.105 (0.043 / 0.068)	0.456 (0.168 / 0.264)
3.00E-03	3.00E-04	1.80E-04	0.4	0	0	1.47e-04 (3.79e-05 / 4.45e-05)	0.002 (0.015 / 0.024)	0.001 (0.101 / 0.134)
3.00E-03	3.00E-04	1.80E-04	0.4	0.5	0	2.00e-04 (4.55e-05 / 4.73e-05)	0.089 (0.023 / 0.026)	0.438 (0.125 / 0.163)
3.00E-03	3.00E-04	1.80E-04	0.7	0	0	1.89e-04 (2.01e-05 / 4.43e-05)	0.004 (0.009 / 0.017)	0.023 (0.047 / 0.095)
3.00E-03	3.00E-04	1.80E-04	0.7	0.5	0	1.88e-04 (2.31e-05 / 4.18e-05)	0.103 (0.011 / 0.019)	0.537 (0.069 / 0.114)
3.00E-03	3.00E-04	2.40E-04	0.1	0	0	2.48e-04 (9.64e-05 / 4.23e-05)	-0.028 (0.035 / 0.043)	-0.086 (0.151 / 0.167)
3.00E-03	3.00E-04	2.40E-04	0.1	0.5	0	2.12e-04 (6.26e-05 / 7.56e-05)	0.116 (0.046 / 0.033)	0.630 (0.150 / 0.231)
3.00E-03	3.00E-04	2.40E-04	0.4	0	0	2.31e-04 (3.53e-05 / 3.31e-05)	-0.003 (0.011 / 0.022)	-0.017 (0.047 / 0.085)
3.00E-03	3.00E-04	2.40E-04	0.4	0.5	0	2.34e-04 (3.69e-05 / 4.72e-05)	0.130 (0.024 / 0.025)	0.557 (0.096 / 0.196)
3.00E-03	3.00E-04	2.40E-04	0.7	0	0	2.22e-04 (2.96e-05 / 2.20e-05)	-0.002 (0.009 / 0.013)	-0.010 (0.038 / 0.058)
3.00E-03	3.00E-04	2.40E-04	0.7	0.5	0	2.32e-04 (1.83e-05 / 4.31e-05)	0.130 (0.010 / 0.019)	0.548 (0.047 / 0.097)
3.00E-03	3.00E-04	3.00E-04	0.1	0	0	2.46e-04 (1.00e-04 / 7.69e-05)	-0.020 (0.032 / 0.048)	-0.041 (0.133 / 0.304)
3.00E-03	3.00E-04	3.00E-04	0.1	0.5	0	2.44e-04 (5.94e-05 / 4.83e-05)	0.162 (0.061 / 0.064)	0.650 (0.234 / 0.222)
3.00E-03	3.00E-04	3.00E-04	0.4	0	0	2.63e-04 (2.10e-05 / 3.29e-05)	0.004 (0.011 / 0.031)	0.018 (0.040 / 0.112)
3.00E-03	3.00E-04	3.00E-04	0.4	0.5	0	2.75e-04 (2.17e-05 / 3.41e-05)	0.168 (0.016 / 0.017)	0.584 (0.038 / 0.088)
3.00E-03	3.00E-04	3.00E-04	0.7	0	0	2.81e-04 (1.49e-05 / 2.01e-05)	0.002 (0.009 / 0.013)	0.006 (0.030 / 0.043)
3.00E-03	3.00E-04	3.00E-04	0.7	0.5	0	2.77e-04 (3.25e-05 / 1.43e-05)	0.154 (0.018 / 0.013)	0.518 (0.034 / 0.047)
3.00E-03	3.00E-04	6.00E-05	0.1	0	0	1.16e-04 (5.82e-05 / 3.94e-05)	0.030 (0.036 / 0.039)	0.242 (0.200 / 0.381)
3.00E-03	3.00E-04	6.00E-05	0.1	0.5	0	1.74e-04 (5.79e-05 / 6.74e-05)	0.057 (0.039 / 0.066)	0.338 (0.139 / 0.458)
3.00E-03	3.00E-04	6.00E-05	0.4	0	0	6.30e-05 (2.54e-05 / 2.92e-05)	0.011 (0.013 / 0.018)	0.178 (0.167 / 0.339)
3.00E-03	3.00E-04	6.00E-05	0.4	0.5	0	7.83e-05 (2.49e-05 / 3.47e-05)	0.029 (0.013 / 0.030)	0.348 (0.144 / 0.322)
3.00E-03	3.00E-04	6.00E-05	0.7	0	0	5.33e-05 (2.21e-05 / 2.02e-05)	-0.002 (0.009 / 0.012)	-0.034 (0.130 / 0.281)
3.00E-03	3.00E-04	6.00E-05	0.7	0.5	0	6.47e-05 (2.62e-05 / 2.27e-05)	0.028 (0.021 / 0.016)	0.463 (0.235 / 0.287)
3.00E-04	3.00E-04	0.00E+00	0.1	0	0	2.42e-05 (8.12e-06 / 1.32e-05)	0.033 (0.022 / 0.068)	0.277 (0.086 / 0.682)
3.00E-04	3.00E-04	0.00E+00	0.1	0.5	0	2.17e-05 (1.34e-05 / 1.22e-05)	-0.015 (0.036 / 0.047)	-0.205 (0.257 / 0.538)
3.00E-04	3.00E-04	0.00E+00	0.4	0	0	8.80e-06 (5.84e-06 / 8.07e-06)	-0.002 (0.017 / 0.030)	-0.031 (0.399 / 0.597)
3.00E-04	3.00E-04	0.00E+00	0.4	0.5	0	6.63e-06 (8.54e-06 / 5.12e-06)	0.009 (0.016 / 0.018)	0.336 (0.183 / 0.704)
3.00E-04	3.00E-04	0.00E+00	0.7	0	0	5.83e-06 (3.76e-06 / 3.21e-06)	0.005 (0.010 / 0.014)	0.166 (0.223 / 0.660)
3.00E-04	3.00E-04	0.00E+00	0.7	0.5	0	6.73e-06 (2.65e-06 / 2.79e-06)	0.011 (0.013 / 0.014)	0.410 (0.340 / 0.633)
3.00E-04	3.00E-04	1.20E-04	0.1	0	0	1.22e-04 (2.68e-05 / 3.06e-05)	0.013 (0.029 / 0.036)	0.038 (0.075 / 0.088)
3.00E-04	3.00E-04	1.20E-04	0.1	0.5	0	1.11e-04 (2.87e-05 / 3.25e-05)	0.164 (0.054 / 0.054)	0.485 (0.156 / 0.174)
3.00E-04	3.00E-04	1.20E-04	0.4	0	0	1.22e-04 (9.50e-06 / 9.56e-06)	-0.002 (0.014 / 0.023)	-0.006 (0.037 / 0.056)
3.00E-04	3.00E-04	1.20E-04	0.4	0.5	0	1.16e-04 (9.21e-06 / 1.44e-05)	0.196 (0.016 / 0.026)	0.499 (0.041 / 0.066)
3.00E-04	3.00E-04	1.20E-04	0.7	0	0	1.21e-04 (7.19e-06 / 9.17e-06)	-0.008 (0.013 / 0.034)	-0.018 (0.032 / 0.086)
3.00E-04	3.00E-04	1.20E-04	0.7	0.5	0	1.23e-04 (6.92e-06 / 1.47e-05)	0.199 (0.013 / 0.020)	0.496 (0.031 / 0.058)
3.00E-04	3.00E-04	1.80E-04	0.1	0	0	1.61e-04 (2.85e-05 / 3.35e-05)	0.018 (0.029 / 0.036)	0.039 (0.057 / 0.074)
3.00E-04	3.00E-04	1.80E-04	0.1	0.5	0	1.50e-04 (2.96e-05 / 4.80e-05)	0.299 (0.051 / 0.075)	0.592 (0.102 / 0.152)
3.00E-04	3.00E-04	1.80E-04	0.4	0	0	1.74e-04 (1.15e-05 / 1.24e-05)	-0.004 (0.017 / 0.030)	-0.006 (0.028 / 0.050)
3.00E-04	3.00E-04	1.80E-04	0.4	0.5	0	1.73e-04 (1.11e-05 / 9.43e-06)	0.274 (0.018 / 0.019)	0.463 (0.028 / 0.034)
3.00E-04	3.00E-04	1.80E-04	0.7	0	0	1.77e-04 (8.78e-06 / 1.58e-05)	0.006 (0.015 / 0.033)	0.010 (0.025 / 0.054)
3.00E-04	3.00E-04	1.80E-04	0.7	0.5	0	1.79e-04 (8.46e-06 / 1.72e-05)	0.309 (0.015 / 0.022)	0.513 (0.022 / 0.033)
3.00E-04	3.00E-04	2.40E-04	0.1	0	0	2.49e-04 (4.03e-05 / 4.40e-05)	0.004 (0.030 / 0.056)	0.005 (0.041 / 0.076)
3.00E-04	3.00E-04	2.40E-04	0.1	0.5	0	2.32e-04 (3.77e-05 / 3.70e-05)	0.381 (0.047 / 0.065)	0.499 (0.053 / 0.091)
3.00E-04	3.00E-04	2.40E-04	0.4	0	0	2.41e-04 (1.54e-05 / 1.94e-05)	0.005 (0.018 / 0.032)	0.005 (0.023 / 0.041)
3.00E-04	3.00E-04	2.40E-04	0.4	0.5	0	2.48e-04 (1.53e-05 / 1.83e-05)	0.404 (0.021 / 0.035)	0.499 (0.023 / 0.047)
3.00E-04	3.00E-04	2.40E-04	0.7	0	0	2.51e-04 (1.31e-05 / 1.90e-05)	0.002 (0.016 / 0.027)	0.002 (0.020 / 0.034)
3.00E-04	3.00E-04	2.40E-04	0.7	0.5	0	2.35e-04 (1.02e-05 / 9.97e-06)	0.394 (0.016 / 0.038)	0.502 (0.018 / 0.038)
3.00E-04	3.00E-04	3.00E-04	0.1	0	0	2.45e-04 (6.03e-05 / 3.57e-05)	0.033 (0.037 / 0.073)	0.038 (0.038 / 0.086)
3.00E-04	3.00E-04	3.00E-04	0.1	0.5	0	2.44e-04 (5.52e-05 / 3.05e-05)	0.483 (0.097 / 0.034)	0.587 (0.039 / 0.061)
3.00E-04	3.00E-04	3.00E-04	0.4	0	0	3.03e-04 (1.44e-05 / 1.59e-05)	0.008 (0.019 / 0.058)	0.009 (0.019 / 0.061)
3.00E-04	3.00E-04	3.00E-04	0.4	0.5	0	2.86e-04 (2.55e-05 / 1.23e-05)	0.512 (0.042 / 0.036)	0.532 (0.015 / 0.044)
3.00E-04	3.00E-04	3.00E-04	0.7	0	0	2.91e-04 (1.07e-05 / 8.90e-06)	-0.010 (0.017 / 0.025)	-0.010 (0.018 / 0.026)
3.00E-04	3.00E-04	3.00E-04	0.7	0.5	0	2.82e-04 (1.59e-05 / 1.55e-05)	0.514 (0.028 / 0.029)	0.539 (0.012 / 0.041)
3.00E-04	3.00E-04	6.00E-05	0.1	0	0	5.24e-05 (1.74e-05 / 1.86e-05)	-0.011 (0.031 / 0.048)	-0.050 (0.175 / 0.303)
3.00E-04	3.00E-04	6.00E-05	0.1	0.5	0	7.23e-05 (2.53e-05 / 3.19e-05)	0.110 (0.045 / 0.046)	0.531 (0.160 / 0.224)
3.00E-04	3.00E-04	6.00E-05	0.4	0	0	5.41e-05 (6.93e-06 / 9.61e-06)	-0.002 (0.012 / 0.021)	-0.017 (0.065 / 0.109)
3.00E-04	3.00E-04	6.00E-05	0.4	0.5	0	6.04e-05 (7.14e-06 / 1.12e-05)	0.106 (0.013 / 0.016)	0.533 (0.073 / 0.091)
3.00E-04	3.00E-04	6.00E-05	0.7	0	0	6.12e-05 (5.26e-06 / 6.99e-06)	0.002 (0.010 / 0.024)	0.011 (0.051 / 0.115)
3.00E-04	3.00E-04	6.00E-05	0.7	0.5	0	6.13e-05 (5.25e-06 / 9.92e-06)	0.098 (0.010 / 0.016)	0.493 (0.051 / 0.070)

Supplementary Table 2. Simulations: polygenicity and heritability estimates in univariate analysis

Univariate simulations with synthetic data. Columns: true_pi1u – simulated polygenicity; true_h2 – simulated heritability; spow – value of "S" parameter that describes MAF-dependent architecture (S=-1 corresponds to LDSR model, S=0 corresponds to MiXeR model); pi – estimates of polygenicity, followed by standard errors (the first number in parenthesis is the mean of theoretical standard errors derived from variance formulas; the second one is the empirical standard errors across 120 simulation runs); h2 – estimates of heritability.

true_pi1u	true_h2	spow	pi_vec	h2
0.003	0.1	0	3.63e-03 (4.58e-03 / 2.94e-03)	0.099 (0.007 / 0.008)
0.003	0.4	0	2.99e-03 (2.20e-04 / 2.51e-04)	0.399 (0.011 / 0.010)
0.003	0.7	0	2.99e-03 (1.47e-04 / 1.71e-04)	0.699 (0.014 / 0.012)
0.0003	0.1	0	2.92e-04 (4.11e-05 / 4.50e-05)	0.097 (0.007 / 0.007)
0.0003	0.4	0	3.03e-04 (1.66e-05 / 2.18e-05)	0.403 (0.013 / 0.013)
0.0003	0.7	0	3.03e-04 (1.26e-05 / 1.75e-05)	0.704 (0.020 / 0.017)
0.003	0.4	-0.25	2.93e-03 (2.28e-04 / 2.40e-04)	0.376 (0.011 / 0.011)
0.003	0.4	-0.5	2.79e-03 (2.36e-04 / 2.70e-04)	0.343 (0.010 / 0.010)
0.003	0.4	-0.75	2.54e-03 (2.43e-04 / 2.83e-04)	0.298 (0.010 / 0.010)
0.0003	0.4	-0.25	2.89e-04 (1.63e-05 / 2.09e-05)	0.393 (0.013 / 0.011)
0.0003	0.4	-0.5	2.67e-04 (1.58e-05 / 2.28e-05)	0.379 (0.013 / 0.013)
0.0003	0.4	-0.75	2.24e-04 (1.44e-05 / 1.98e-05)	0.358 (0.013 / 0.013)

Supplementary Table 3. Sensitivity analysis: polygenic overlap estimates under differential genomic enrichment

Simulations with model misspecification showing bias in bivariate estimates in the presence of genomic annotations. The data were simulated independently in the two traits, but with shared pattern of enrichment. Columns: true_N1u – total number of simulated causal variants (in 1,000) per trait; true_n12 – number of shared causal variants, arising by chance due to high polygenicity; true_h2 – simulated heritability; N1u - estimated total number of causal variants in trait1, in 1,000; N12 – estimated number of shared causal variants, in 1,000; N12/N1u – fraction of shared causal variants with respect to total polygenicity of the first trait; h2 – estimated heritability.

true_N1u (x1000)	true_h2	true_n12	N1u (x1000)	N12 (x1000)	N12/N1u	h2
30	0.1	0.41 (0.02)	23.71 (8.07/8.74)	4.86 (4.40/4.83)	0.22 (0.19/0.24)	0.09 (0.01/0.01)
30	0.4	0.41 (0.02)	23.37 (1.65/2.09)	1.83 (1.41/1.24)	0.08 (0.06/0.05)	0.37 (0.01/0.01)
30	0.7	0.41 (0.02)	24.19 (1.17/1.09)	1.90 (1.12/0.82)	0.08 (0.05/0.03)	0.66 (0.01/0.01)
3	0.1	0.00 (0.00)	2.46 (0.36/0.35)	0.05 (0.34/0.05)	0.02 (0.14/0.02)	0.09 (0.01/0.01)
3	0.4	0.00 (0.00)	2.70 (0.15/0.17)	0.04 (0.18/0.03)	0.01 (0.06/0.01)	0.40 (0.01/0.01)
3	0.7	0.00 (0.00)	2.69 (0.11/0.13)	0.05 (0.13/0.04)	0.02 (0.05/0.02)	0.71 (0.02/0.02)

Supplementary Table 4. Sensitivity analysis: parameter estimates in bivariate analysis under mis-specified MAF architecture

Simulations with model misspecification showing bias in bivariate estimates under mis-specified MAF-dependent architecture. Appearance of the columns is as in Supplementary Table 1.

true_pi1u	true_pi2u	true_pi12	true_h2	true_rg	spow	pi12	rg	rho12
3.00E-03	3.00E-03	0.00E+00	0.4	0	-0.25	1.13e-04 (9.69e-05 / 6.38e-05)	-0.002 (0.030 / 0.028)	-0.095 (0.361 / 0.622)
3.00E-03	3.00E-03	0.00E+00	0.4	0	-0.5	1.56e-04 (2.16e-04 / 9.40e-05)	0.007 (0.028 / 0.017)	0.108 (0.271 / 0.434)
3.00E-03	3.00E-03	0.00E+00	0.4	0	-0.75	1.16e-04 (8.67e-05 / 6.06e-05)	0.002 (0.025 / 0.018)	0.030 (0.374 / 0.407)
3.00E-03	3.00E-03	0.00E+00	0.4	0.5	-0.25	1.27e-04 (5.60e-05 / 8.44e-05)	0.004 (0.016 / 0.021)	0.070 (0.214 / 0.525)
3.00E-03	3.00E-03	0.00E+00	0.4	0.5	-0.5	1.61e-04 (1.87e-04 / 9.01e-05)	-0.001 (0.049 / 0.027)	-0.174 (0.407 / 0.480)
3.00E-03	3.00E-03	0.00E+00	0.4	0.5	-0.75	2.07e-04 (3.00e-04 / 5.90e-05)	0.003 (0.039 / 0.036)	0.041 (0.219 / 0.423)
3.00E-03	3.00E-03	1.20E-03	0.4	0	-0.25	1.09e-03 (1.87e-04 / 3.13e-04)	0.005 (0.016 / 0.021)	0.006 (0.050 / 0.065)
3.00E-03	3.00E-03	1.20E-03	0.4	0	-0.5	1.05e-03 (1.75e-04 / 1.65e-04)	0.013 (0.017 / 0.020)	0.031 (0.047 / 0.050)
3.00E-03	3.00E-03	1.20E-03	0.4	0	-0.75	9.69e-04 (2.03e-04 / 3.07e-04)	0.011 (0.020 / 0.024)	0.035 (0.064 / 0.087)
3.00E-03	3.00E-03	1.20E-03	0.4	0.5	-0.25	1.08e-03 (1.60e-04 / 3.25e-04)	0.190 (0.025 / 0.024)	0.563 (0.108 / 0.178)
3.00E-03	3.00E-03	1.20E-03	0.4	0.5	-0.5	1.17e-03 (1.72e-04 / 1.95e-04)	0.196 (0.021 / 0.031)	0.461 (0.068 / 0.120)
3.00E-03	3.00E-03	1.20E-03	0.4	0.5	-0.75	8.97e-04 (1.39e-04 / 2.82e-04)	0.206 (0.027 / 0.019)	0.610 (0.094 / 0.181)
3.00E-03	3.00E-03	1.80E-03	0.4	0	-0.25	1.92e-03 (2.14e-04 / 2.88e-04)	0.001 (0.016 / 0.024)	0.003 (0.027 / 0.040)
3.00E-03	3.00E-03	1.80E-03	0.4	0	-0.5	1.67e-03 (2.10e-04 / 2.05e-04)	0.007 (0.018 / 0.022)	0.010 (0.031 / 0.038)
3.00E-03	3.00E-03	1.80E-03	0.4	0	-0.75	1.67e-03 (2.28e-04 / 3.82e-04)	0.000 (0.019 / 0.011)	0.003 (0.032 / 0.024)
3.00E-03	3.00E-03	1.80E-03	0.4	0.5	-0.25	1.51e-03 (2.26e-04 / 3.45e-04)	0.302 (0.029 / 0.030)	0.619 (0.090 / 0.136)
3.00E-03	3.00E-03	1.80E-03	0.4	0.5	-0.5	1.76e-03 (3.54e-04 / 4.25e-04)	0.305 (0.072 / 0.022)	0.520 (0.155 / 0.138)
3.00E-03	3.00E-03	1.80E-03	0.4	0.5	-0.75	1.43e-03 (2.78e-04 / 4.12e-04)	0.302 (0.047 / 0.025)	0.612 (0.122 / 0.221)
3.00E-03	3.00E-03	2.40E-03	0.4	0	-0.25	2.40e-03 (2.50e-04 / 3.31e-04)	-0.006 (0.016 / 0.019)	-0.006 (0.020 / 0.024)
3.00E-03	3.00E-03	2.40E-03	0.4	0	-0.5	2.12e-03 (2.54e-04 / 2.22e-04)	-0.004 (0.017 / 0.018)	-0.006 (0.022 / 0.023)
3.00E-03	3.00E-03	2.40E-03	0.4	0	-0.75	1.84e-03 (1.97e-04 / 4.34e-04)	0.001 (0.018 / 0.014)	0.004 (0.027 / 0.024)
3.00E-03	3.00E-03	2.40E-03	0.4	0.5	-0.25	2.09e-03 (2.29e-04 / 4.55e-04)	0.408 (0.034 / 0.029)	0.580 (0.055 / 0.119)
3.00E-03	3.00E-03	2.40E-03	0.4	0.5	-0.5	2.19e-03 (2.14e-04 / 4.11e-04)	0.417 (0.053 / 0.028)	0.556 (0.090 / 0.159)
3.00E-03	3.00E-03	2.40E-03	0.4	0.5	-0.75	1.89e-03 (2.63e-04 / 3.45e-04)	0.404 (0.039 / 0.022)	0.526 (0.056 / 0.092)
3.00E-03	3.00E-03	3.00E-03	0.4	0	-0.25	2.59e-03 (1.71e-04 / 1.67e-04)	0.010 (0.014 / 0.024)	0.011 (0.015 / 0.027)
3.00E-03	3.00E-03	3.00E-03	0.4	0	-0.5	2.64e-03 (2.15e-04 / 2.64e-04)	-0.006 (0.015 / 0.022)	-0.007 (0.017 / 0.025)
3.00E-03	3.00E-03	3.00E-03	0.4	0	-0.75	2.25e-03 (1.74e-04 / 1.41e-04)	-0.012 (0.016 / 0.021)	-0.014 (0.018 / 0.022)
3.00E-03	3.00E-03	3.00E-03	0.4	0.5	-0.25	2.73e-03 (2.07e-04 / 1.18e-04)	0.510 (0.029 / 0.032)	0.555 (0.013 / 0.035)
3.00E-03	3.00E-03	3.00E-03	0.4	0.5	-0.5	2.48e-03 (2.25e-04 / 2.31e-04)	0.500 (0.032 / 0.025)	0.565 (0.030 / 0.066)
3.00E-03	3.00E-03	3.00E-03	0.4	0.5	-0.75	2.01e-03 (3.01e-04 / 2.92e-04)	0.505 (0.058 / 0.017)	0.624 (0.044 / 0.116)
3.00E-03	3.00E-03	6.00E-04	0.4	0	-0.25	5.04e-04 (2.01e-04 / 1.27e-04)	0.001 (0.020 / 0.020)	0.008 (0.112 / 0.113)
3.00E-03	3.00E-03	6.00E-04	0.4	0	-0.5	7.22e-04 (1.86e-04 / 2.09e-04)	0.004 (0.019 / 0.017)	0.021 (0.075 / 0.070)
3.00E-03	3.00E-03	6.00E-04	0.4	0	-0.75	5.06e-04 (2.02e-04 / 1.37e-04)	0.004 (0.022 / 0.009)	0.019 (0.103 / 0.048)
3.00E-03	3.00E-03	6.00E-04	0.4	0.5	-0.25	6.01e-04 (1.21e-04 / 1.93e-04)	0.110 (0.020 / 0.015)	0.577 (0.108 / 0.190)
3.00E-03	3.00E-03	6.00E-04	0.4	0.5	-0.5	7.17e-04 (1.63e-04 / 2.51e-04)	0.090 (0.019 / 0.020)	0.389 (0.101 / 0.141)
3.00E-03	3.00E-03	6.00E-04	0.4	0.5	-0.75	6.88e-04 (2.59e-04 / 2.52e-04)	0.096 (0.045 / 0.034)	0.415 (0.192 / 0.175)
3.00E-04	3.00E-04	0.00E+00	0.4	0	-0.25	6.08e-06 (4.84e-06 / 2.96e-06)	-0.008 (0.016 / 0.017)	-0.251 (0.320 / 0.650)
3.00E-04	3.00E-04	0.00E+00	0.4	0	-0.5	6.85e-06 (1.07e-05 / 2.78e-06)	-0.009 (0.037 / 0.017)	-0.367 (0.340 / 0.542)
3.00E-04	3.00E-04	0.00E+00	0.4	0	-0.75	6.66e-06 (8.31e-06 / 3.96e-06)	-0.004 (0.018 / 0.023)	-0.079 (0.271 / 0.455)
3.00E-04	3.00E-04	0.00E+00	0.4	0.5	-0.25	6.76e-06 (9.51e-06 / 3.65e-06)	-0.005 (0.029 / 0.017)	-0.108 (0.193 / 0.664)
3.00E-04	3.00E-04	0.00E+00	0.4	0.5	-0.5	6.18e-06 (1.21e-05 / 4.33e-06)	0.008 (0.032 / 0.018)	0.266 (0.356 / 0.640)
3.00E-04	3.00E-04	0.00E+00	0.4	0.5	-0.75	4.69e-06 (9.03e-06 / 3.33e-06)	-0.006 (0.029 / 0.021)	-0.093 (0.272 / 0.618)
3.00E-04	3.00E-04	1.20E-04	0.4	0	-0.25	1.19e-04 (9.51e-06 / 6.73e-06)	-0.004 (0.015 / 0.027)	-0.012 (0.037 / 0.063)
3.00E-04	3.00E-04	1.20E-04	0.4	0	-0.5	1.14e-04 (9.22e-06 / 1.07e-05)	0.001 (0.016 / 0.023)	0.004 (0.039 / 0.053)
3.00E-04	3.00E-04	1.20E-04	0.4	0	-0.75	8.84e-05 (7.72e-06 / 1.19e-05)	0.004 (0.017 / 0.023)	0.012 (0.043 / 0.061)
3.00E-04	3.00E-04	1.20E-04	0.4	0.5	-0.25	1.19e-04 (9.23e-06 / 1.13e-05)	0.197 (0.016 / 0.028)	0.491 (0.040 / 0.057)
3.00E-04	3.00E-04	1.20E-04	0.4	0.5	-0.5	1.06e-04 (8.56e-06 / 1.08e-05)	0.197 (0.017 / 0.018)	0.494 (0.043 / 0.062)
3.00E-04	3.00E-04	1.20E-04	0.4	0.5	-0.75	8.45e-05 (7.42e-06 / 1.14e-05)	0.210 (0.018 / 0.031)	0.567 (0.046 / 0.113)
3.00E-04	3.00E-04	1.80E-04	0.4	0	-0.25	1.79e-04 (1.19e-05 / 1.01e-05)	-0.005 (0.017 / 0.031)	-0.009 (0.028 / 0.050)
3.00E-04	3.00E-04	1.80E-04	0.4	0	-0.5	1.63e-04 (1.13e-05 / 1.68e-05)	0.011 (0.018 / 0.028)	0.020 (0.030 / 0.047)
3.00E-04	3.00E-04	1.80E-04	0.4	0	-0.75	1.35e-04 (1.02e-05 / 1.51e-05)	-0.017 (0.019 / 0.046)	-0.031 (0.034 / 0.078)
3.00E-04	3.00E-04	1.80E-04	0.4	0.5	-0.25	1.66e-04 (1.12e-05 / 1.37e-05)	0.307 (0.019 / 0.028)	0.517 (0.030 / 0.040)
3.00E-04	3.00E-04	1.80E-04	0.4	0.5	-0.5	1.64e-04 (1.12e-05 / 1.90e-05)	0.302 (0.019 / 0.026)	0.495 (0.030 / 0.053)
3.00E-04	3.00E-04	1.80E-04	0.4	0.5	-0.75	1.27e-04 (9.60e-06 / 1.20e-05)	0.310 (0.022 / 0.033)	0.541 (0.034 / 0.050)
3.00E-04	3.00E-04	2.40E-04	0.4	0	-0.25	2.30e-04 (1.50e-05 / 1.60e-05)	0.022 (0.019 / 0.029)	0.029 (0.024 / 0.038)
3.00E-04	3.00E-04	2.40E-04	0.4	0	-0.5	2.18e-04 (2.03e-05 / 1.32e-05)	-0.009 (0.020 / 0.039)	-0.011 (0.025 / 0.048)

Supplementary Table 4 (continuation).

true_pi1u	true_pi2u	true_pi12	true_h2	true_rg	spow	pi12	rg	rho12
3.00E-04	3.00E-04	2.40E-04	0.4	0	-0.75	1.76e-04 (1.44e-05 / 1.69e-05)	-0.022 (0.021 / 0.041)	-0.027 (0.028 / 0.052)
3.00E-04	3.00E-04	2.40E-04	0.4	0.5	-0.25	2.30e-04 (1.44e-05 / 1.33e-05)	0.396 (0.021 / 0.033)	0.500 (0.024 / 0.039)
3.00E-04	3.00E-04	2.40E-04	0.4	0.5	-0.5	2.04e-04 (1.24e-05 / 2.34e-05)	0.411 (0.020 / 0.026)	0.524 (0.025 / 0.044)
3.00E-04	3.00E-04	2.40E-04	0.4	0.5	-0.75	1.79e-04 (1.28e-05 / 1.26e-05)	0.423 (0.024 / 0.019)	0.546 (0.027 / 0.031)
3.00E-04	3.00E-04	3.00E-04	0.4	0	-0.25	2.76e-04 (1.38e-05 / 1.74e-05)	-0.003 (0.019 / 0.051)	-0.003 (0.021 / 0.054)
3.00E-04	3.00E-04	3.00E-04	0.4	0	-0.5	2.45e-04 (1.31e-05 / 2.19e-05)	0.015 (0.020 / 0.046)	0.016 (0.022 / 0.050)
3.00E-04	3.00E-04	3.00E-04	0.4	0	-0.75	2.03e-04 (1.18e-05 / 1.69e-05)	-0.006 (0.022 / 0.047)	-0.006 (0.024 / 0.050)
3.00E-04	3.00E-04	3.00E-04	0.4	0.5	-0.25	2.74e-04 (2.68e-05 / 1.91e-05)	0.500 (0.042 / 0.040)	0.523 (0.016 / 0.037)
3.00E-04	3.00E-04	3.00E-04	0.4	0.5	-0.5	2.49e-04 (2.05e-05 / 2.15e-05)	0.512 (0.039 / 0.018)	0.543 (0.016 / 0.028)
3.00E-04	3.00E-04	3.00E-04	0.4	0.5	-0.75	2.05e-04 (1.43e-05 / 2.11e-05)	0.523 (0.031 / 0.025)	0.559 (0.018 / 0.033)
3.00E-04	3.00E-04	6.00E-05	0.4	0	-0.25	5.79e-05 (7.12e-06 / 1.25e-05)	0.010 (0.012 / 0.019)	0.043 (0.063 / 0.090)
3.00E-04	3.00E-04	6.00E-05	0.4	0	-0.5	5.53e-05 (6.89e-06 / 6.75e-06)	-0.013 (0.013 / 0.029)	-0.063 (0.066 / 0.143)
3.00E-04	3.00E-04	6.00E-05	0.4	0	-0.75	4.39e-05 (5.74e-06 / 6.94e-06)	0.004 (0.015 / 0.024)	0.023 (0.076 / 0.133)
3.00E-04	3.00E-04	6.00E-05	0.4	0.5	-0.25	5.78e-05 (6.99e-06 / 8.90e-06)	0.104 (0.013 / 0.025)	0.532 (0.072 / 0.137)
3.00E-04	3.00E-04	6.00E-05	0.4	0.5	-0.5	5.20e-05 (6.53e-06 / 9.76e-06)	0.110 (0.014 / 0.021)	0.566 (0.075 / 0.093)
3.00E-04	3.00E-04	6.00E-05	0.4	0.5	-0.75	4.33e-05 (5.83e-06 / 9.58e-06)	0.107 (0.015 / 0.024)	0.557 (0.086 / 0.139)

Supplementary Table 5. Sensitivity analysis: simulations with incomplete reference.

Simulations with incomplete reference showing that total number of causal SNPs, as well as heritability, are estimated correctly, while polygenicity parameter is misestimated. “Frac” column indicates the proportion of SNPs from the reference that were used for LD structure estimation. All phenotypes were simulated using complete reference of N=11015833 variants. “true_ncausal” gives total number of simulated causal variants (including those that were not included in the estimation of LD structure). “hat_ncausal” is calculated as a product of polygenicity (pi) and the effective number of variants in the reference (N*frac). “true_ncausal” and “hat_ncausal” are expressed in 1,000.

true_h2	true_ncausal	frac	h2 (se)	hat_ncausal (se)	sig2_beta (se)	pi (se)
0.1	33	0.125	0.093 (0.005)	40.7 (16.7)	1.058e-05 (4.299e-06)	2.958e-02 (1.213e-02)
0.1	33	0.25	0.104 (0.005)	30.8 (8.6)	1.557e-05 (4.161e-06)	1.120e-02 (3.130e-03)
0.1	33	0.5	0.105 (0.005)	39.1 (13.8)	1.234e-05 (4.198e-06)	7.100e-03 (2.500e-03)
0.1	3.3	0.125	0.095 (0.006)	3.8 (0.5)	1.159e-04 (1.112e-05)	2.738e-03 (3.348e-04)
0.1	3.3	0.25	0.091 (0.006)	3.4 (0.4)	1.216e-04 (1.136e-05)	1.250e-03 (1.511e-04)
0.1	3.3	0.5	0.097 (0.005)	3.7 (0.4)	1.205e-04 (1.077e-05)	6.725e-04 (7.570e-05)
0.1	3.3	1	0.101 (0.005)	3.7 (0.4)	1.272e-04 (1.126e-05)	3.327e-04 (3.676e-05)
0.4	33	0.125	0.375 (0.008)	36.4 (2.3)	4.758e-05 (3.041e-06)	2.642e-02 (1.688e-03)
0.4	33	0.25	0.379 (0.008)	33.8 (2.1)	5.177e-05 (3.229e-06)	1.227e-02 (7.655e-04)
0.4	33	0.5	0.388 (0.008)	34.2 (2.2)	5.222e-05 (3.199e-06)	6.218e-03 (4.052e-04)
0.4	3.3	0.125	0.384 (0.012)	3.5 (0.2)	5.117e-04 (2.248e-05)	2.520e-03 (1.240e-04)
0.4	3.3	0.25	0.397 (0.011)	3.5 (0.2)	5.250e-04 (2.169e-05)	1.267e-03 (5.925e-05)
0.4	3.3	0.5	0.407 (0.011)	3.5 (0.2)	5.339e-04 (2.060e-05)	6.392e-04 (2.808e-05)
0.4	3.3	1	0.420 (0.010)	3.5 (0.1)	5.473e-04 (1.959e-05)	3.218e-04 (1.326e-05)
0.7	33	0.125	0.654 (0.011)	32.6 (1.4)	9.271e-05 (3.734e-06)	2.366e-02 (1.038e-03)
0.7	33	0.25	0.674 (0.011)	31.6 (1.3)	9.837e-05 (3.817e-06)	1.149e-02 (4.842e-04)
0.7	33	0.5	0.685 (0.011)	32.5 (1.3)	9.712e-05 (3.693e-06)	5.907e-03 (2.412e-04)
0.7	3.3	0.125	0.632 (0.017)	3.2 (0.1)	9.049e-04 (3.201e-05)	2.345e-03 (9.066e-05)
0.7	3.3	0.25	0.674 (0.016)	3.3 (0.1)	9.566e-04 (3.030e-05)	1.181e-03 (4.137e-05)
0.7	3.3	0.5	0.686 (0.015)	3.3 (0.1)	9.556e-04 (2.857e-05)	6.012e-04 (2.022e-05)
0.7	3.3	1	0.680 (0.014)	3.2 (0.1)	9.670e-04 (2.696e-05)	2.948e-04 (9.400e-06)

Supplementary Table 6. Summary statistics Metadata

List of GWAS studies used in current manuscript.

Sample size indicate number of cases and controls for dichotomous phenotypes and total GWAS sample for quantitative phenotypes.

Trait	Sample size	Year	Publication
Schizophrenia	33640 / 43456	2014	S. Ripke et al., Biological insights from 108 schizophrenia-associated genetic loci
Bipolar Disorder	20352 / 31358	2017	E. Stahl et al., Genomewide association study identifies 30 loci associated with bipolar disorder
Educational attainment	766345	2018	J.J. Lee et al, Gene discovery and polygenic prediction from a 1.1-million-person GWAS of educational attainment
Height	709706	2018	L. Yengo et al, Meta-analysis of genome-wide association studies for height and body mass index in ~700,000 individuals of European ancestry
Attention-Deficit/Hyperactivity Disorder	19099 / 34194	2018	D. Demontis et al., Discovery Of The First Genome-Wide Significant Risk Loci For ADHD
Autism Spectrum Disorder	18381 / 27969	2018	J. Grove et al., Common risk variants identified in autism spectrum disorder
Major depressive disorder	59851 / 113154	2018	N.R. Wray et al., Genome-wide association analyses identify 44 risk variants and refine the genetic architecture of major depressive disorder
Rheumatoid Arthritis	14361 / 43923	2014	Y. Okada et al., Genetics of rheumatoid arthritis contributes to biology and drug discovery
Inflammatory Bowel Disease	25042 / 34915	2017	K.M. De Lange et al., Genome-wide association study implicates immune activation of multiple integrin genes in inflammatory bowel disease
Crohn's Disease	12194 / 34915	2017	K.M. De Lange et al., Genome-wide association study implicates immune activation of multiple integrin genes in inflammatory bowel disease
Ulcerative Colitis	12366 / 34915	2017	K.M. De Lange et al., Genome-wide association study implicates immune activation of multiple integrin genes in inflammatory bowel disease
Birth Weight	143677	2016	M. Horikoshi et al., Genome-wide associations for birth weight and correlations with adult disease
Waist Hip Ratio	224459	2015	D. Shungin et al., New genetic loci link adipose and insulin biology to body fat distribution
Body Mass Index	339224	2015	A.E. Locke, Genetic studies of body mass index yield new insights for obesity biology

Supplementary Table 7a. Results of bivariate analysis with MiXeR without right-censoring

Results of cross-trait analysis with MiXeR model, with and without right-censoring for genome-wide significant SNPs.
Columns: nc12– estimated number of shared causal variants, reported in 1,000; nc1 (nc2)– estimated number of causal variants, unique to trait1 (trait2), expressed in 1,000; nc12@p9, nc1@p9 and nc2@p9 are adjusted estimates that explain 90% of heritability in the corresponding component; rho_12 – correlation of effect sizes in shared component; rg –genetic correlation; LDSR(2)_rg - genetic correlation from LD Score Regression using MiXeR MAF model; LDSR_rg - estimate of genetic correlation from LD Score Regression using original LDSR MAF model. Parameters are fitted using ca. 1.1M HapMap3 SNPs. MDD: Major Depressive Disorder; ASD: Autism Spectrum Disorder; ADHD: attention deficit hyperactivity disorder; EDU: educational attainment.

trait1	trait2	nc12@p9 (se)	nc1@p9 (se)	nc2@p9 (se)	nc12 (se)	nc1 (se)	nc2 (se)	rho12 (se)	rg (se)	LDSR_rg (se)	LDSR(2)_rg (se)
Schizophrenia	Bipolar Disorder	6.19 (0.99)	2.10 (1.26)	0.21 (0.44)	27.40 (4.37)	9.30 (5.57)	0.92 (1.97)	0.8533 (0.0193)	0.7251 (0.0711)	0.7246 (0.0238)	0.6815 (0.0211)
Schizophrenia	MDD	5.14 (0.67)	3.15 (0.60)	9.77 (7.41)	22.76 (2.98)	13.94 (2.66)	43.24 (32.78)	0.8088 (0.0502)	0.374 (0.0766)	0.3618 (0.0331)	0.3555 (0.0264)
Schizophrenia	ASD	7.15 (1.66)	1.15 (1.71)	0.52 (3.25)	31.62 (7.35)	5.08 (7.57)	2.30 (14.39)	0.2243 (0.03)	0.201 (0.0334)	0.2348 (0.0545)	0.2122 (0.0466)
Schizophrenia	ADHD	4.72 (2.39)	3.58 (2.53)	0.30 (2.33)	20.87 (10.56)	15.83 (11.21)	1.35 (10.29)	0.1922 (0.0317)	0.1405 (0.0694)	0.1304 (0.0402)	0.1243 (0.0362)
Schizophrenia	EDU	8.29 (0.84)	0.00 (0.04)	2.54 (1.02)	36.68 (3.73)	0.02 (0.19)	11.24 (4.50)	0.0715 (0.0152)	0.0625 (0.0138)	0.0791 (0.0218)	0.0626 (0.0196)
Schizophrenia	Height	0.83 (0.10)	7.46 (0.87)	2.29 (0.12)	3.69 (0.46)	33.01 (3.87)	10.15 (0.54)	-0.0446 (0.0599)	-0.0073 (0.01)	-0.0084 (0.0189)	-0.0103 (0.0174)
Bipolar Disorder	MDD	5.35 (2.45)	1.05 (2.39)	9.57 (7.58)	23.66 (10.83)	4.67 (10.58)	42.35 (33.54)	0.6776 (0.4132)	0.3707 (0.2035)	0.3403 (0.0437)	0.3445 (0.0317)
Bipolar Disorder	ASD	5.31 (1.86)	1.09 (1.90)	2.36 (4.11)	23.50 (8.23)	4.83 (8.41)	10.43 (18.18)	0.2171 (0.0885)	0.1646 (0.0545)	0.174 (0.0549)	0.1372 (0.0475)
Bipolar Disorder	ADHD	3.19 (0.78)	3.21 (1.26)	1.83 (0.86)	14.12 (3.47)	14.20 (5.59)	8.10 (3.82)	0.2824 (0.0507)	0.159 (0.0403)	0.1794 (0.0475)	0.1205 (0.0381)
Bipolar Disorder	EDU	5.72 (1.46)	0.68 (1.16)	5.11 (1.58)	25.31 (6.47)	3.01 (5.15)	22.61 (6.97)	0.2777 (0.0514)	0.1908 (0.0358)	0.1879 (0.0227)	0.182 (0.021)
Bipolar Disorder	Height	0.83 (0.11)	5.57 (1.11)	2.29 (0.13)	3.69 (0.48)	24.63 (4.93)	10.15 (0.56)	0.0009 (0.0671)	0.0002 (0.0127)	-0.014 (0.0205)	-0.0152 (0.0221)
MDD	ASD	5.66 (2.52)	9.25 (7.20)	2.00 (2.46)	25.07 (11.16)	40.94 (31.87)	8.86 (10.89)	0.7948 (0.0709)	0.421 (0.1091)	0.4371 (0.0453)	0.4076 (0.0397)
MDD	ADHD	4.87 (2.29)	10.04 (7.79)	0.15 (2.18)	21.56 (10.14)	44.44 (34.49)	0.65 (9.64)	0.9893 (nan)	0.5571 (0.2662)	0.58 (0.0443)	0.536 (0.038)
MDD	EDU	10.79 (1.31)	4.13 (6.99)	0.04 (1.24)	47.75 (5.80)	18.26 (30.93)	0.17 (5.48)	-0.289 (0.0209)	-0.2454 (0.0392)	-0.2233 (0.0322)	-0.2196 (0.0254)
MDD	Height	2.01 (0.13)	12.91 (7.09)	1.12 (0.12)	8.88 (0.58)	57.12 (31.38)	4.95 (0.53)	-0.2109 (0.0567)	-0.062 (0.0213)	-0.0628 (0.0228)	-0.0639 (0.0186)
ASD	ADHD	5.02 (1.11)	2.65 (4.03)	0.00 (0.76)	22.21 (4.90)	11.71 (17.81)	0.01 (3.34)	0.4662 (0.0381)	0.3772 (0.0711)	0.3656 (0.0638)	0.3459 (0.0511)
ASD	EDU	7.33 (2.40)	0.34 (2.43)	3.50 (2.44)	32.42 (10.61)	1.50 (10.76)	15.50 (10.79)	0.2902 (0.0265)	0.2333 (0.0409)	0.2162 (0.0313)	0.2081 (0.0284)
ASD	Height	1.78 (0.13)	5.89 (4.34)	1.35 (0.13)	7.88 (0.59)	26.05 (19.19)	5.96 (0.57)	0.0669 (0.0512)	0.0243 (0.0204)	0.0139 (0.0314)	0.0305 (0.0254)
ADHD	EDU	4.82 (1.18)	0.20 (0.48)	6.01 (1.32)	21.35 (5.24)	0.87 (2.12)	26.57 (5.85)	-0.8578 (0.0283)	-0.5613 (0.084)	-0.5141 (0.0275)	-0.5159 (0.0255)
ADHD	Height	0.67 (0.09)	4.35 (1.19)	2.46 (0.11)	2.97 (0.39)	19.25 (5.25)	10.87 (0.50)	-0.4631 (0.1071)	-0.0783 (0.0226)	-0.0759 (0.0258)	-0.0644 (0.023)
EDU	Height	1.76 (0.11)	9.07 (0.58)	1.37 (0.10)	7.79 (0.48)	40.14 (2.57)	6.05 (0.46)	0.5194 (0.0402)	0.157 (0.0097)	0.1409 (0.0124)	0.138 (0.0116)

Supplementary Table 7b. Results of bivariate analysis with MiXeR with right-censoring

Appearance of the columns is as in Supplementary Table 7a.

trait1	trait2	nc12@p9 (se)	nc1@p9 (se)	nc2@p9 (se)	nc12 (se)	nc1 (se)	nc2 (se)	rho12 (se)	rg (se)	LDSR_rg (se)	LDSR(2)_rg (se)
Schizophrenia	Bipolar Disorder	6.31 (0.98)	2.12 (1.27)	0.07 (0.27)	27.90 (4.35)	9.37 (5.64)	0.31 (1.20)	0.8409 (0.0192)	0.7236 (0.0639)	0.7246 (0.0238)	0.6815 (0.0211)
Schizophrenia	MDD	4.33 (0.57)	4.09 (0.61)	10.68 (7.20)	19.16 (2.51)	18.11 (2.72)	47.27 (31.84)	0.9663 (0.211)	0.3721 (0.1058)	0.3618 (0.0331)	0.3555 (0.0264)
Schizophrenia	ASD	5.85 (1.69)	2.57 (1.78)	1.70 (3.42)	25.89 (7.46)	11.38 (7.89)	7.54 (15.14)	0.2751 (0.0668)	0.2018 (0.0415)	0.2348 (0.0545)	0.2122 (0.0466)
Schizophrenia	ADHD	4.79 (1.08)	3.63 (1.39)	0.23 (0.15)	21.21 (4.79)	16.06 (6.15)	1.00 (0.68)	0.1975 (0.032)	0.1456 (0.0295)	0.1304 (0.0402)	0.1243 (0.0362)
Schizophrenia	EDU	8.41 (0.87)	0.01 (0.02)	2.92 (1.00)	37.23 (3.83)	0.05 (0.11)	12.93 (4.45)	0.0728 (0.0164)	0.0627 (0.0146)	0.0791 (0.0218)	0.0626 (0.0196)
Schizophrenia	Height	1.60 (0.17)	6.83 (0.90)	2.04 (0.18)	7.07 (0.75)	30.20 (3.99)	9.02 (0.80)	-0.0722 (0.0802)	-0.0208 (0.0232)	-0.0084 (0.0189)	-0.0103 (0.0174)
Bipolar Disorder	MDD	5.32 (2.25)	1.06 (2.18)	9.69 (7.50)	23.53 (9.98)	4.67 (9.63)	42.89 (33.19)	0.6788 (0.3446)	0.369 (0.1754)	0.3403 (0.0437)	0.3445 (0.0317)
Bipolar Disorder	ASD	5.25 (1.83)	1.13 (1.88)	2.31 (4.02)	23.22 (8.11)	4.98 (8.32)	10.20 (17.79)	0.218 (0.0893)	0.1649 (0.0543)	0.174 (0.0549)	0.1372 (0.0475)
Bipolar Disorder	ADHD	3.55 (0.88)	2.82 (1.31)	1.47 (0.88)	15.71 (3.88)	12.49 (5.78)	6.50 (3.89)	0.2519 (0.0456)	0.1582 (0.0405)	0.1794 (0.0475)	0.1205 (0.0381)
Bipolar Disorder	EDU	5.93 (1.33)	0.45 (0.91)	5.41 (1.42)	26.22 (5.87)	1.99 (4.05)	23.94 (6.28)	0.2781 (0.0242)	0.1939 (0.0371)	0.1879 (0.0227)	0.182 (0.021)
Bipolar Disorder	Height	1.34 (0.17)	5.04 (1.12)	2.30 (0.20)	5.93 (0.76)	22.28 (4.94)	10.16 (0.88)	-0.0293 (0.1053)	-0.0082 (0.0297)	-0.014 (0.0205)	-0.0152 (0.0221)
MDD	ASD	5.73 (2.57)	9.28 (7.22)	1.82 (2.34)	25.37 (11.36)	41.06 (31.96)	8.05 (10.35)	0.7786 (0.069)	0.4192 (0.1099)	0.4371 (0.0453)	0.4076 (0.0397)
MDD	ADHD	4.87 (2.04)	10.14 (7.70)	0.15 (1.86)	21.55 (9.03)	44.87 (34.09)	0.66 (8.24)	0.9894 (nan)	0.5552 (0.2371)	0.58 (0.0443)	0.536 (0.038)
MDD	EDU	11.16 (1.40)	3.85 (6.89)	0.17 (1.36)	49.39 (6.20)	17.04 (30.49)	0.76 (6.03)	-0.2785 (0.0221)	-0.2383 (0.0373)	-0.2233 (0.0322)	-0.2196 (0.0254)
MDD	Height	2.98 (0.15)	12.03 (7.08)	0.66 (0.03)	13.19 (0.67)	53.24 (31.32)	2.90 (0.15)	-0.2685 (0.0734)	-0.1083 (0.0386)	-0.0628 (0.0228)	-0.0639 (0.0186)
ASD	ADHD	4.93 (1.98)	2.62 (4.32)	0.09 (1.94)	21.82 (8.78)	11.61 (19.14)	0.39 (8.57)	0.472 (0.0387)	0.378 (0.1462)	0.3656 (0.0638)	0.3459 (0.0511)
ASD	EDU	7.45 (2.61)	0.10 (2.17)	3.88 (2.64)	32.97 (11.53)	0.45 (9.62)	17.19 (11.68)	0.2878 (0.0283)	0.2317 (0.0436)	0.2162 (0.0313)	0.2081 (0.0284)
ASD	Height	1.44 (0.21)	6.11 (4.24)	2.19 (0.24)	6.39 (0.94)	27.03 (18.78)	9.69 (1.06)	0.0248 (0.1464)	0.0068 (0.0422)	0.0139 (0.0314)	0.0305 (0.0254)
ADHD	EDU	4.77 (1.12)	0.25 (0.26)	6.57 (1.23)	21.09 (4.95)	1.12 (1.16)	29.07 (5.44)	-0.8832 (0.0309)	-0.558 (0.0717)	-0.5141 (0.0275)	-0.5159 (0.0255)
ADHD	Height	1.14 (0.19)	3.87 (1.18)	2.49 (0.23)	5.07 (0.82)	17.14 (5.21)	11.02 (1.01)	-0.5787 (0.1572)	-0.1551 (0.0398)	-0.0759 (0.0258)	-0.0644 (0.023)
EDU	Height	2.24 (0.39)	9.09 (0.64)	1.39 (0.38)	9.92 (1.71)	40.23 (2.84)	6.16 (1.69)	0.7162 (0.1621)	0.2502 (0.038)	0.1409 (0.0124)	0.138 (0.0116)
Birth Weight	Waist Hip Ratio	0.93 (0.21)	0.37 (0.18)	2.02 (0.45)	4.13 (0.92)	1.65 (0.78)	8.94 (2.00)	-0.1036 (0.0645)	-0.0492 (0.0294)	-0.0581 (0.0378)	-0.0499 (0.0325)
Birth Weight	Body Mass Index	0.76 (0.18)	0.55 (0.17)	4.97 (0.46)	3.34 (0.79)	2.43 (0.74)	21.99 (2.02)	0.4281 (0.1133)	0.1184 (0.0243)	0.1164 (0.0301)	0.1133 (0.0257)
Birth Weight	Height	1.23 (0.16)	0.07 (0.01)	2.40 (0.21)	5.45 (0.71)	0.32 (0.04)	10.64 (0.95)	0.8447 (0.0434)	0.4776 (0.0404)	0.3923 (0.0264)	0.3792 (0.0232)
Waist Hip Ratio	Body Mass Index	2.39 (0.33)	0.56 (0.10)	3.33 (0.53)	10.58 (1.48)	2.48 (0.45)	14.75 (2.37)	0.9995 (nan)	0.5814 (0.0473)	0.5989 (0.0316)	0.5683 (0.0294)
Waist Hip Ratio	Height	1.98 (0.28)	0.97 (0.21)	1.66 (0.32)	8.76 (1.25)	4.31 (0.94)	7.33 (1.41)	-0.1748 (0.0533)	-0.1056 (0.0324)	-0.078 (0.0223)	-0.0652 (0.0206)
Body Mass Index	Height	2.06 (0.15)	3.66 (0.45)	1.57 (0.14)	9.12 (0.67)	16.22 (2.00)	6.97 (0.62)	-0.2738 (0.0518)	-0.1237 (0.0238)	-0.0802 (0.0186)	-0.0837 (0.0174)
Rheumatoid Arthritis	Inflam. Bowel Disease	0.29 (0.06)	0.10 (0.04)	0.30 (0.09)	1.28 (0.28)	0.43 (0.18)	1.31 (0.39)	0.0151 (0.0654)	0.0092 (0.0397)	0.0137 (0.0164)	0.012 (0.0151)
Rheumatoid Arthritis	Crohns Disease	0.26 (0.05)	0.12 (0.05)	0.23 (0.07)	1.16 (0.23)	0.55 (0.21)	1.00 (0.33)	0.0012 (0.069)	0.0007 (0.0412)	-0.0183 (0.0167)	-0.017 (0.0149)
Rheumatoid Arthritis	Ulcerative Colitis	0.30 (0.07)	0.09 (0.05)	0.25 (0.10)	1.31 (0.29)	0.39 (0.22)	1.12 (0.46)	0.0038 (0.0652)	0.0024 (0.0416)	0.0382 (0.0197)	0.0301 (0.0172)
Inflam. Bowel Disease	Crohns Disease	0.52 (0.05)	0.03 (0.06)	0.00 (0.05)	2.31 (0.22)	0.11 (0.27)	0.00 (0.20)	0.9622 (0.0309)	0.9391 (0.0602)	0.9364 (0.0122)	0.9228 (0.0105)
Inflam. Bowel Disease	Ulcerative Colitis	0.55 (0.06)	0.04 (0.07)	0.00 (0.05)	2.43 (0.26)	0.16 (0.31)	0.00 (0.23)	0.9659 (0.0073)	0.9351 (0.0508)	0.9072 (0.0153)	0.8973 (0.0121)
Crohns Disease	Ulcerative Colitis	0.41 (0.05)	0.07 (0.04)	0.14 (0.08)	1.83 (0.21)	0.33 (0.18)	0.60 (0.36)	0.7506 (0.035)	0.5995 (0.047)	0.6496 (0.0424)	0.6246 (0.0332)

Supplementary Table 8. Results of polygenicity comparison between MiXeR and BayesS

General comparison of the polygenicity estimates between MiXeR and BayesS models.
MiXeR columns: pi_vec(se) gives MiXeR-estimated proportion of causal variants in the reference of 9997231 SNPs; nc(se) is the estimated total number of causal variants, expressed in thousands, calculated as pi_vec * 9997231 (including small effects, e.i. not adjusted for 90% heritability); Sample size gives number of participants in a GWAS study that provided summary statistics.
BayesS columns: pi gives BayesS-estimated proportion of causal variants in the reference of 483634 SNPs; nc is the estimated total number of causal variants, espressed in thousands, calculated as pi * 483634; sample size gives the number of individuals from UK Biobank used in BayesS analysis.

MiXeR				BayesS			
**Trait name**	**Sample size**	**pi_vec (se)**	**nc (se)**	**Trait name**	**Sample size**	**pi**	**nc**
Birth Weight	143677	5.77e-04 (7.25e-05)	5.8 (0.72)	Birth weight	71772	0.028	13.5
Body Mass Index	339224	2.53e-03 (1.86e-04)	25.3 (1.9)	BMI	126389	0.093	45
Height	709706	1.38e-03 (3.83e-05)	13.8 (0.38)	Height	126545	0.048	23.2
Educational attainment	766345	4.79e-03 (2.43e-04)	47.9 (2.4)	Educational attainment	125385	0.132	63.8
Major Depressive Disorder	59851 / 113154	6.60e-03 (3.22e-03)	66.0 (32.2)	Depression	107543	0.083	40.1

Supplementary Table 9. Results of univariate analysis with MiXeR, with and without right-censoring

Results of polygenicity analysis with UGMG, with and without right-censoring for genome-wide significant SNPs. Columns: h2 – heritability estimated by UGMG; pi: estimated of polygenicity (pi); sig2_beta – estimate of discoverability; sig2_zero - variance distortion parameter; nc@p9 – estimated number of causal variants explaining 90% of heritability, reported in 1,000; nc – estimated total number of causal variants, reported in 1,000; LDSR(2)_intercept and LDSR(2)_h2 -estimates from LD score regression using MiXeR MAF model; LDSR_intercept and LDSR_h2 – estimates of LD score regression using original LDSR MAF model; AIC (BIC) - results from Akaike and Bayesian information criterion, showing AIC (BIC) calculated for the infinitesimal model minus AIC (BIC) calculated for the causal mixture model; negative value indicates that AIC (BIC) model selection criteria chooses an infinitesimal model over the causal mixture model. MDD: Major Depressive Disorder; ASD: Autism Spectrum Disorder; ADHD: attention deficit hyperactivity disorder; EDU: educational attainment.

trait	h2 (se)	pi_vec (se)	sig2_beta (se)	sig2_zero (se)	nc@p9 (se)	nc (se)	LDSR(2)_ h2 (se)	LDSR(2)_ intercept (se)	LDSR_h2 (se)	LDSR_ intercept (se)	AIC	BIC
Schizophrenia	0.4526 (0.0154)	3.67e-03 (3.76e-04)	5.94e-05 (5.46e-06)	1.1715 (0.0084)	8.3 (0.85)	36.7 (3.8)	0.3469 (0.018)	1.1403 (0.0125)	0.4545 (0.0184)	1.0588 (0.0119)	189	177.5
Bipolar Disorder	0.3433 (0.0179)	2.83e-03 (4.86e-04)	5.84e-05 (9.06e-06)	1.0845 (0.0076)	6.4 (1.1)	28.3 (4.9)	0.257 (0.0161)	1.0777 (0.0093)	0.3633 (0.0183)	1.0208 (0.0101)	59.1	47.7
MDD	0.0786 (0.0048)	6.60e-03 (3.22e-03)	5.74e-06 (2.76e-06)	1.0478 (0.0069)	14.9 (7.3)	66.0 (32.2)	0.0638 (0.0046)	1.0337 (0.0091)	0.0856 (0.0053)	0.995 (0.009)	5.8	-5.7
ASD	0.1817 (0.0151)	3.39e-03 (1.86e-03)	2.58e-05 (1.40e-05)	1.0489 (0.0072)	7.7 (4.2)	33.9 (18.6)	0.1423 (0.0151)	1.04 (0.0097)	0.2028 (0.0176)	1.0084 (0.0096)	6.1	-5.3
ADHD	0.2431 (0.0161)	2.22e-03 (5.16e-04)	5.27e-05 (1.10e-05)	1.0814 (0.0077)	5.0 (1.2)	22.2 (5.2)	0.1882 (0.014)	1.0724 (0.0098)	0.256 (0.0166)	1.0334 (0.0101)	36.2	24.8
EDU	0.1233 (0.0024)	4.79e-03 (2.43e-04)	1.24e-05 (5.53e-07)	1.2117 (0.0087)	10.8 (0.55)	47.9 (2.4)	0.0896 (0.0027)	1.149 (0.0162)	0.1066 (0.0026)	1.0301 (0.0137)	996.9	985.2
Height	0.6950 (0.0120)	1.38e-03 (3.83e-05)	2.42e-04 (6.06e-06)	2.0252 (0.0164)	3.1 (0.09)	13.8 (0.38)	0.429 (0.0207)	2.1116 (0.0458)	0.4552 (0.0193)	1.8969 (0.0452)	19048.1	19036.6
**Analysis with right-sensoring:**												
Schizophrenia	0.4529 (0.0154)	3.73e-03 (3.85e-04)	5.86e-05 (5.44e-06)	1.1709 (0.0084)	8.4 (0.87)	37.3 (3.8)	0.3469 (0.018)	1.1403 (0.0125)	0.4545 (0.0184)	1.0588 (0.0119)	172	160.5
Bipolar Disorder	0.3432 (0.0179)	2.82e-03 (4.83e-04)	5.86e-05 (9.07e-06)	1.0844 (0.0076)	6.4 (1.1)	28.2 (4.8)	0.257 (0.0161)	1.0777 (0.0093)	0.3633 (0.0183)	1.0208 (0.0101)	59.2	47.8
MDD	0.0792 (0.0048)	6.64e-03 (3.22e-03)	5.74e-06 (2.74e-06)	1.0469 (0.0069)	15.0 (7.3)	66.4 (32.2)	0.0638 (0.0046)	1.0337 (0.0091)	0.0856 (0.0053)	0.995 (0.009)	5.8	-5.6
ASD	0.1804 (0.0151)	3.34e-03 (1.83e-03)	2.60e-05 (1.40e-05)	1.0494 (0.0072)	7.5 (4.1)	33.4 (18.3)	0.1423 (0.0151)	1.04 (0.0097)	0.2028 (0.0176)	1.0084 (0.0096)	6.1	-5.2
ADHD	0.2434 (0.0161)	2.22e-03 (5.15e-04)	5.28e-05 (1.10e-05)	1.0813 (0.0077)	5.0 (1.2)	22.2 (5.1)	0.1882 (0.014)	1.0724 (0.0098)	0.256 (0.0166)	1.0334 (0.0101)	35.9	24.5
EDU	0.1225 (0.0023)	5.02e-03 (2.24e-04)	1.18e-05 (5.24e-07)	1.2105 (0.0079)	11.3 (0.51)	50.2 (2.2)	0.0896 (0.0027)	1.149 (0.0162)	0.1066 (0.0026)	1.0301 (0.0137)	783.9	772.3
Height	0.6490 (0.0121)	1.61e-03 (6.51e-05)	1.94e-04 (8.79e-06)	1.9914 (0.0202)	3.6 (0.15)	16.1 (0.65)	0.429 (0.0207)	2.1116 (0.0458)	0.4552 (0.0193)	1.8969 (0.0452)	6304	6292.5
Birth Weight	0.1012 (0.0059)	5.77e-04 (7.25e-05)	8.45e-05 (8.35e-06)	1.0803 (0.0060)	1.3 (0.16)	5.8 (0.72)	0.0729 (0.0061)	1.0795 (0.0088)	0.1021 (0.0065)	1.0418 (0.0088)	392.1	380.5
Waist Hip Ratio	0.0854 (0.0048)	1.31e-03 (1.77e-04)	3.15e-05 (3.43e-06)	0.8922 (0.0056)	3.0 (0.40)	13.1 (1.8)	0.074 (0.0047)	0.8732 (0.0067)	0.0977 (0.0053)	0.8385 (0.0069)	228.8	217.2
Body Mass Index	0.1260 (0.0037)	2.53e-03 (1.86e-04)	2.40e-05 (1.50e-06)	0.7675 (0.0055)	5.7 (0.42)	25.3 (1.9)	0.1091 (0.0054)	0.7249 (0.0084)	0.1296 (0.0054)	0.6738 (0.008)	674.2	662.7
Rheumatoid Arthritis	0.1761 (0.0144)	1.71e-04 (3.10e-05)	4.97e-04 (6.92e-05)	0.9738 (0.0051)	0.39 (0.07)	1.7 (0.31)	0.1298 (0.0179)	0.9724 (0.0081)	0.1893 (0.0237)	0.9511 (0.0076)	453.1	441.5
Inflam. Bowel Disease	0.3380 (0.0173)	2.59e-04 (2.78e-05)	6.29e-04 (5.84e-05)	1.1708 (0.0062)	0.59 (0.06)	2.6 (0.28)	0.222 (0.0231)	1.178 (0.0099)	0.3043 (0.0264)	1.1441 (0.0096)	1183.7	1172.1
Crohns Disease	0.4755 (0.0262)	2.16e-04 (2.56e-05)	1.06e-03 (1.11e-04)	1.1458 (0.0060)	0.49 (0.06)	2.2 (0.26)	0.3374 (0.0399)	1.1476 (0.0099)	0.4653 (0.0491)	1.1145 (0.0095)	1092	1080.4
Ulcerative Colitis	0.3147 (0.0215)	2.43e-04 (3.64e-05)	6.23e-04 (7.30e-05)	1.1213 (0.0059)	0.55 (0.08)	2.4 (0.36)	0.2087 (0.0256)	1.1226 (0.0089)	0.3083 (0.0284)	1.0933 (0.0089)	568.4	556.7
